# Imine-based [2]catenanes in water†
†Electronic supplementary information (ESI) available. See DOI: 10.1039/c7sc04901c


**DOI:** 10.1039/c7sc04901c

**Published:** 2017-12-18

**Authors:** Kenji Caprice, Marion Pupier, Anneli Kruve, Christoph A. Schalley, Fabien B. L. Cougnon

**Affiliations:** a Department of Organic Chemistry , University of Geneva , 30 Quai Ernest Ansermet , 1211 Geneva 4 , Switzerland . Email: Fabien.cougnon@unige.ch; b Institut für Chemie und Biochemie , Freie Universität Berlin , Takustraße 3 , 14195 Berlin , Germany

## Abstract

We report the efficient condensation of imine-based macrocycles from dialdehyde **A** and aliphatic diamines **B*_n_*** in pure water. Within the libraries, we identified a family of homologous amphiphilic [2]catenanes, whose self-assembly is primarily driven by the hydrophobic effect. The length and odd-even character of the diamine alkyl linker dictate both the yield and the conformation of the [2]catenanes, whose particular thermodynamic stability further shifts the overall equilibrium in favour of imine condensation. These findings highlight the role played by solvophobic effects in the self-assembly of complex architectures.

## Abstract 2

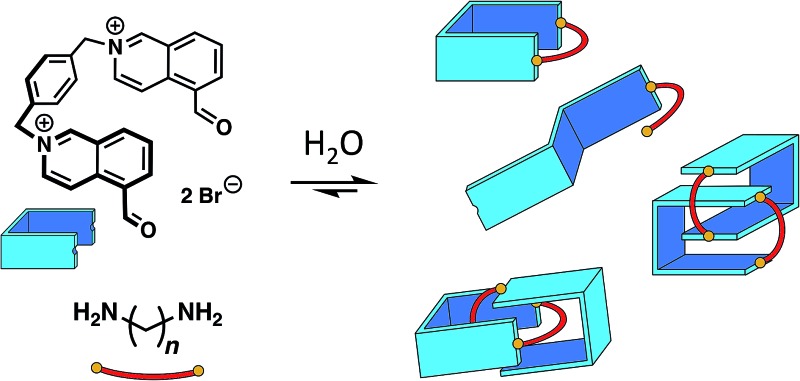
The hydrophobic effect promotes the self-assembly of imine-based [2]catenanes in pure water.

## Introduction

The synthesis of topologically complex molecules continues to challenge chemists,^1^,^2^ prompting the need to develop new efficient synthetic strategies. In the literature, a few examples suggest that solvophobic effects could provide a powerful driving force to direct the assembly of catenanes and knots.^3^–^7^ Most notably, it was serendipitously discovered that amphiphilic macrocycles can adopt non-trivial topologies in water^3^–^5^ in order to minimize their hydrophobic surface area exposed to the solvent. The role of the hydrophobic effect in this process is poorly understood and it is not yet possible to predict the formation of complex topologies in such systems.

**Fig. 1:**
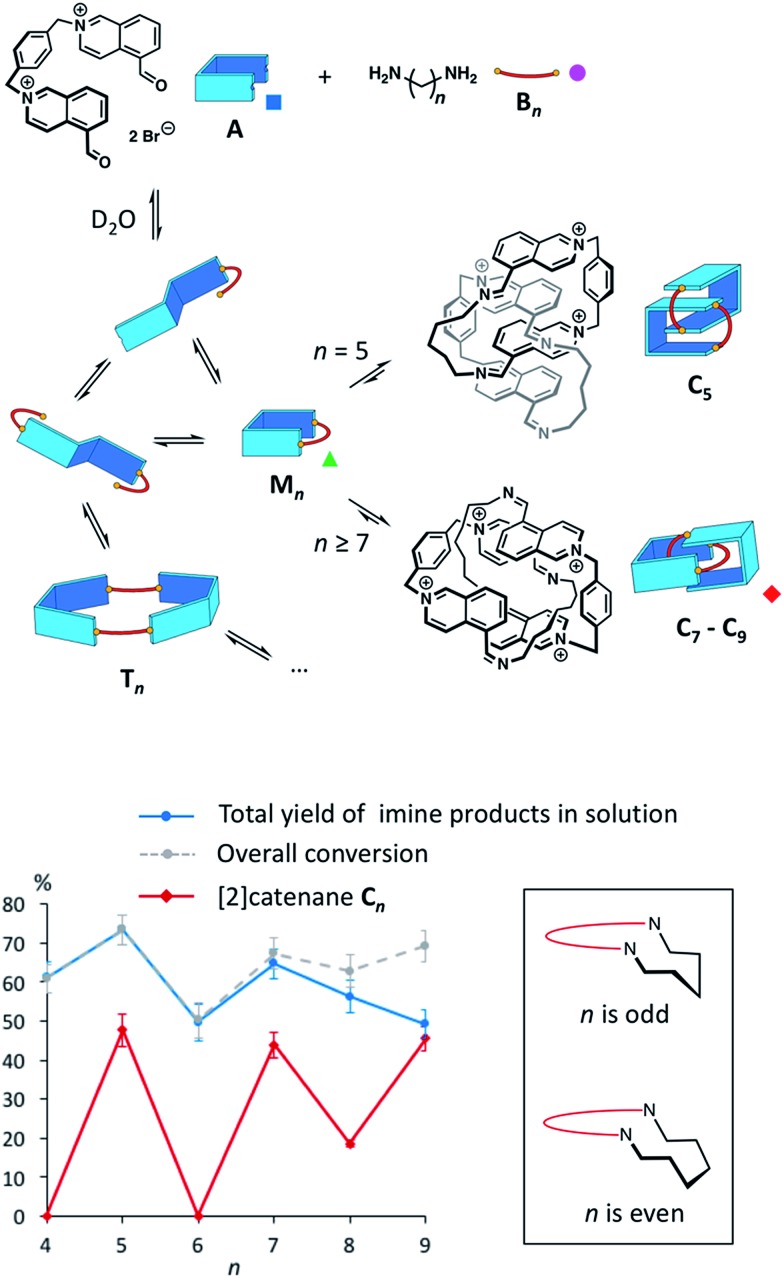
Top: Imine-based dynamic combinatorial libraries in water of dialdehyde **A** and aliphatic diamine **B*_n_*** (10 mM each, *n* = 4–9). Bottom: Overall conversion (grey dotted line), total yield of imine products (blue line) and of [2]catenanes **C*_n_*** (red line) plotted as a function of *n*. These values were measured from the integration of the ^1^H NMR signals (internal standard: hydroquinone). Insert: zigzag representation of odd- and even-numbered aliphatic chains within a macrocycle.

We describe here the dynamic combinatorial^8^ synthesis of imine-based [2]catenanes in water from a dialdehyde (**A**, Fig. 1) and a series of homologous aliphatic diamines **B*_n_*** (*n* = 4 to 9 denotes the number of CH_2_ groups). Building block **A** is composed of two water-soluble, electron-deficient aromatic surfaces (isoquinolinium bromide) connected by a *p*-xylylene linker and terminated by two aldehydes. Closing **A** with a hydrophobic aliphatic diamine provides amphiphilic macrocycles, wherein permanent positive charges alternate with lipophilic units. Our system was specifically designed to probe whether the hydrophobicity of a purely aliphatic chain is sufficient to drive the formation of interlocked structures in the absence of motifs that favour more traditional supramolecular interactions (*e.g.* metal coordination, π–π donor–acceptor interactions or cooperative hydrogen bonds) and in spite of the charge repulsion between the isoquinonium moieties of **A**. In this context, the use of imine condensation presents several advantages. First, aliphatic diamines of various lengths are commercially available, allowing us to study easily the effect of chain length on the product distribution in the dynamic combinatorial libraries. In addition, aliphatic diamines display minimum hydrophilicity after condensation. The reversibility of the imine bond has been extensively used to produce complex supramolecular architectures.^9^ In water, the condensation of an aldehyde and an amine is generally disfavoured^10^,^11^ but can be high yielding in some cases. In nature, a mechanism central to our visual cycle involves the formation of a Schiff base between the retinal chromophore and a lysine residue positioned in the hydrophobic cavity of opsin.^12^ In the laboratory, imines can also be stabilized in water by metal coordination,^13^ within the core of micelles,^14^ or in the presence of an adjacent boronate.^15^ In most other situations, chemists have preferred the related, more stable acyl hydrazones and oximes^16^ over imines. With isoquinolinium-based aldehydes, the imine bond can form rather efficiently in water (condensation yield >60%, Fig. S3–S10†). However, the equilibrium is very sensitive to the experimental conditions. In agreement with the literature,11*a* we preliminarily assessed that imine condensation was most efficient at higher concentrations of building blocks, higher pH and lower temperatures.

## Results and discussion

Dynamic combinatorial libraries were prepared from **A** and each one of the diamines **B*_n_*** (20 mM total, 1 : 1) in water under optimum conditions for imine condensation (room temperature, pH 9.6). The libraries re-organised within a few minutes when conditions were changed (Fig. S7–S10†), preventing us from analysing the libraries by conventional chromatography and to isolate the products formed. Clean imine reduction also proved challenging, mostly due to the presence of the reactive isoquinolinium unit. Therefore, we characterised the products formed directly within the libraries, using ^1^H NMR spectroscopy and electrospray ionization mass spectrometry (ESI-MS), including traveling wave ion-mobility spectrometry (TWIMS).

The libraries reached thermodynamic equilibrium within five minutes and their composition did not evolve any further over a few days. At equilibrium, a significant amount of the initial **A** and **B*_n_*** remained. Nevertheless, the total yield of imine-based products, measured from the integration of the NMR signals using hydroquinone as an internal standard, ranged between 49% and 74% (Fig. 1).

Overall, the yields of imine-based products displayed an odd-even effect^17^ with respect to *n*. Imine yields were lower if *n* was even (61% and 49% for *n* = 4 and 6) and higher if *n* was odd (74% and 65% for *n* = 5 and 7). The odd-even effect was attenuated for longer diamines and the yields slightly decreased (56% and 49% for *n* = 8 and 9). With these longer diamines, a fine precipitate was observed, leading to the disappearance of imine oligomers from the solution. This behaviour explains why conversion and total yield of imine diverge when *n* ≥ 7.

The odd-even effect was also apparent in the library compositions. If *n* was even, the ^1^H NMR spectra were rather complex (Fig. S11†) suggesting the formation of mixtures of multiple oligomers. Several species were observable in the ESI mass spectra of these libraries.

**Fig. 2:**
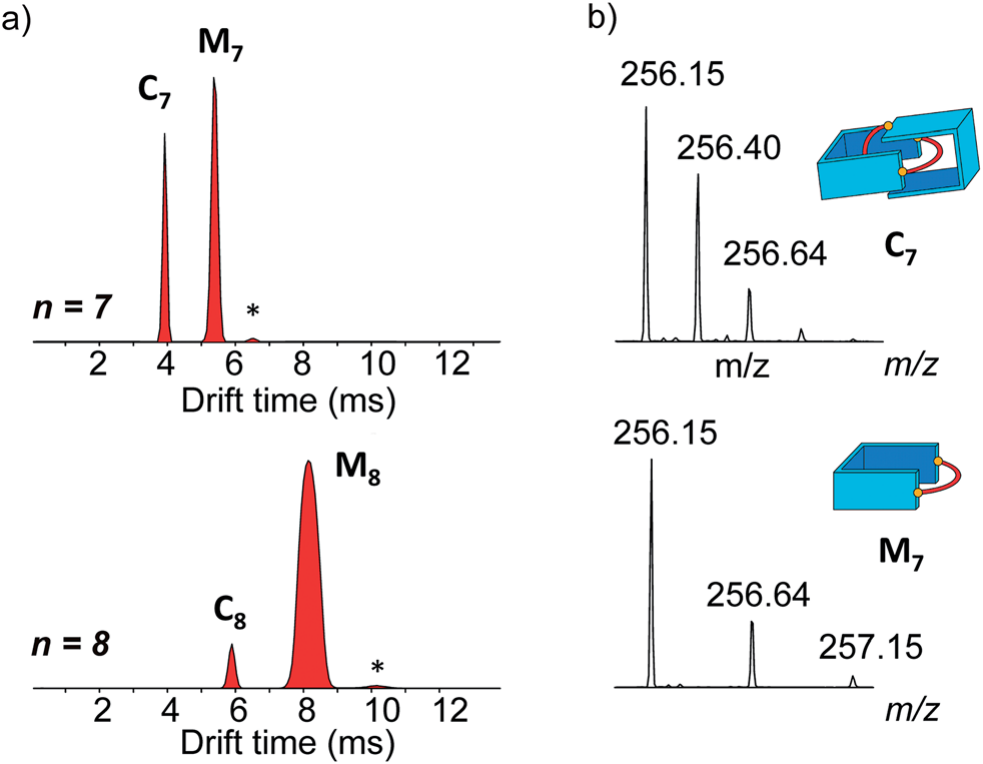
(a) Arrival time distributions for the ions at *m*/*z* 256.15 (*n* = 7) and 263.16 (*n* = 8). The relative abundances of **M*_n_*** and **C*_n_*** between the two cases illustrate the occurrence of an odd-even effect. The asterisk labels a trace of the open form of **M_7_** (top) or **M_8_** (bottom). (b) Isotope patterns of the ion-mobility-separated parent ions **C_7_** and **M_7_** with peak spacings of Δ*m*/*z* = 0.25 and 0.5 in line with the assigned charge states.

The main species corresponded to the closed [1 + 1] macrocycles (**M*_n_***). Only traces of the larger [2 + 2] species (*I*_[2+2]_/(*I*_[1+1]_ + *I*_[2+2]_) < 7%, Fig. 2a) were observed. In contrast, the ^1^H NMR spectra were much simpler if *n* was odd and were clearly dominated by the presence of one major product. In the mass spectra, **M*_n_*** was still observed, but the abundance of **C*_n_*** had significantly increased (*I*_[2+2]_/(*I*_[1+1]_ + *I*_[2+2]_) > 31%, Fig. 2a). From an entropic point of view, the formation of the relatively large [2 + 2] species was intriguing. Therefore, we investigated whether the it could be a [2]catenane (**C*_n_***), composed of two interlocked macrocycles **M*_n_***, rather than a trivial macrocycle (**T*_n_***). The in-source formation of a non-specific complex of two **M*_n_*** was immediately ruled out, as charge repulsion would promote dissociation rather than unspecific association, especially at the concentration at which the mass spectra were recorded (20 μM). For clarity, the results of this investigation are only described here when *n* = 7 as a representative example.

**Fig. 3:**
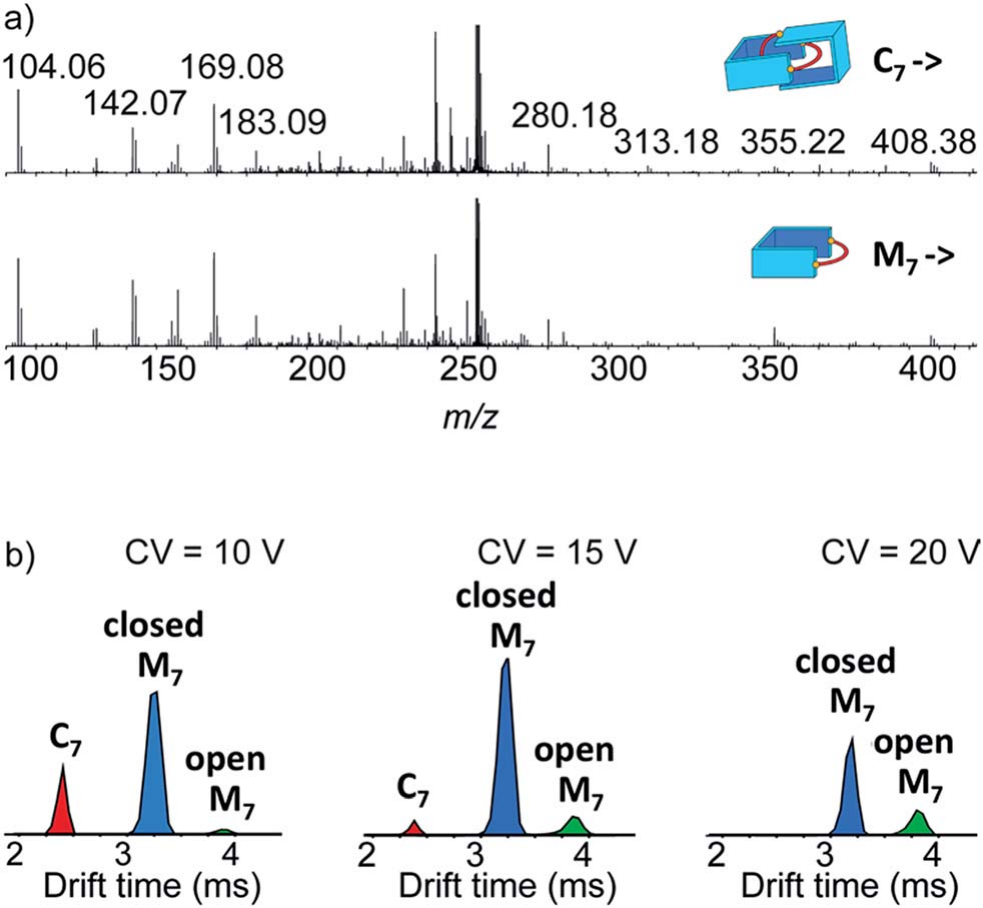
(a) Collision-induced decay spectra of the mass-selected and IMS-separated parent ions **C_7_** and **M_7_** (*m*/*z* 256). (b) Arrival time distribution of the species formed at low, medium and high collision voltage (CV) in the collision-induced decay of the non-separated parent ions **C_7_** and **M_7_** (*m*/*z* 256).

Narrow arrival time distributions (ATDs) for both **M_7_** and the [2 + 2] species were consistent with single molecular structures. Both species were independently subjected to collision-induced decay experiments resulting in virtually identical spectra (Fig. 3a). This result strongly suggested that the [2 + 2] species was the [2]catenane **C_7_**. Initial bond cleavage of **C_7_** led to charge-separation-driven dissociation into one closed and one open [1 + 1] macrocycles, which subsequently fragmented like the simpler **M_7_** and thus gave rise to identical spectra. **T*_n_*** should fragment in a non-symmetrical fashion and its collision-induced decay spectrum should be markedly different from that of **M*_n_***. The collision energy needed to induce the fragmentation of **C_7_** (Fig. S38†) also confirmed its interlocked nature. The acceleration voltages at which 50% of the parent ions have fragmented is significantly lower for **C_7_** (15.5 V) than for **M_7_** (23.0 V) even though the larger **C_7_** ions can store more internal energy before they fragment. This agrees only with a catenated structure, which merely requires a single bond cleavage for the initial fragmentation. Charge repulsion within the quadruply charged [2]catenane further reduces the energetic barrier that must be overcome for cleavage. **T*_n_*** would only fragment when two covalent bonds are broken and would require a higher voltage than **M*_n_***.

Another experiment was independently conducted to confirm our assignment without ambiguity. On the mass spectra of the full library, we selected the molecular ion *m*/*z* = 256, corresponding to both **C_7_** and **M_7_**. We first performed on this specific molecular ion collision-induced decay (CID) at various collision energies. The species produced were subsequently analysed with TWIMS and MS. Three peaks were observed in the mobilogram (Fig. 3b). The first two peaks corresponded to **C_7_** and **M_7_**. The third peak corresponded to another isomer of **M_7_**, as identified by its isotope pattern, which exactly matched that of **M_7_**. Its longer drift time indicated a less compact structure and it could thus be assigned to the open form of **M_7_**, produced by cleavage of one bond *via* in-source fragmentation. Only traces of this species were observed in the previous experiments. As the collision energy increased from 10 V to 20 V, we observed that **C_7_** disappeared while the amount of the open and closed **M_7_** increased (Fig. 3b and S37†). At higher collision energies, **M_7_** also underwent bond cleavage, forming the open **M_7_** and smaller fragments. The cumulated increase of both **M_7_** peaks was directly proportional to the decrease of **C_7_** peak, showing that the fragmentation of **C_7_** cleanly produced the open and closed **M_7_**.

**Fig. 4:**
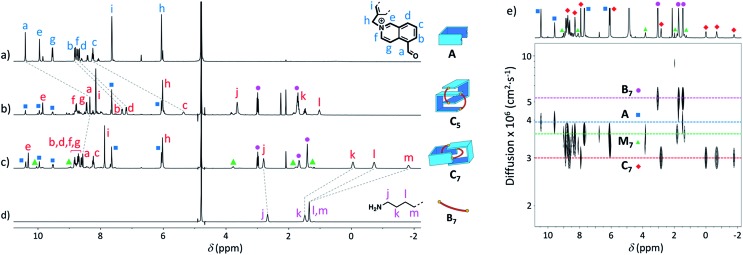
^1^H NMR spectra in D_2_O of (a) **A** (non-labelled signals correspond to the hydrate), (b) library formed from **A** and **B_5_** (10 mM each), (c) library formed from **A** and **B_7_** (10 mM each), and (d) **B_7_**. Only the signals corresponding to the [2]catenanes are assigned in spectra (b and c). (e) DOSY of the library formed from **A** and **B_7_** (5 mM each) in D_2_O. Squares, diamonds and circles refer to the labelling scheme defined in Fig. 1.

**Fig. 5:**
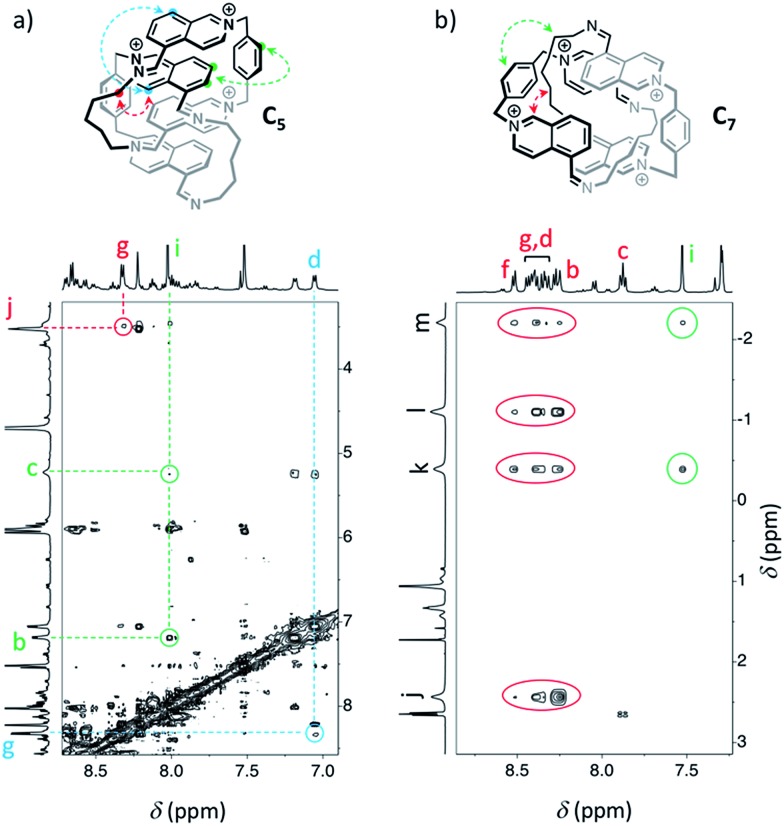
NOESY (D_2_O, 500 MHz, 298 K, *d*_8_ = 500 ms) of the libraries composed of (a) **A** and **B_5_** (5 mM each), and (b) **A** and **B_7_** (5 mM each). Correlations in agreement with the proposed structure of [2]catenanes are highlighted.

Similarly, we demonstrated by ESI-MS that the main products formed from odd-numbered diamines were the [2]catenanes **C_5_**, **C_7_** and **C_9_** (Fig. S42†). Detailed ^1^H NMR analysis of the libraries confirmed the catenated structure of these main products and provided more detailed insight into their binding mode. The spectrum of [2]catenane **C_5_** (Fig. 4b) reflected its interlocked nature. The signals of the aromatic protons b and d were substantially shifted upfield compared to the parent dialdehyde (Δ*δ* ∼ 1.5 ppm). Proton c exhibited the largest shift (Δ*δ* ∼ 3 ppm) and was considerably broadened. These shifts were consistent with a conformation in which the four isoquinolinium units stack in an antiparallel way in order to maximise stacking and minimise charge repulsion. The [2]catenane **C_5_** must be confined into this compact conformation by the short length of the aliphatic chain. The high symmetry reflected in the ^1^H NMR spectrum implied that the inner and outer isoquinolinium units rapidly exchange on the NMR time-scale by circumrotation of the two wheels. NOE cross-peaks (Fig. 5a) between the stacking isoquinoliniums (g ↔ d) and their neighbouring xylylene (b, c ↔ i) and aliphatic (g ↔ j) moieties correlated well with the proposed structure.

The [2]catenanes bearing longer aliphatic chains (*n* ≥ 7) displayed significantly different spectra. The [2]catenane **C_7_** (Fig. 4c) is described here as a characteristic example. All the aromatic protons of **C_7_** resonated in the same range as that of the parent dialdehyde **A** (7.5–10.3 ppm). However, the aliphatic protons k, l and m, located around 1.3–1.5 ppm in the parent diamine, were significantly shifted upfield, even to negative ppm values (0 to –2 ppm). The [2]catenane **C_7_** must therefore adopt a different conformation, wherein the aliphatic chain is threaded between the isoquinolinium units of the other wheel. This conformation is thermodynamically more stable because the four permanent positive charges are located further apart in space, thus minimizing their repulsion. In agreement with this proposed structure, NOE correlations confirmed the close proximity in space of the inner aliphatic and the outer aromatic protons (Fig. 5b). In comparison, the topologically trivial [1 + 1] macrocycle **M_7_**, clearly identifiable in the ^1^H NMR of the same library (triangles, Fig. 4c), did not present any deviations from the expected chemical shift values. Diffusion-ordered spectroscopy (DOSY, Fig. 4e) further confirmed our assignment of the library members. The smallest species of the library was **B_7_** (diffusion coefficient *D* = 5.4 × 10^–6^ cm^2^ s^–1^), followed by **A** (3.9 × 10^–6^ cm^2^ s^–1^), **M_7_** (3.3 × 10^–6^ cm^2^ s^–1^), and finally the larger **C_7_** (3.0 × 10^–6^ cm^2^ s^–1^). As expected, the size of **C_7_** was comparable to that of **C_5_** (3.0 × 10^–6^ cm^2^ s^–1^, Fig. S20†).

**Fig. 6:**
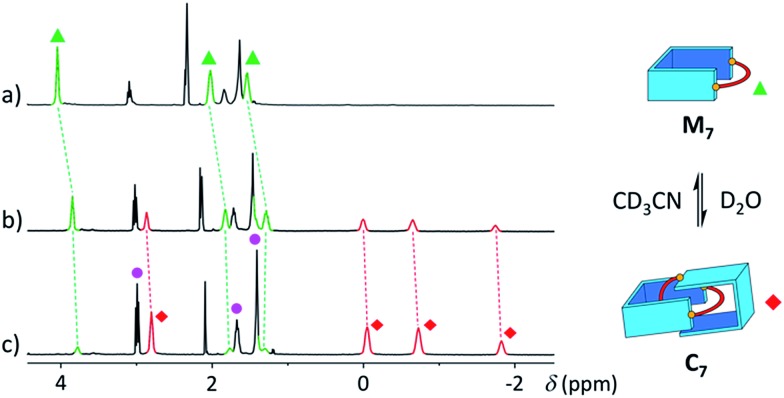
Partial ^1^H NMR spectra of the library formed from **A** and **B_7_** (10 mM each) in (a) 30%, (b) 10%, and (c) 0% of CD_3_CN in D_2_O, showing the reversible polarity-dependent conversion of **C_7_** into **M_7_**.

With a purely aliphatic chain threaded between the aromatic units, **C_7_** exhibits a rather uncommon structure.^4^ The formation of such a [2]catenane appears to be mostly driven by the hydrophobic effect, even if the contribution of other supramolecular interactions cannot be excluded. To confirm this hypothesis, we performed an incremental addition of CD_3_CN^3^,^5^ from 0% to 30% to the aqueous library containing **C_7_** (Fig. 6). The presence of a co-solvent with a lower dielectric constant (*D*_CH_3_CN_ = 37.5) than water (*D*_H_2_O_ = 78.4) alters the hydrophobic effect and shifts the equilibrium in favour of **M_7_**. The kinetics of equilibration were also affected by the presence of a co-solvent and equilibration time significantly increased with the proportion of acetonitrile (>2 h in 30% CD_3_CN). Similarly, all the [2]catenanes disappeared upon incremental addition of CD_3_CN or CD_3_OD (Fig. S54–S60†), a solvent closer to D_2_O in term of its hydrogen bonding ability (*D*_CH_3_OH_ = 32.7). This latter experiment highlights the specific properties of water in the context of this study. Finally, the choice of the counterion (Cl^–^, Br^–^ or CF_3_SO_3_^–^) did not affect the library distributions (Fig. S15, S24, S26, S36, S43 and S52†), showing that the assembly was not templated by a specific counterion, as observed for other catenanes and more complex interlocked structures.^18^

## Conclusions

The self-assembly of amphiphilic imine-based [2]catenanes in water is mostly driven by the need to minimize the hydrophobic surface area exposed to water. It does not require the complementary use of metal coordination^6^ or donor–acceptor π–π stacking.^4^,^5^,^19^ Even the charge repulsion between the positively charged motifs is overcome by the hydrophobic effect.

The products of the libraries could not be isolated, but extensive NMR studies and tandem MS experiments, together with ion mobility spectrometry, allowed for the unambiguous identification and characterisation of the [2]catenanes within the libraries. More importantly, our system generated unexpected results regarding the complex role played by the length of the aliphatic chain. Indeed, the length of the aliphatic diamine controls both the yield and the conformation of the [2]catenanes. Evidently, odd-numbered aliphatic chains favour, and even-numbered chains disfavour, [2]catenane formation. Odd-even effects are often observed in solid phase,^17^ but are much less common in solution.^4^,^20^ Within the [2]catenanes, the aliphatic chains cannot adopt a fully relaxed zigzag conformation. Folding of odd-numbered chains (Fig. 1, insert), required to close the [2]catenanes, induces a chair-like turn that resembles most a relaxed zigzag conformation. On the other hand, folding of even-numbered chains generates unavoidable gauche interactions. In dynamic combinatorial libraries, the final equilibrium distribution can reflect clearly such small energetic differences^8^ (3.9 kJ mol^–1^ per gauche interaction).

The formation of the [2]catenanes is rather remarkable considering that imine condensation is not favoured in pure water, especially under dilute conditions.^11^ The presence of [2]catenanes systematically correlated with an increase in the overall yield of imine (Fig. 1), because the higher thermodynamic stability of the [2]catenanes shifts the equilibria in favour of imine condensation. The system presented here is still quite simple but allowed us to probe how solvophobic effects can direct the formation of interlocked molecules. The knowledge gained from this study will help constructing topologically more complex architectures, such as knots or interwoven materials, using the hydrophobic effect.

## Conflicts of interest

There are no conflicts to declare.

## Supplementary Material

Supplementary information
