# Supplementary material for: Imine-based [2]catenanes in water
Source: Chem Sci. 2017 Dec 18;9(5):1317–22. doi: 10.1039/c7sc04901c (PMC5887103; doi:10.1039/c7sc04901c)
Supplement: Supplementary file 1 [file SC-009-C7SC04901C-s001.pdf]

Electronic Supplementary Material (ESI) for Chemical Science.
This journal is © The Royal Society of Chemistry 2017

Supporting Information for:

Imine-based [2]catenanes in water

*Kenji Caprice,[a] Marion Pupier,[a] Anneli Kruve,[b] Christoph Schalley[b] and Fabien B. L. Cougnon[a]**

[a] *Department of Organic Chemistry, University of Geneva, 30 Quai Ernest-Ansermet 1211 Geneva 4, Switzerland*
[b] *Institut für Chemie und Biochemie, Freie Universität Berlin, Takustraße 3, 14195 Berlin, Germany*

E-mail: fabien.cougnon@unige.ch

Table of Contents

1	Methods	S3
2	Synthesis of the building blocks	S4
2.1	Synthesis of A	S4
2.2	Synthesis of A1	S7
3	Preliminary experiments: determination of the optimum conditions for imine condensation in water	S9
3.1	Imine bond formation in water	S9
3.2	Reversibility of the imine bond in water	S12
3.3	Influence of concentration, number of equivalents of diamine, pD and temperature on imine formation	S13
4	Overall comparison of the libraries composed of A^{2+} and B_n	S17
5	Library composed of A and B_4	S20
6	Library composed of A and B_5	S22
7	Library composed of A and B_6	S31
8	Library composed of A and B_7	S33
9	Library composed of A and B_8	S47
10	Library composed of A and B_9	S49
11	Re-organisation of the aqueous libraries containing [2]catenanes upon addition of organic solvents	S58
12	Deuterium exchange occurring on the isoquinolinium unit in D_2O	S64
13	Semi-empirical calculations	S66

1 Methods

Building block synthesis. All reagents and solvents were purchased from commercial sources and were used without further purification. Reagents for synthesis were purchased from Sigma-Aldrich (isoquinoline-5-carboxaldehyde, benzyl bromide, butylamine, ethanolamine, α,α'-dichloro-*p*-xylene, silver methanesulfonate, aliphatic diamines $\mathbf{B}_n$) and Lancaster (α,α'-dibromo-*p*-xylene).

General procedure for the preparation of the libraries. Unless otherwise stated, standard libraries were prepared to obtain a final concentration of 10 mM of **A** and 10 mM of $\mathbf{B}_n$. A volume of 70 µL of a stock solution of a chosen diamine $\mathbf{B}_n$ (100 mM) in D_2O was added to 630 µL of a stock solution of dialdehyde building block **A** (11.1 mM) in D_2O. The libraries were vigorously shaken for a few seconds. When a small amount of fine precipitate could be observed, it was filtered through a 0.2 µm filter and the pD was measured (pD = 9.6). NMR spectra can be recorded immediately after preparation and the libraries do not evolve over the course of several days.

NMR analysis. Spectra were measured using a Bruker Avance III HD-Nanobay 400 MHz spectrometer, equipped with a 5-mm Prodigy CryoProbe, or using a Bruker Avance III 500 MHz spectrometer equipped with a 5-mm DCH ^{13}C-^{1}H/D helium-cooled cryogenic probe, using D_2O (ARMAR isotope) as the NMR solvent. All signals were internally referenced to the solvent residue (D_2O, δ = 4.79 ppm). For some experiments, CD_3CN (Cambridge Isotope Laboratories) and CD_3OD (ARMAR isotope) served as co-solvents.

IMS analysis. MS, IMS and IMS separated MS/MS spectra were measured on a Synapt G2-S HDMS (Waters Co., Milford, MA, USA) instrument using travelling wave technique. All measurements were performed in ESI positive mode. Soft ionization conditions were used throughout the experiments: Capillary Voltage 700 V, Source temperature 30 ^{o}C, Sampling Cone Voltage 10 V, Source Offset 10 V, Desolvation Gas Temperature 25 ^{o}C and Flow 1.33 L·min^{-1}, Nebulizer Gas Pressure 2.5 bar. MS/MS experiments were carried out is two different modes: the fragmentation was carried out (1) before or (2) after IMS cell.
Before analysis, the library was diluted in pure acetonitrile, so that the final concentration was 20 µM. Under these conditions the sample proved to be stable for approximately 24 h. Acetonitrile is a non-protic solvent, not well suited to promote imine exchange and hydrolysis. By taking a small sample of a concentrated water solution and putting it into pure acetonitrile (reaching a final solvent mixture of ca. 0.2% water in acetonitrile), the exchange almost completely shuts off and the product distribution initially present in the water solution thus becomes frozen. Indeed, the kinetics of equilibration already slows down when the ratio acetonitrile/water increases from 0% (equilibration within 5 min) to 30% (equilibration > 120 min, see Fig. S57).
This solution was infused to the ESI source with a flow rate of 10 µL·min^{-1}.

2 Synthesis of the building blocks

2.1 Synthesis of A

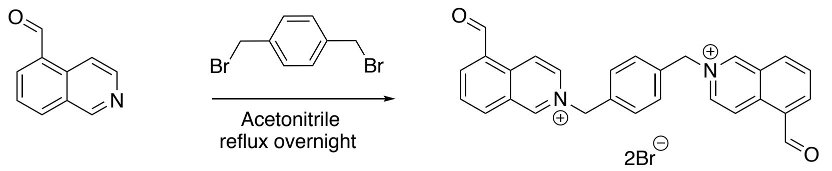

A

A mixture of isoquinoline-5-carboxaldehyde (200 mg, 1.28 mmol) and α,α'-dibromo-*p*-xylene (168 mg, 0.64 mmol) in acetonitrile (5 ml) was refluxed overnight. The precipitate was collected by filtration, washed with small volumes of acetonitrile (10 mL total) and dried under vacuum. Yield: 365 mg, 50%. m. p.: 306 ºC (decomposition). HR-MS (ESI+) calculated for $C_{28}H_{22}N_2O_2^{2+}$ $[M]^{2+}$ (*m/z*) 209.0835, found 209.0839.

NMR characterization was performed in D_2O. Both the dialdehyde **A** and its dihydrate form **A'** were observed in a ratio of **A**: **A'** = 4:1.

NMR characterization of A. ^{1}H NMR (400 MHz, D_2O, 298K) δ (ppm): 10.32 (s, 1H), 9.87 (s, 1H), 9.46 (d, *J* = 7.2 Hz, 1H), 8.74 (d, *J* = 7.2 Hz, 1H), 8.67 (d, *J* = 7.1 Hz, 1H), 8.61 (d, *J* = 8.4 Hz, 1H), 8.17 (dd, *J* = 8.4, 7.2 Hz, 1H), 7.56 (s, 2H), 5.98 (s, 2H). ^{13}C NMR (125 MHz, D_2O, 298K) δ (ppm): 194.58, 149.75, 145.59, 136.58, 135.33, 134.32, 131.11, 130.06, 129.96, 128.49, 123.84, 87.94, 63.91.
NMR characterization of A'. ^{1}H NMR (400 MHz, D_2O, 298K) δ (ppm): 9.76 (s, 1H), 8.70 (d, *J* = 7.2 Hz, 1H), 8.52 (d, *J* = 7.2 Hz, 1H), 8.33 (d, *J* = 7.9 Hz, 2H), 7.99 (t, *J* = 7.9 Hz, 1H), 7.56 (s, 2H), 6.62 (s, 1H), 5.98 (s, 2H). ^{13}C NMR (125 MHz, D_2O, 298K) δ (ppm): 149.68, 137.20, 134.57, 134.04, 133.92, 131.09, 129.97, 128.49, 123.79, 87.94, 63.61.

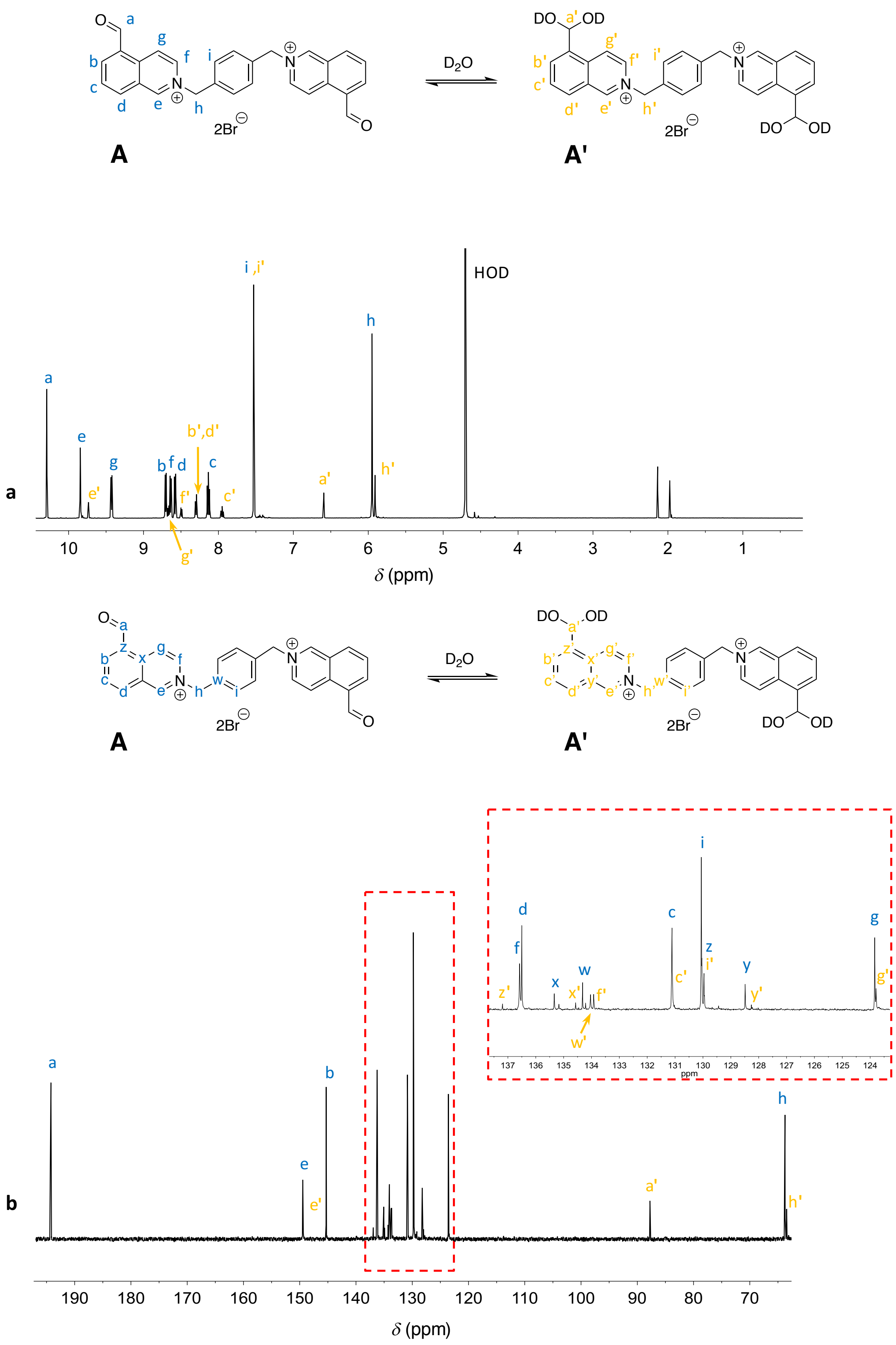

Figure S1. (a) ^{1}H NMR spectrum (D_2O, 400 MHz, 298 K) and (b) ^{13}C NMR spectrum of **A** and **A'** (D_2O, 125 MHz, 298 K). The red insert is an expansion of the aromatic region of the ^{13}C NMR spectrum.

Building block **A** was also synthesized with two other counterions (chloride and methanesulfonate), as described below.

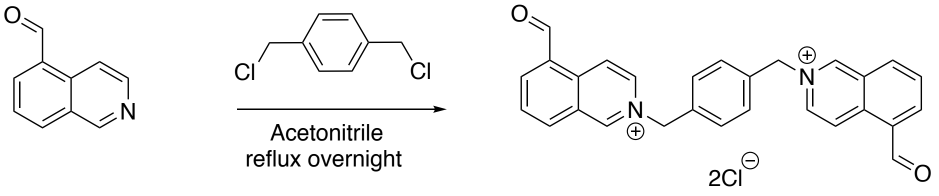

A mixture of isoquinoline-5-carboxaldehyde (200 mg, 1.27 mmol) and α,α'-dichloro-*p*-xylene (112 mg, 0.64 mmol) in acetonitrile (5 mL) was refluxed overnight. The precipitate was collected by filtration, washed with acetonitrile (10 mL) and dried. Yield: 227 mg, 54%. The ^{1}H NMR spectrum of the product obtained is identical to that shown in Figure S1.

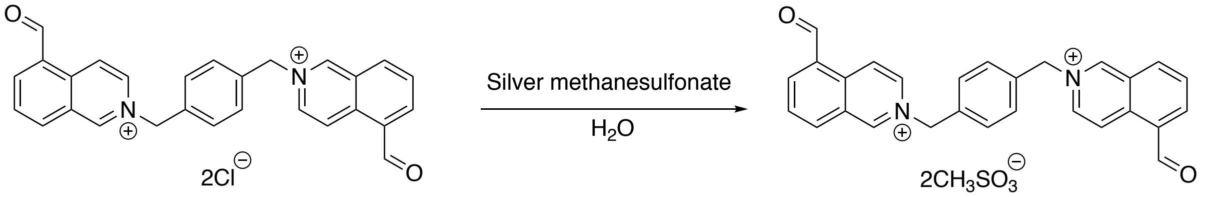

The product synthesized above (**A**·2Cl^{-}; 42 mg, 86 mmol) was solubilized in 500 μL of water. A concentrated solution of silver methanesulfonate (38 mg, 189.42 mmol) in water (500 μL) was added. A precipitate (silver choride) formed immediately and the reaction was left to proceed for 1 hour. The mixture was centrifuged and the precipitate was discarded. The aqueous phase was lyophilised and the residue was washed several times with small amounts of acetonitrile (3 x 200 μL) to remove the small excess of silver methanesulfonate remaining. After washing, the product was dried under vacuum. Yield: 205 mg, 80%.
The ^{1}H NMR spectrum of the product obtained is identical to that shown in Figure S1.

2.2 Synthesis of A1

A monoaldehyde **A1'** was synthesized to test the formation of the imine bond in water (see following Fig. S3-6)

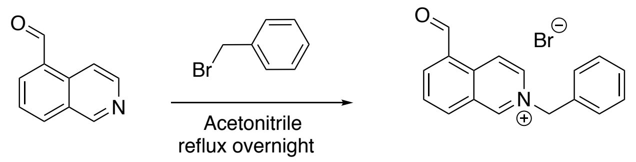

A1

A mixture of isoquinoline-5-carboxaldehyde (50 mg, 0.32 mmol) and benzyl bromide (54 mg, 0.32 mmol) in acetonitrile (5 mL) was refluxed overnight. The precipitate was collected by filtration, washed with small volumes of acetonitrile (10 mL total) and dried under vacuum. Yield: 56 mg, 54%. m. p.: 201 ºC. HR-MS (ESI+) calculated for $C_{17}H_{14}NO^+$ $[M]^+$ (*m/z*): 248.1070, found 248.1073.

NMR characterization was performed in D_2O. Both the dialdehyde **A1** and its dihydrate form **A1'** were observed in a ratio **A1**: **A1'** = 5:1.

NMR characterization of A1. ^{1}H NMR (400 MHz, D_2O, 298K) δ (ppm): 10.32 (s, 1H), 9.83 (s, 1H), 9.43 (d, *J* = 5.6 Hz, 1H), 8.72 (d, *J* = 6.9 Hz, 1H), 8.67 (d, *J* = 4.3 Hz, 1H), 8.59 (d, *J* = 7.9 Hz, 1H), 8.24 – 8.08 (t, 1H), 7.47 (s, 5H), 5.92 (s, 2H). ^{13}C NMR (125 MHz, D_2O, 298K) δ (ppm): 194.59, 149.50, 136.58, 136.49, 135.28, 132.54, 130.07, 129.95, 129.54, 129.17, 128.46, 123.67.
NMR characterization of A1'. ^{1}H NMR (400 MHz, D_2O, 298K) δ (ppm): 9.70 (s, 1H), 8.69 (d, *J* = 6.6 Hz, 1H), 8.66 (d, *J* = 6.6 Hz, 1H), 8.51 (d, *J* = 8.3 Hz,21H), 8.31 (d, *J* = 8.4 Hz, 1H), 7.97 – 7.93 (t, 1H), 7.43 (s, 5H), 6.60 (s, 1H), 5.86 (s, 2H). ^{13}C NMR (125 MHz, D_2O, 298K) δ (ppm): 194.59, 149.50, 136.58, 136.49, 135.28, 132.54, 130.07, 129.95, 129.54, 129.17, 128.46, 123.67.

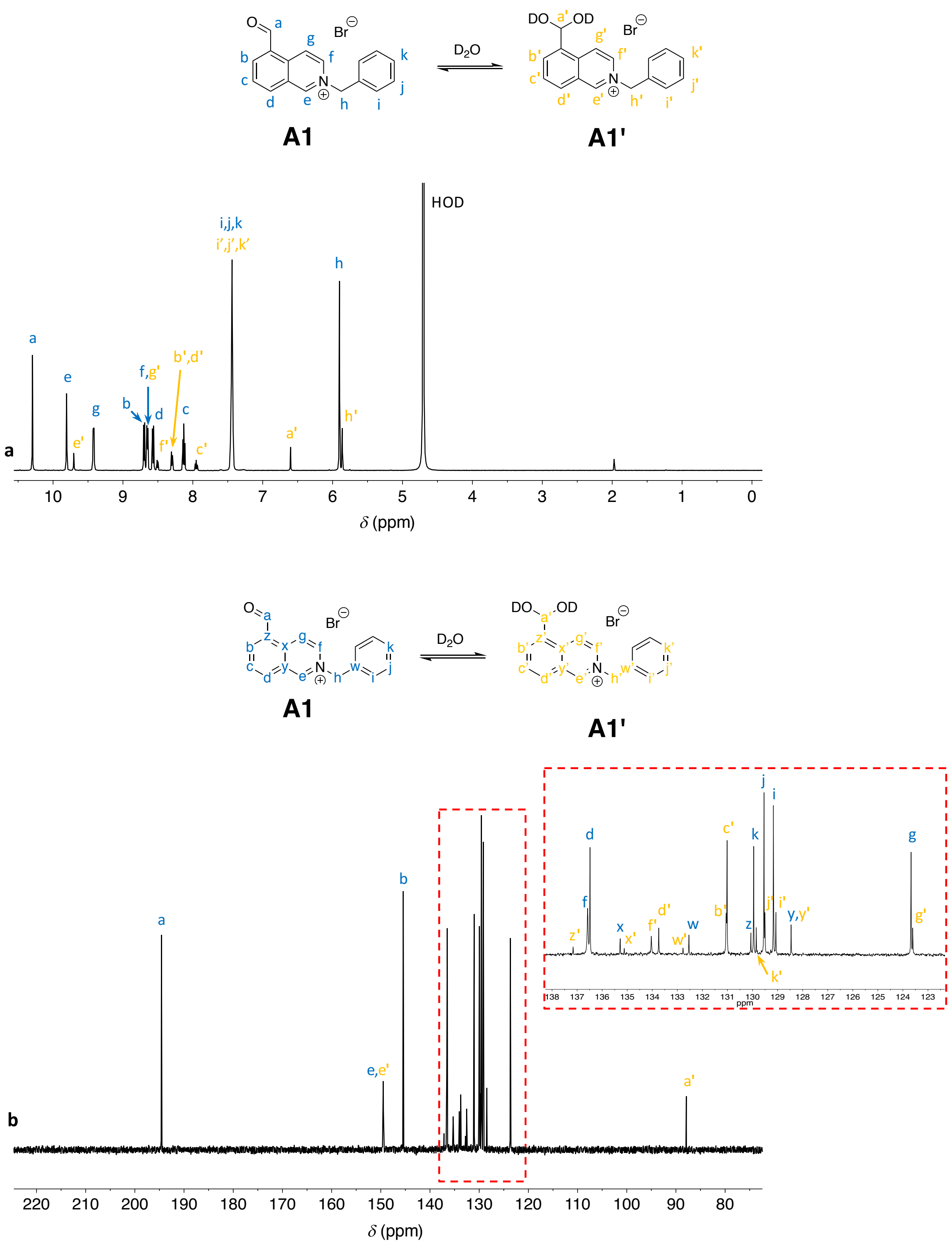

Figure S2. (a) ^{1}H NMR (D_2O, 400 MHz, 298 K) and (b) ^{13}C NMR spectra of **A1** and **A1'** (D_2O, 100 MHz, 298 K). The red insert is an expansion of the aromatic region of the ^{13}C NMR spectrum.

3 Preliminary experiments: determination of the optimum conditions for imine condensation in water

3.1 Imine bond formation in water

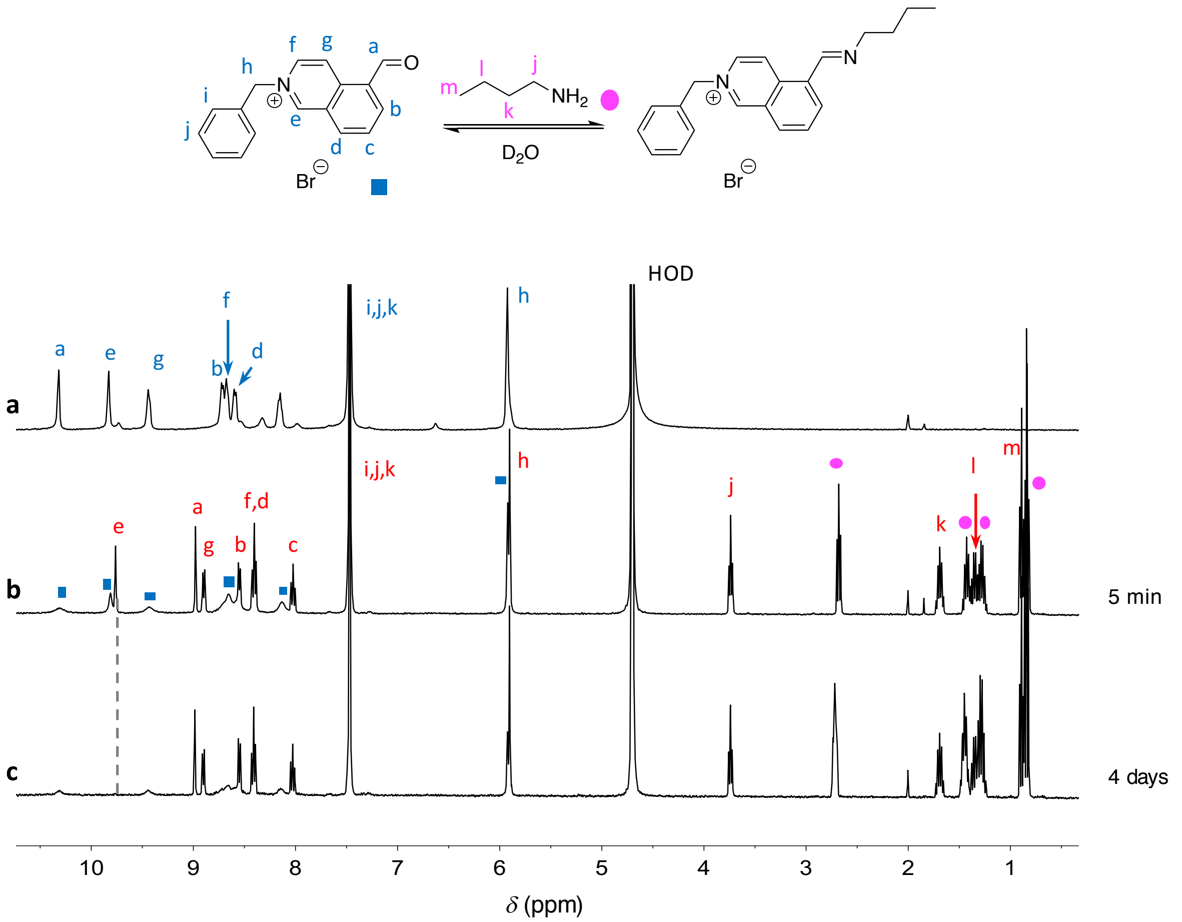

Figure S3. The imine bond forms readily in water and the composition of library does not evolve in the course of several hours. ^{1}H NMR spectra (D_2O, 400 MHz, 298 K) of **A1 (a)** alone, **(b)** 5 min after addition of butylamine, and **(c)** 4 days after addition of butylamine. The initial concentrations are 10 mM of aldehyde and 10 mM of amine. In spectra **(b)** and **(c)** only the protons of the imine product were assigned. Residual starting materials are labelled with blue squares (aldehyde) and pink circles (amine).

The grey dotted line highlights the disappearance over time of the signal corresponding to proton **e**, due to deuterium exchange, as demonstrated in Figures S61-62.

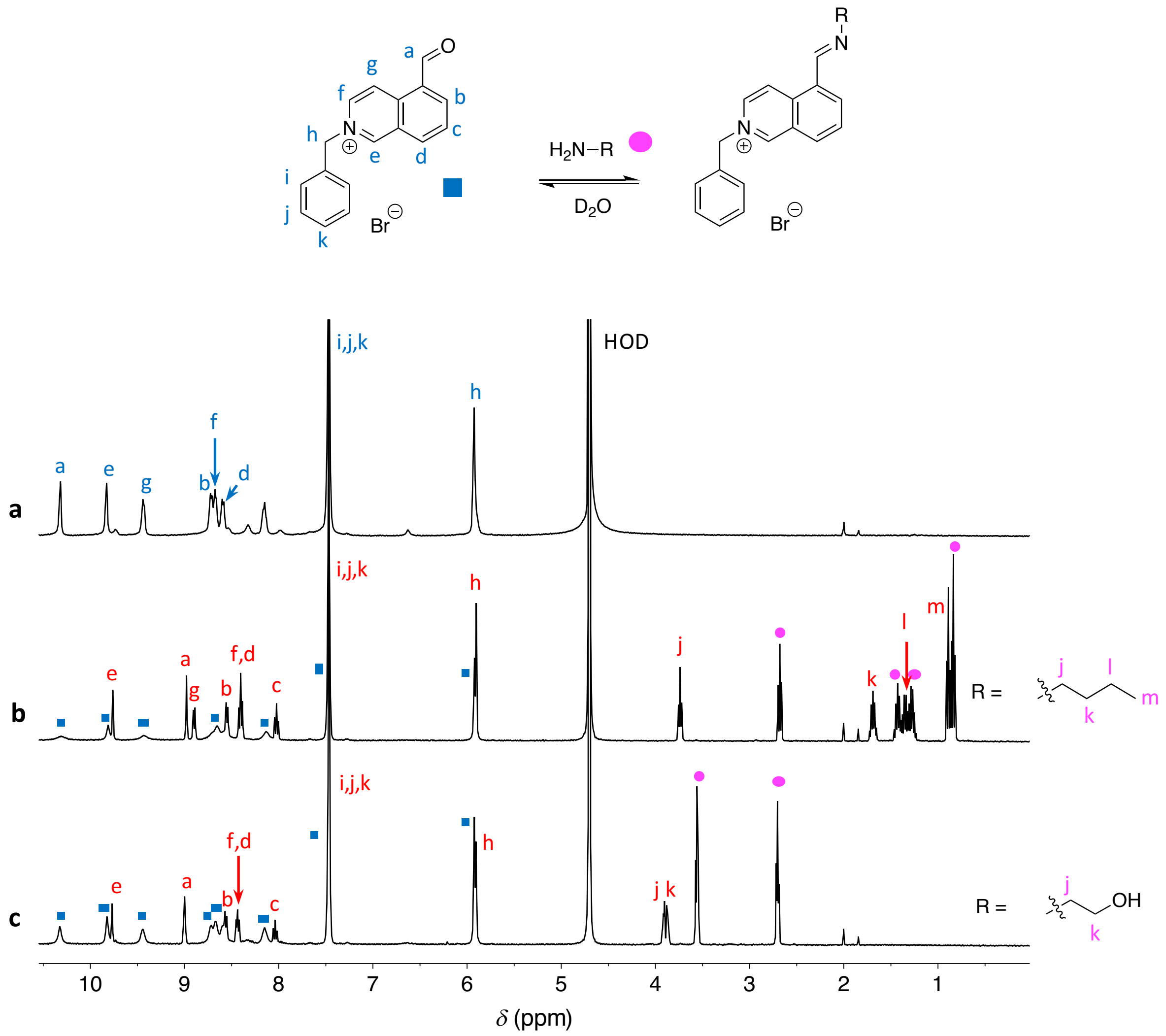

Figure S4. The imine bond forms more efficiently with lipophilic amines. ^{1}H NMR spectra (D_2O, 400 MHz, 298 K) of **A1** **(a)** alone, **(b)** after addition of butylamine, and **(c)** after addition of ethanolamine. The initial concentrations are 10 mM of aldehyde and 10 mM of amine. In spectra **(b)** and **(c)** only the protons of the imine products were assigned. Residual starting materials are labelled with blue squares (aldehyde) and pink circles (amine).

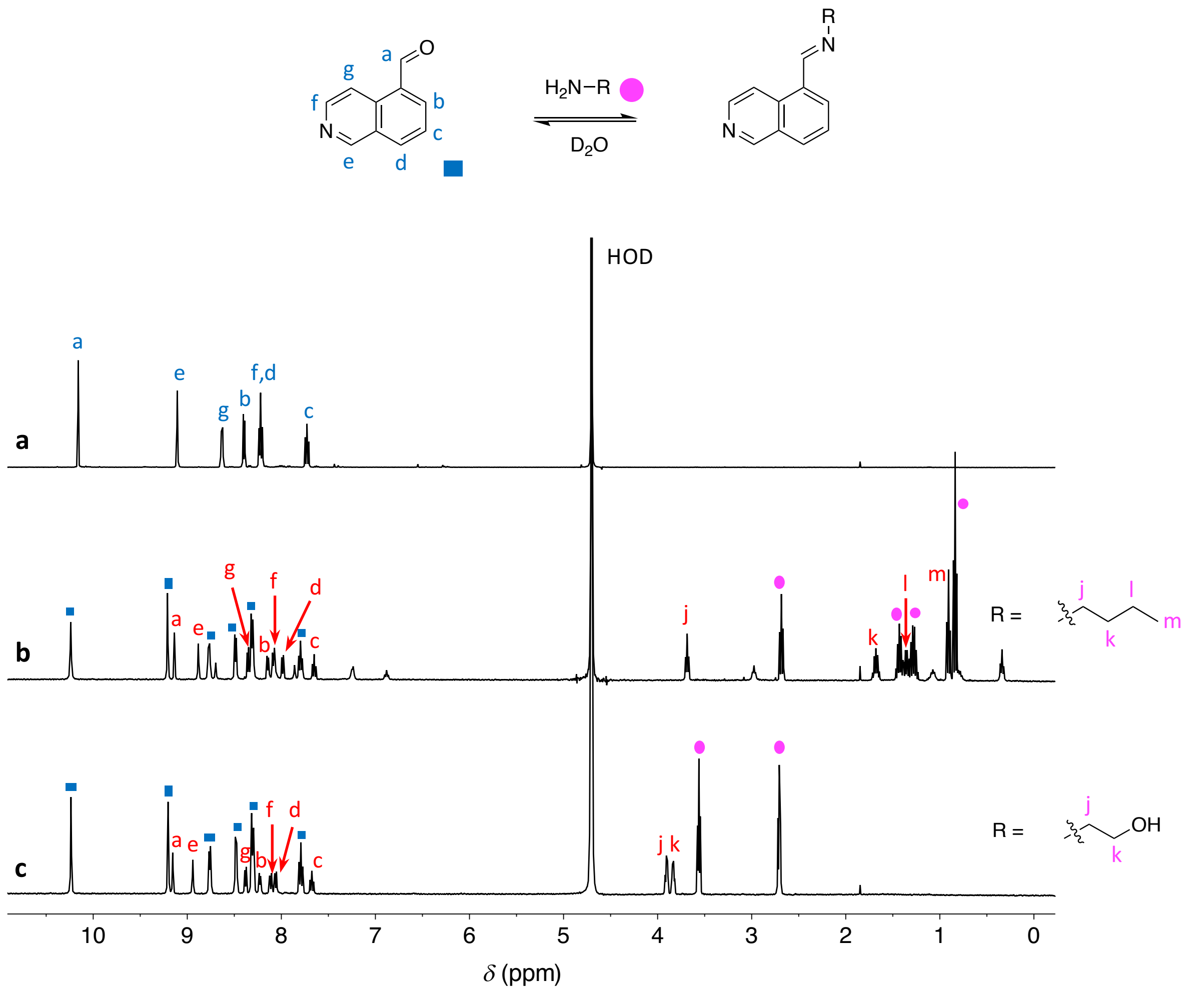

Figure S5. The presence of a permanent charge on the aromatic aldehyde favours the formation of the imine bond.
Comparison of the ^{1}H NMR spectra (D_2O, 400 MHz, 298 K) of isoquinoline-5-carboxaldehyde **(a)** alone, **(b)** after addition of butylamine, and **(c)** after addition of ethanolamine. The initial concentrations are 10 mM of aldehyde and 10 mM of amine. In spectra **(b)** and **(c)** only the protons of the imine products were assigned. Residual starting materials are labelled with blue squares (aldehyde) and pink circles (amine).

The amount of unreacted isoquinoline-5-carboxaldehyde left in spectra **(b)** and **(c)** is much higher (> 50%) than in the same experiment performed on **A1** (Fig. S4, < 50%), implying that the presence of the permanent charge favours imine formation.

3.2 Reversibility of the imine bond in water

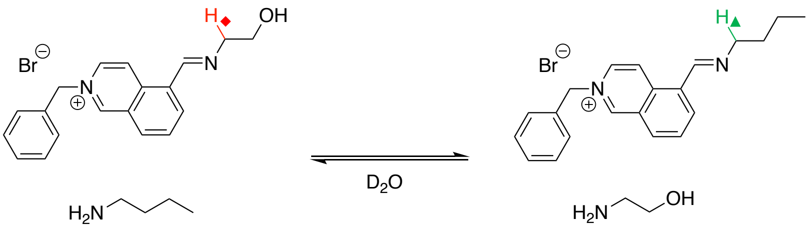

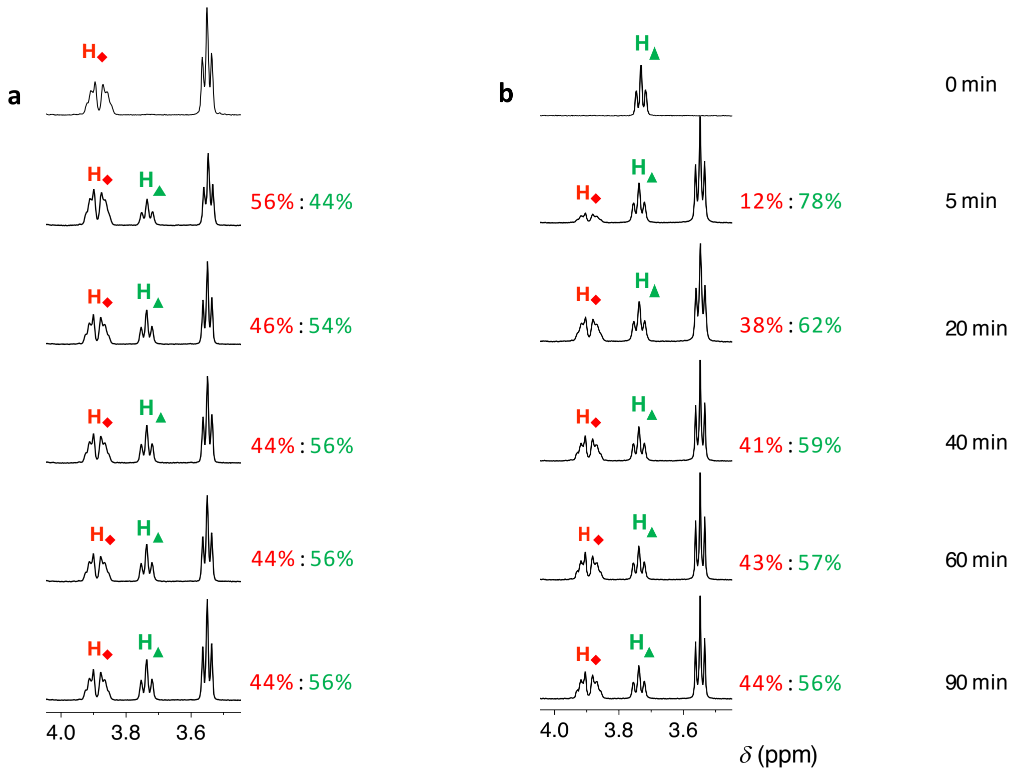

Figure S6. The imine bond is reversible in water and the composition of libraries is thermodynamically controlled.

(a) Partial ^{1}H NMR spectra (D_2O, 400 MHz, 298 K) showing imine exchange upon addition of butylamine to the pre-formed imine product of **A1** and ethanolamine (red diamond). **(b)** In a second experiment, imine exchange was followed after addition of ethanolamine to the pre-formed imine product of **A1** and butylamine (green triangle). The initial concentrations are 10 mM of aldehyde and 10 mM of amine. The kinetics of exchange are different, but the same distribution of products was obtained after 90 min, demonstrating that thermodynamic equilibrium is reached.

3.3 Influence of concentration, number of equivalents of diamine, pD and temperature on imine formation

The library composed of **A** and **B**$_7$ was used to determine the optimum conditions for [2]catenane formation. The effects of concentration, number of equivalents of diamine, pD and temperature were investigated.

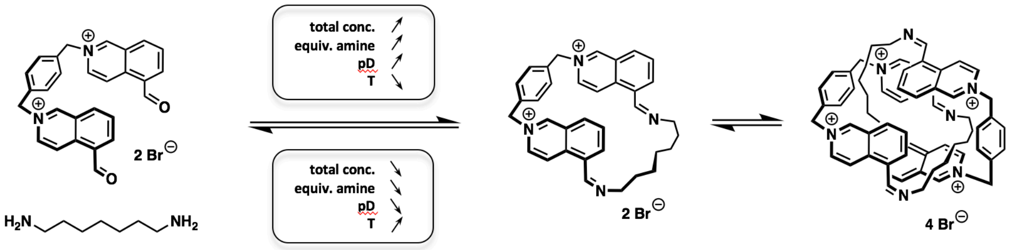

The [2]catenane **C**$_7$ forms more efficiently when using higher concentrations of building blocks, a higher number of equivalents of diamine, higher pD and lower temperatures.

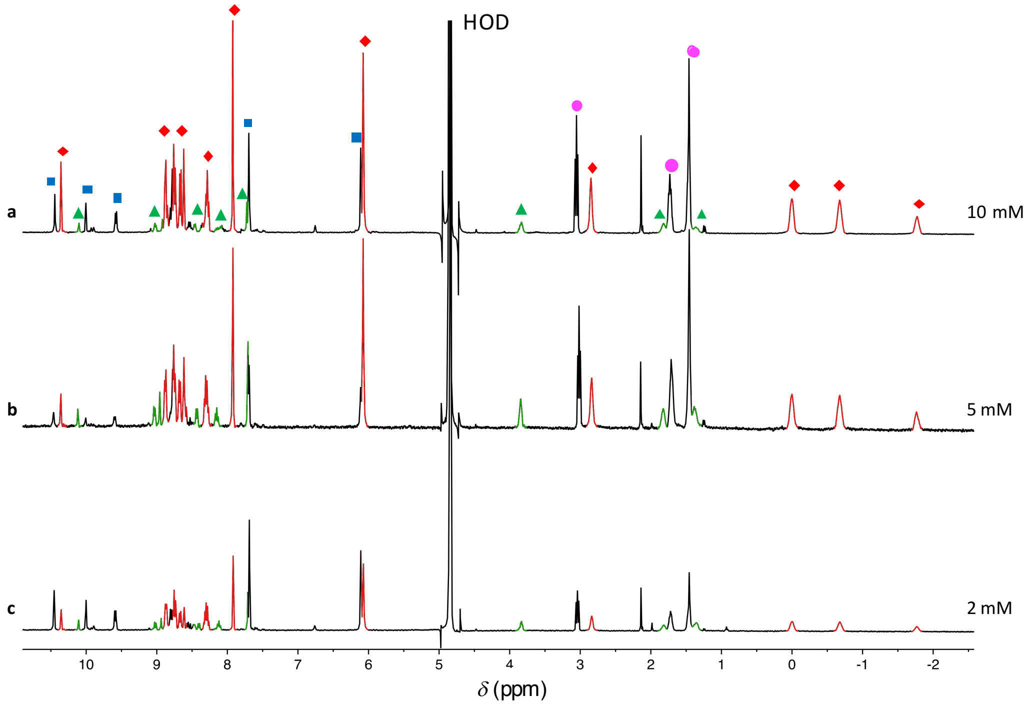

Figure S7. ^{1}H NMR spectra (D_2O, 400 MHz, 298 K) of the library composed of **A** and **B**$_7$ at various concentrations: **(a)** 10 mM, **(b)** 5 mM and **(c)** 2 mM of each building block. The concentration of 10 mM of each building block is the optimum concentration. Higher concentrations led to precipitation.

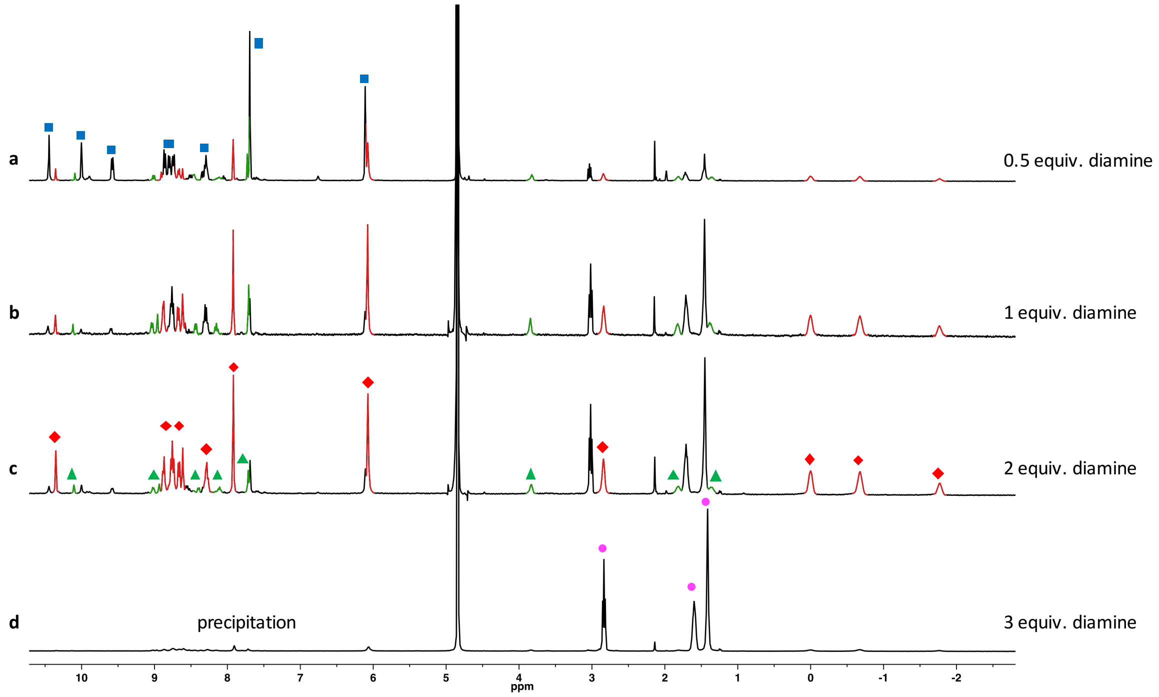

Figure S8. ^{1}H NMR spectra (D_2O, 400 MHz, 298 K) of the library composed of 10 mM of **A** and different number of equivalents of **B_7**: **(a)** 0.5, **(b)** 1, **(c)** 2 and **(d)** 3 equivalents of **B_7**. The higher numbers of equivalents of **B_7** favour the formation of the [2]catenane. Above 3 equivalents, a precipitate formed.

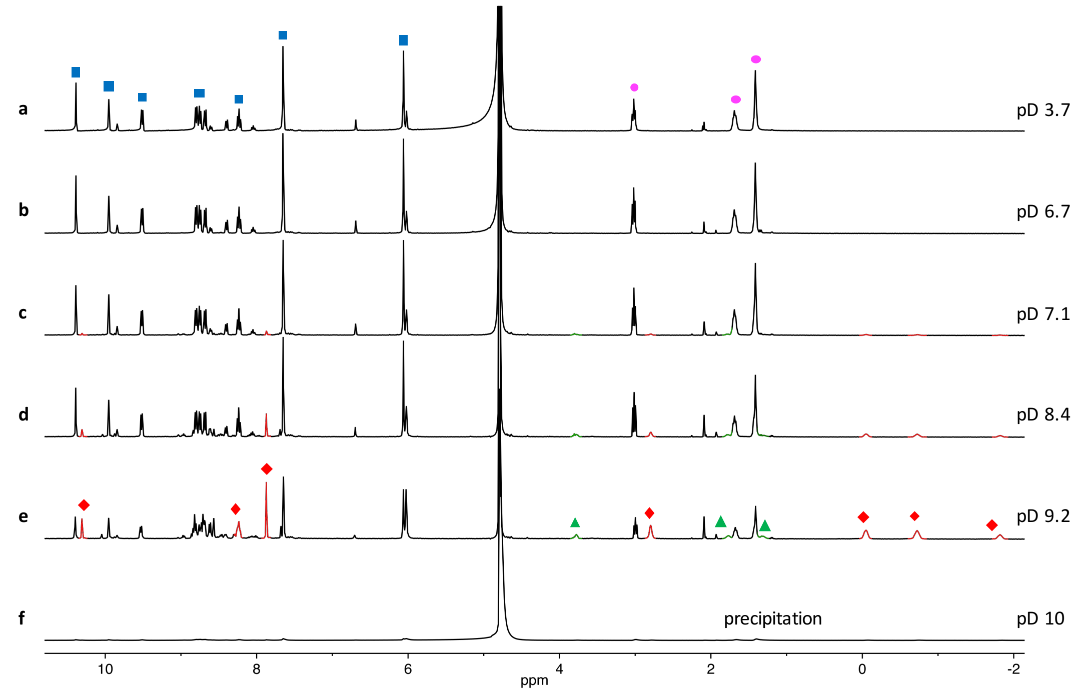

Figure S9. ^{1}H NMR spectra (D_2O, 400 MHz, 298 K) of the library composed of 10 mM of **A** and 10 mM of $\mathbf{B_7}$ at various pD: **(a)** pD = 3.7, **(b)** pD = 6.7, **(c)** pD = 7.1, **(d)** pD = 8.4, **(e)** pD = 9.2, **(f)** pD = 10. Above pD = 10, a precipitate formed.

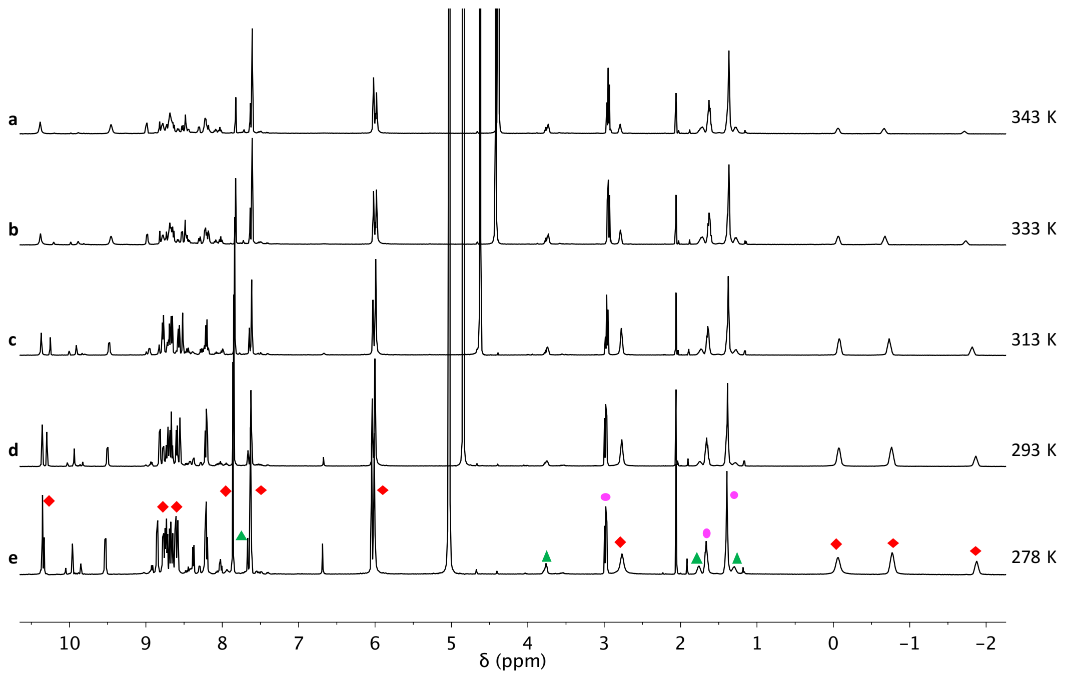

Figure S10. ^{1}H NMR spectra (D_2O, 400 MHz, 298 K) of the library composed of 10 mM of **A** and 10 mM of $\mathbf{B_7}$ at various temperatures: **(a)** T = 343 K, **(b)** T = 333 K, **(c)** T = 313 K, **(d)** T = 293 K, **(e)** T = 278 K.

4 Overall comparison of the libraries composed of A and B_n

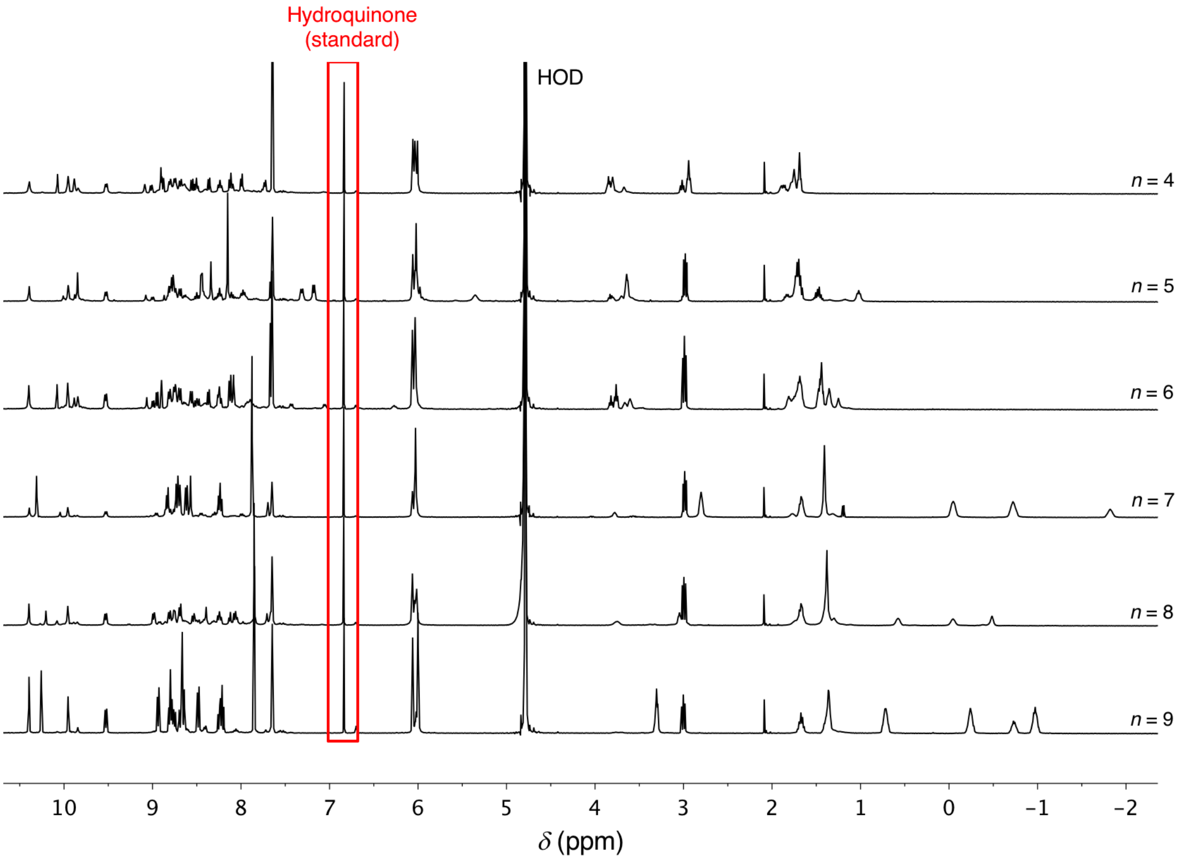

Figure S11. ^{1}H NMR spectra (D_2O, 400 MHz, 298 K) of the library composed of 10 mM of **A** and 10 mM of $\mathbf{B}_n$.

Table S1. Measured yields and conversions (%) corresponding to the values plotted in Fig. 1. The quantity of imine formed was measured from the integration of the NMR signal relative to an internal standard (10 mM of hydroquinone in D_2O) placed in a capillary immersed in the NMR tube containing the library. The values obtained represent an average of three independent experiments for each library. A yield of 0% indicates that the species could not be identified in the NMR spectrum. Hyphens indicate that the libraries were too complex to measure the yield of this particular species.

n	Yield of M_n (%)	Yield of C_n (%)	Total yield of imine (%)	Total conversion (%)
**4**	-	(0 ± 0)	(61.2 ± 3.9)	(61.0 ± 3.6)
**5**	-	(47.7 ± 4.2)	(73.4 ± 3.7)	(73.4 ± 3.8)
**6**	-	(0 ± 0)	(49.8 ± 4.7)	(50.2 ± 4.5)
**7**	(25.6 ± 4.5)	(44.0 ± 3.3)	(64.8 ± 3.8)	(67.3 ± 4.0)
**8**	(16.3 ± 4.7)	(18.6 ± 1.0)	(56.3 ± 4.3)	(62.8 ± 4.2)
**9**	(0 ± 0)	(45.5 ± 3.1)	(49.2 ± 3.7)	(69.3 ± 4.0)

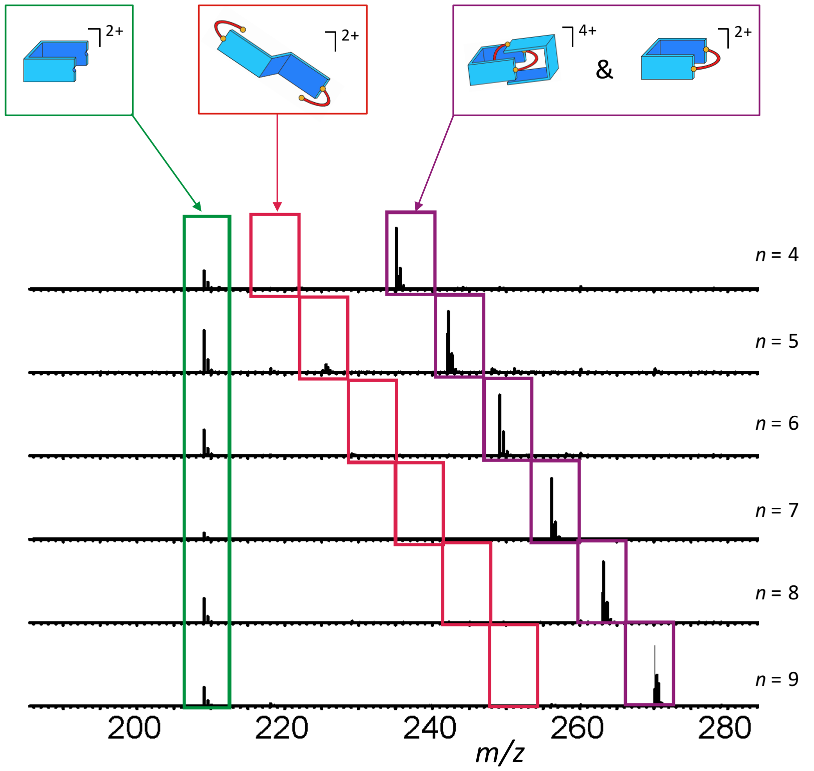

Figure S12. Comparison of the partial mass spectrum (*m/z* 200 to 300) for all libraries composed of 10 mM of **A** and 10 mM of $\mathbf{B}_n$.

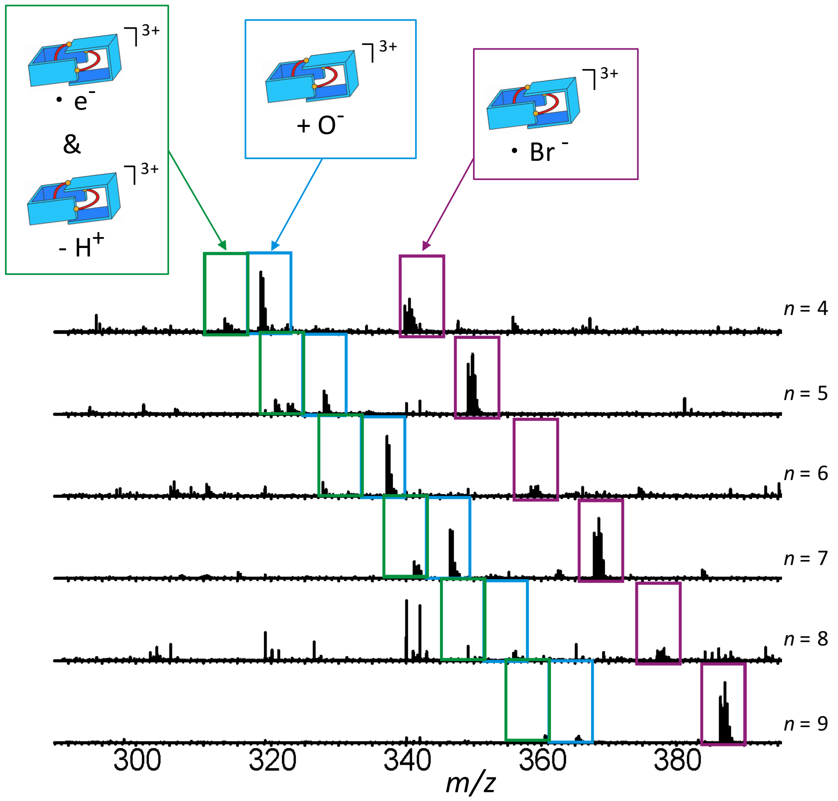

Figure S13. Comparison of the partial mass spectrum (*m/z* 300 to 400) for all libraries composed of 10 mM of **A** and 10 mM of $\mathbf{B}_n$.

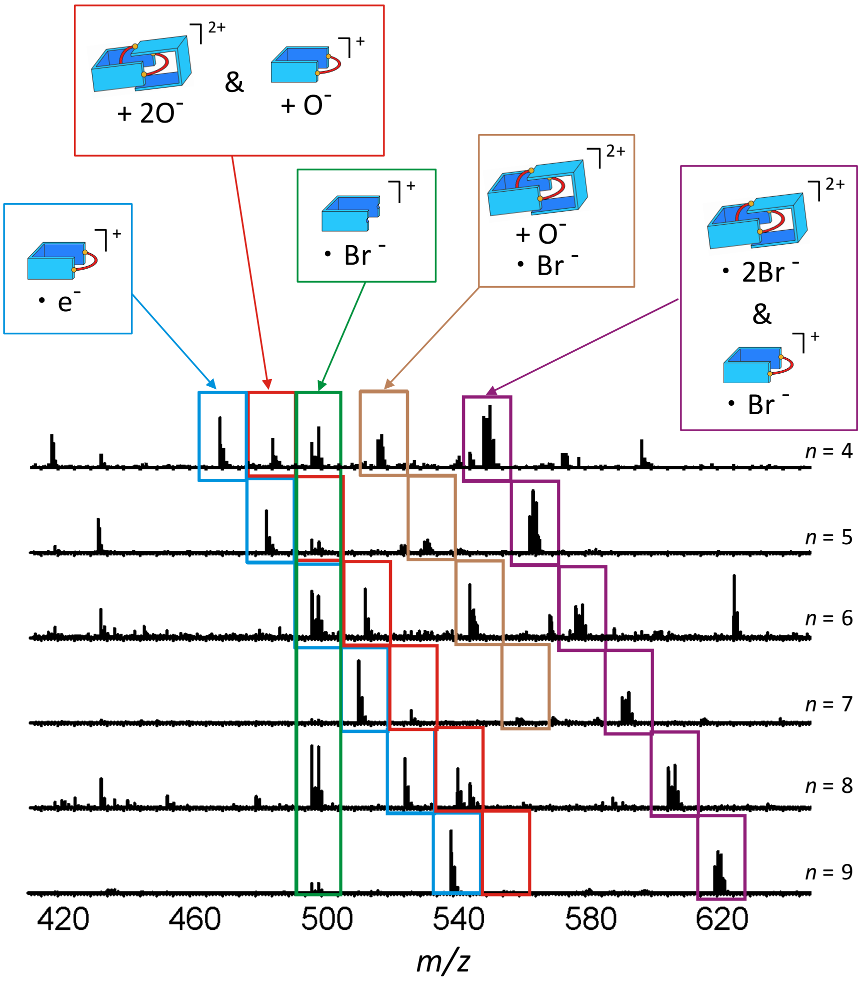

Figure S14. Comparison of the partial mass spectrum (*m/z* 400 to 650) for all libraries composed of 10 mM of **A** and 10 mM of $\mathbf{B}_n$. At higher *m/z* no significant peaks were detected.

5 Library composed of A and B_4

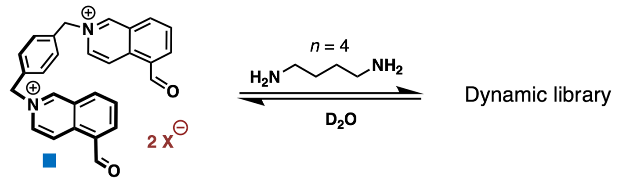

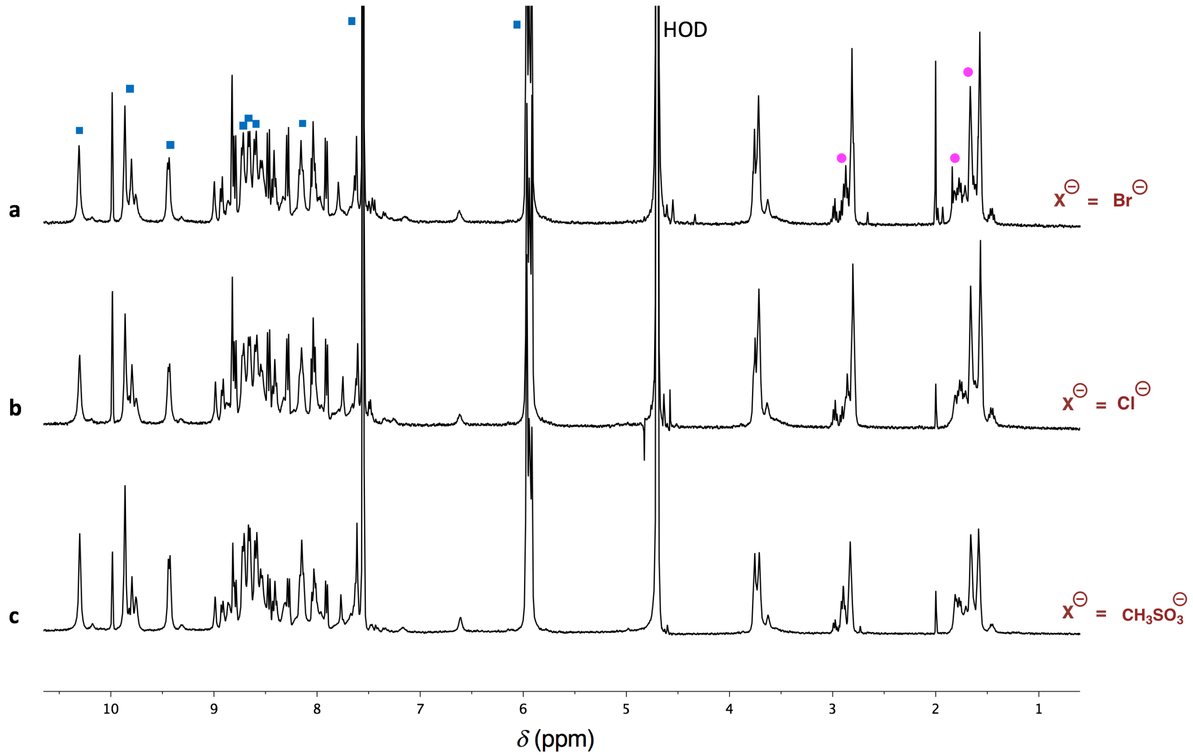

Figure S15. ^{1}H NMR spectra (D_2O, 400 MHz, 298 K) of the library composed of 5 mM of **A** and 5 mM of $\mathbf{B_4}$. The counter-ion is: **(a)** bromide, **(b)** chloride and **(c)** methanesulfonate.

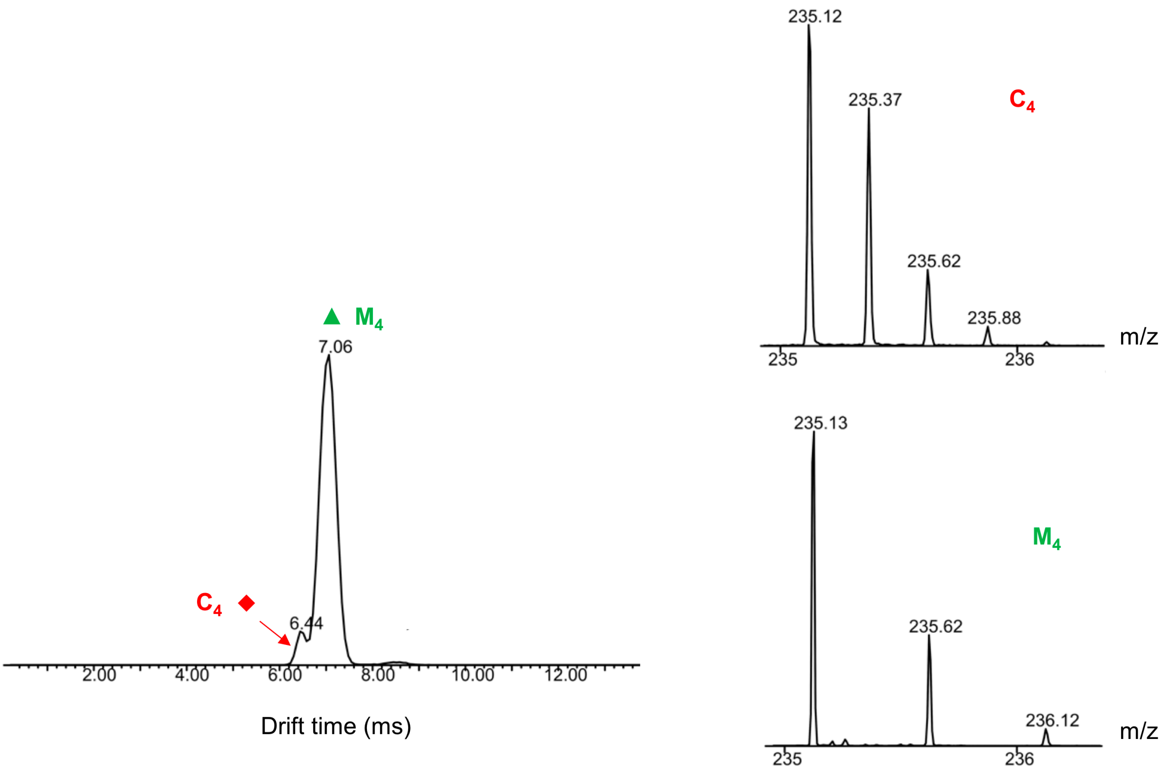

Figure S16. IMS separation of the *m/z* 235.12 peak corresponding to either [2]catenane $\mathbf{C_4}$ or macrocycle $\mathbf{M_4}$. The mass spectrum of each IMS peak is shown on the right side.

6 Library composed of A and B_5

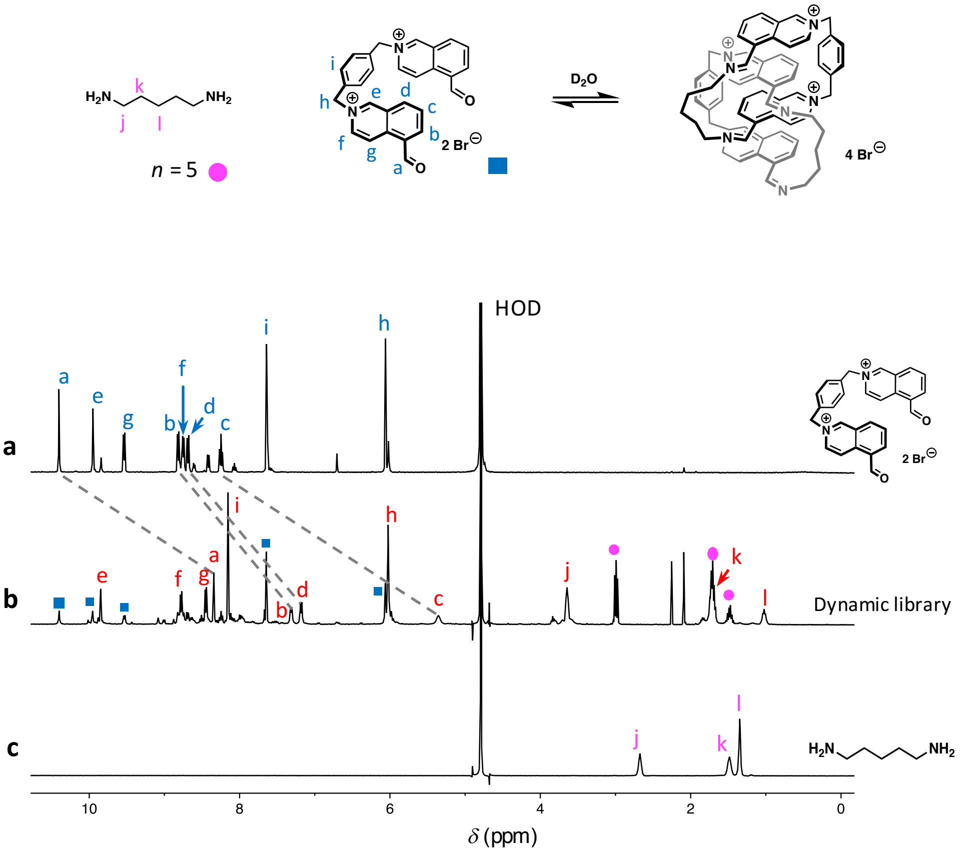

Figure S17. ^{1}H NMR spectra (D_2O, 400 MHz, 298 K) of the library composed of 10 mM of **A** and 10 mM of **B_5**. Comparison of the starting materials **(a)** **A** alone and **(c)** **B_5** alone with **(b)** the corresponding library. Only the signals corresponding to [2]catenane **C_5** were assigned. Residual starting materials are labelled with blue squares (**A**) and pink circles (**B_5**). The grey dotted lines highlight significant NMR shifts between the starting materials and **C_5**.

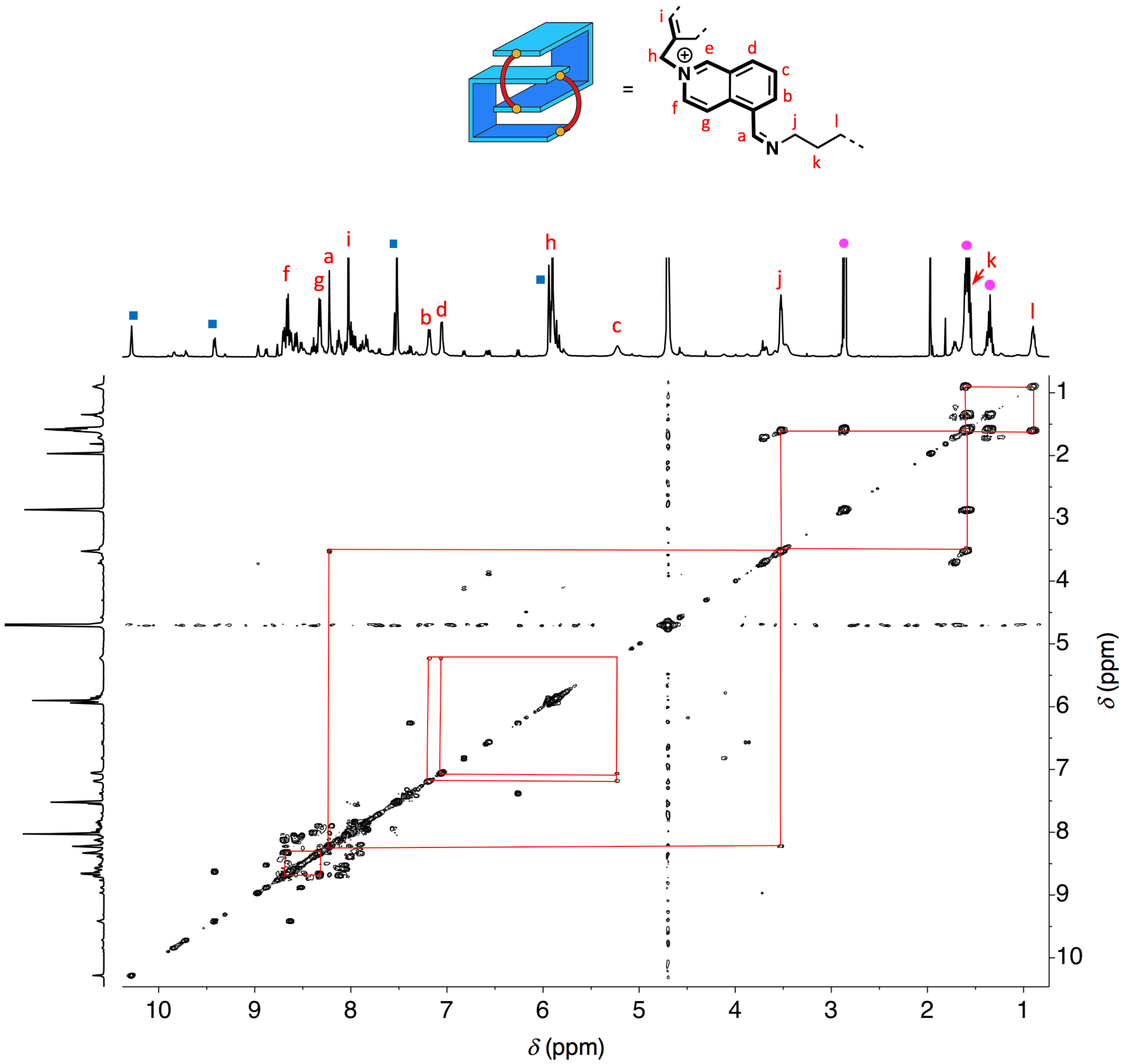

Figure S18. COSY (D_2O, 500 MHz, 298 K) of the library composed of 5 mM of **A** and 5 mM of $\mathbf{B_5}$. Correlations associated with $\mathbf{C_5}$ are highlighted in red.

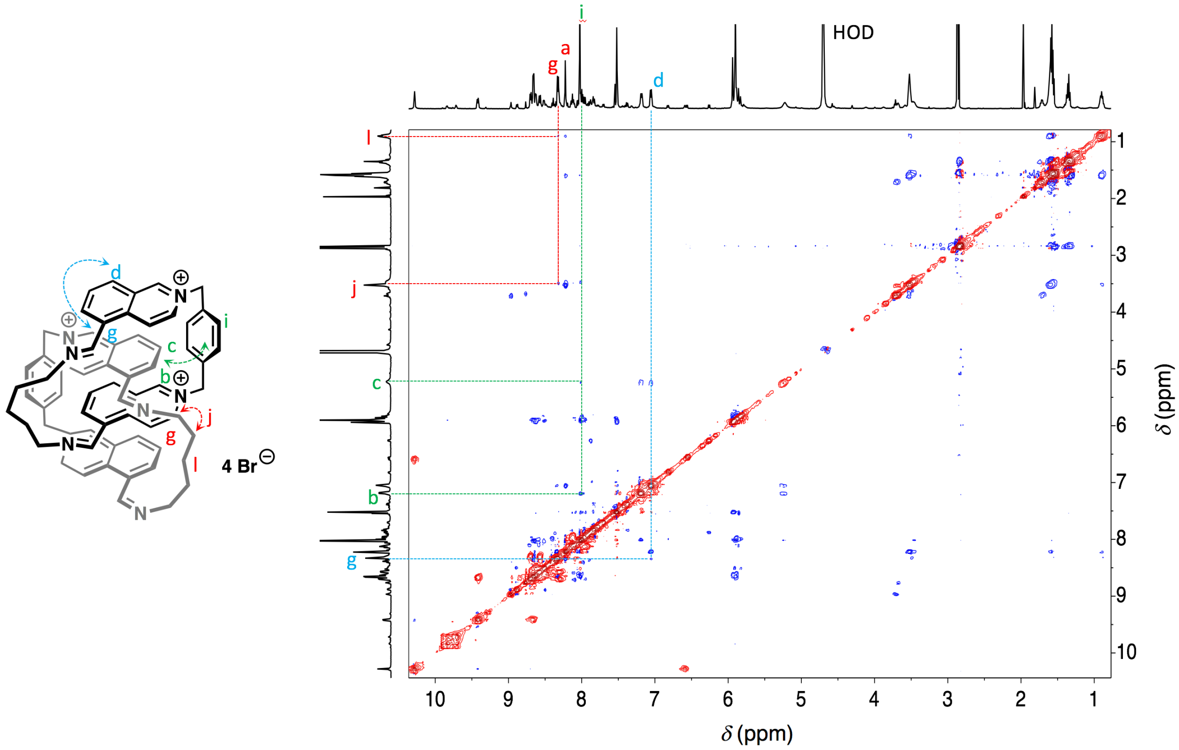

Figure S19. NOESY (D_2O, 500 MHz, 298 K, d_8 = 500 ms) of the library composed of 5 mM of **A** and 5 mM of $\mathbf{B_5}$. Correlations in agreement with the proposed structure of $\mathbf{C_5}$ are highlighted with dotted lines.

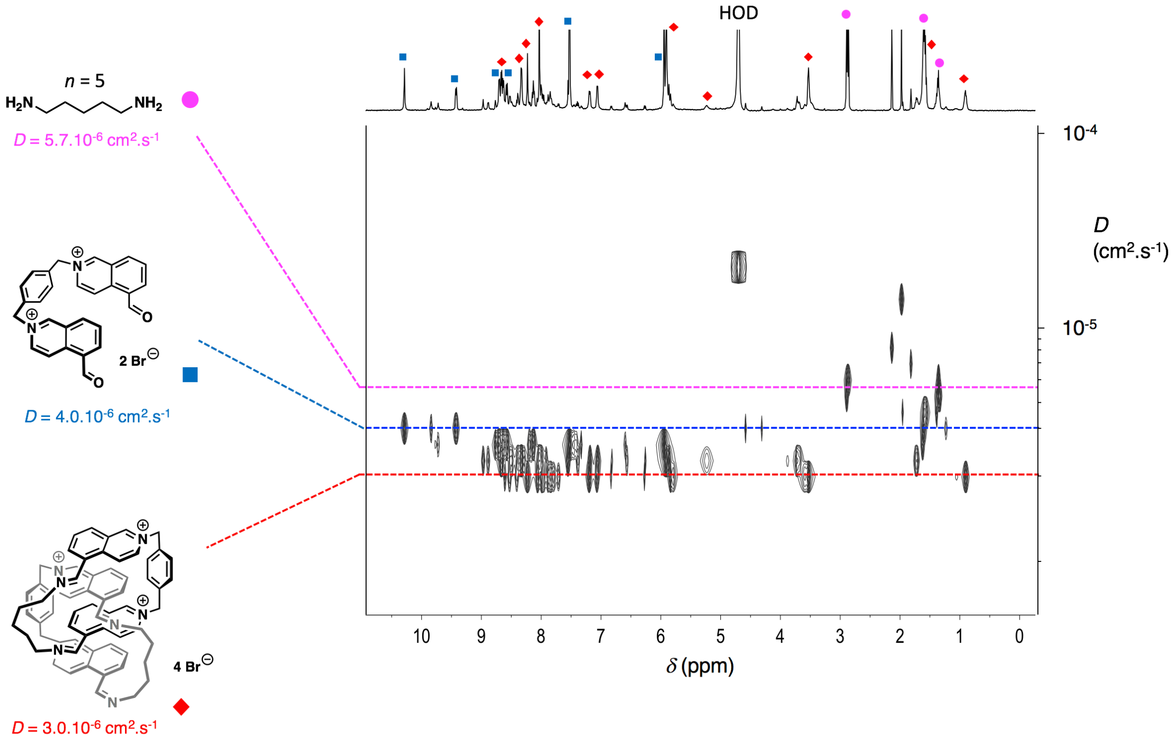

Figure S20. DOSY (D_2O, 500 MHz, 298 K) of the library composed of 5 mM of **A** and 5 mM of **B$_5$**. Each species of the library is highlighted with a dotted line. The diffusion coefficients were measured for **B$_5$** ($5.7.10^{-6}$ $cm^2 \cdot s^{-1}$), **A** ($4.0.10^{-6}$ $cm^2 \cdot s^{-1}$) and **C$_5$** ($3.0.10^{-6}$ $cm^2 \cdot s^{-1}$). Assuming the molecule to be spherical, these values of diffusion coefficients correspond to volumes of $0.12.10^{-27}$, $0.35.10^{-27}$ and $0.83.10^{-27}$ m^3, respectively. Volumes were calculated using the Stokes-Einstein equation at 298 K ($k = 1.381.10^{-23}$ $m^2 \cdot kg \cdot s^{-2} \cdot K^{-1}$, η_{D2O} = 1.25 $x10^{-3}$ $kg \cdot m^{-1} \cdot s^{-1}$).

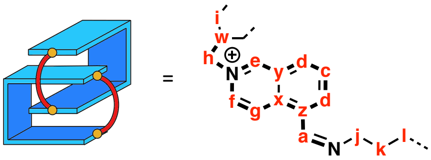

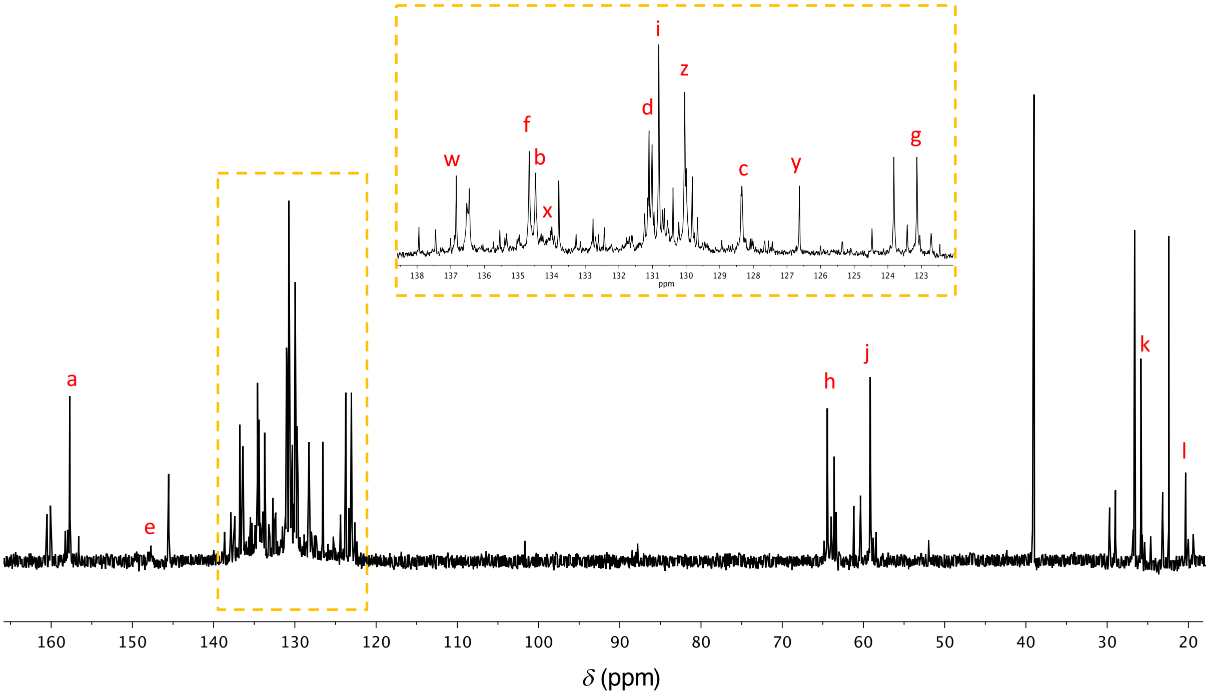

Figure S21. ^{13}C NMR (D_2O, 125 MHz, 298 K) of the library composed of 5 mM of **A** and 5 mM of **B$_5$**. Only the signals corresponding to [2]catenane **C$_5$** were assigned. The red insert shows an expansion of the aromatic region.

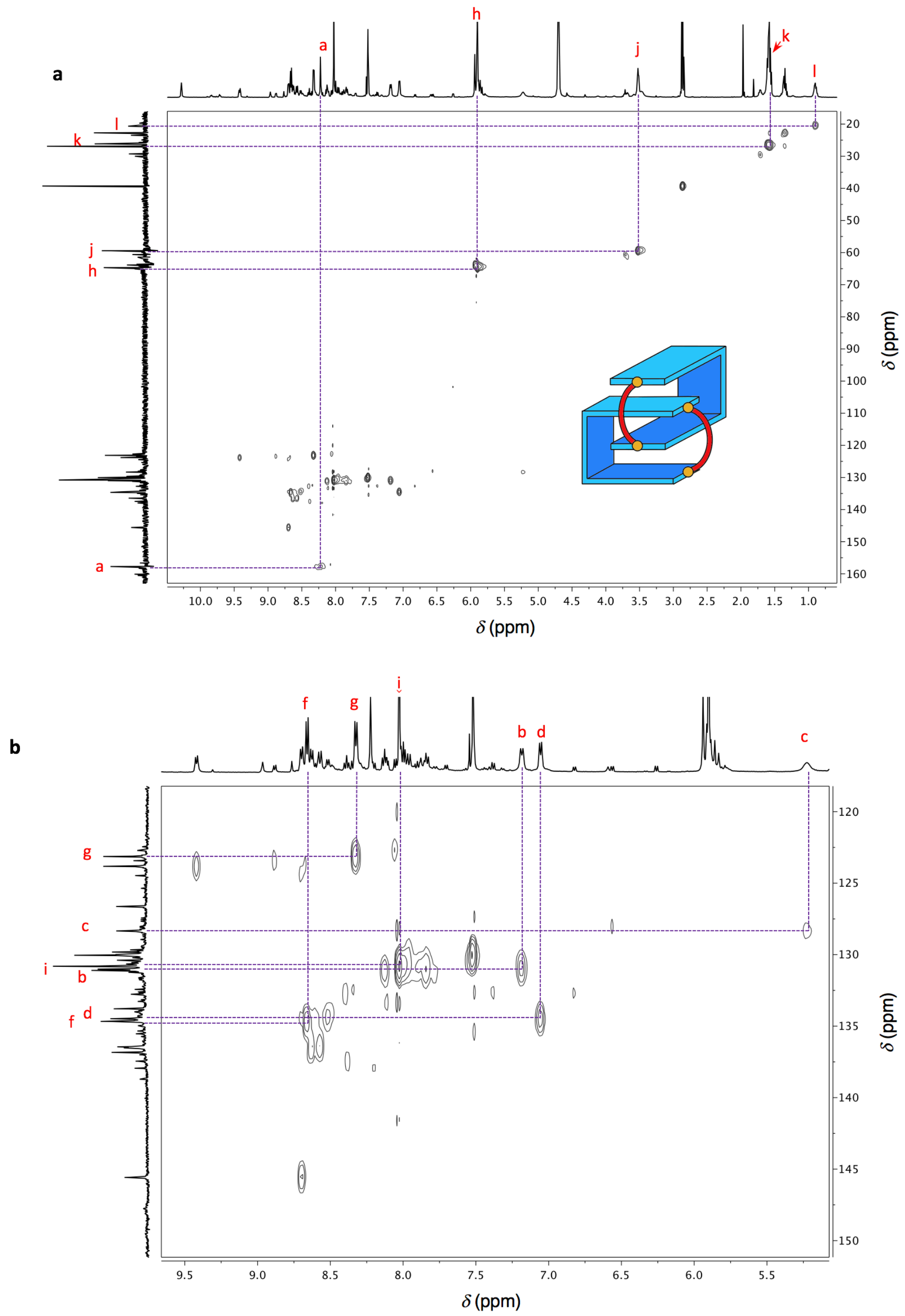

Figure S22. HSQC (D_2O, 125 MHz, 298 K) of the library composed of 5 mM of **A** and 5 mM of $\mathbf{B_5}$. **(a)** Full spectrum and **(b)** expansion of the aromatic region. Correlations between protons and carbons corresponding to [2]catenane $\mathbf{C_5}$ are highlighted with purple dotted lines.

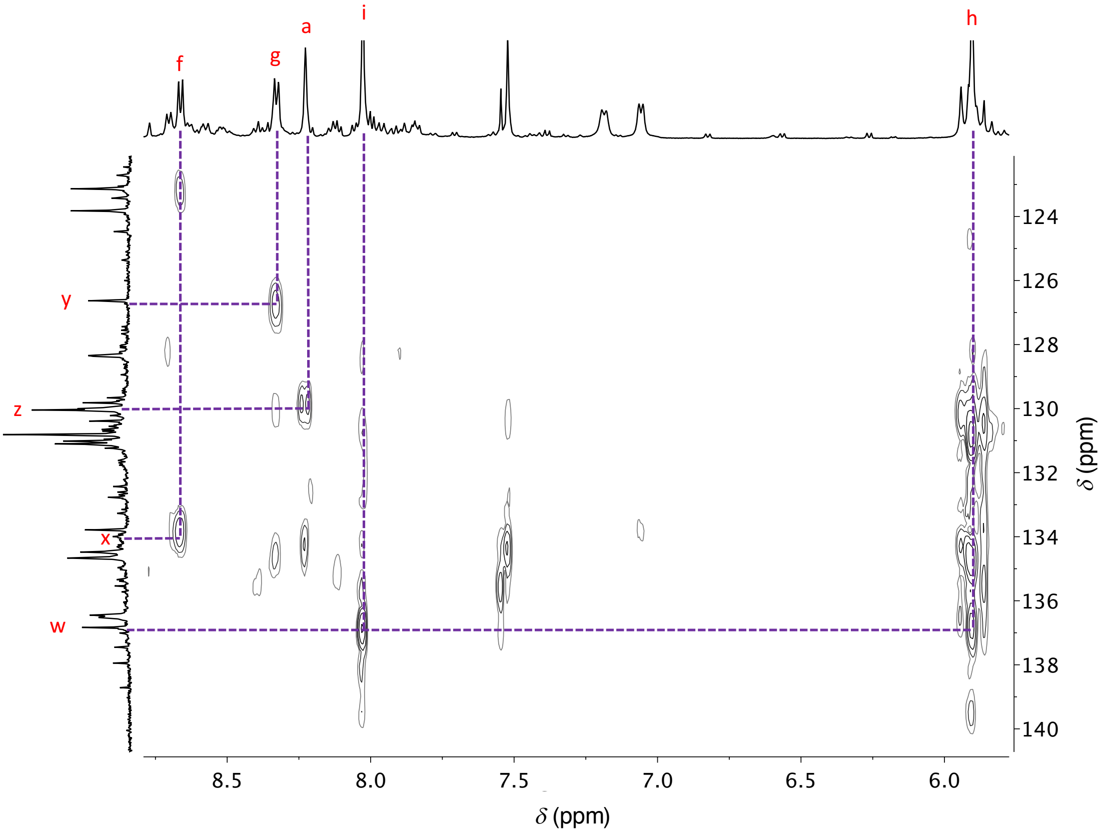

Figure S23. HMBC (D_2O, 125 MHz, 298 K) of the library composed of 5 mM of **A** and 5 mM of $\mathbf{B_5}$. Expansion of the aromatic region. Correlations between protons and carbons corresponding to [2]catenane $\mathbf{C_5}$ are highlighted with purple doted lines.

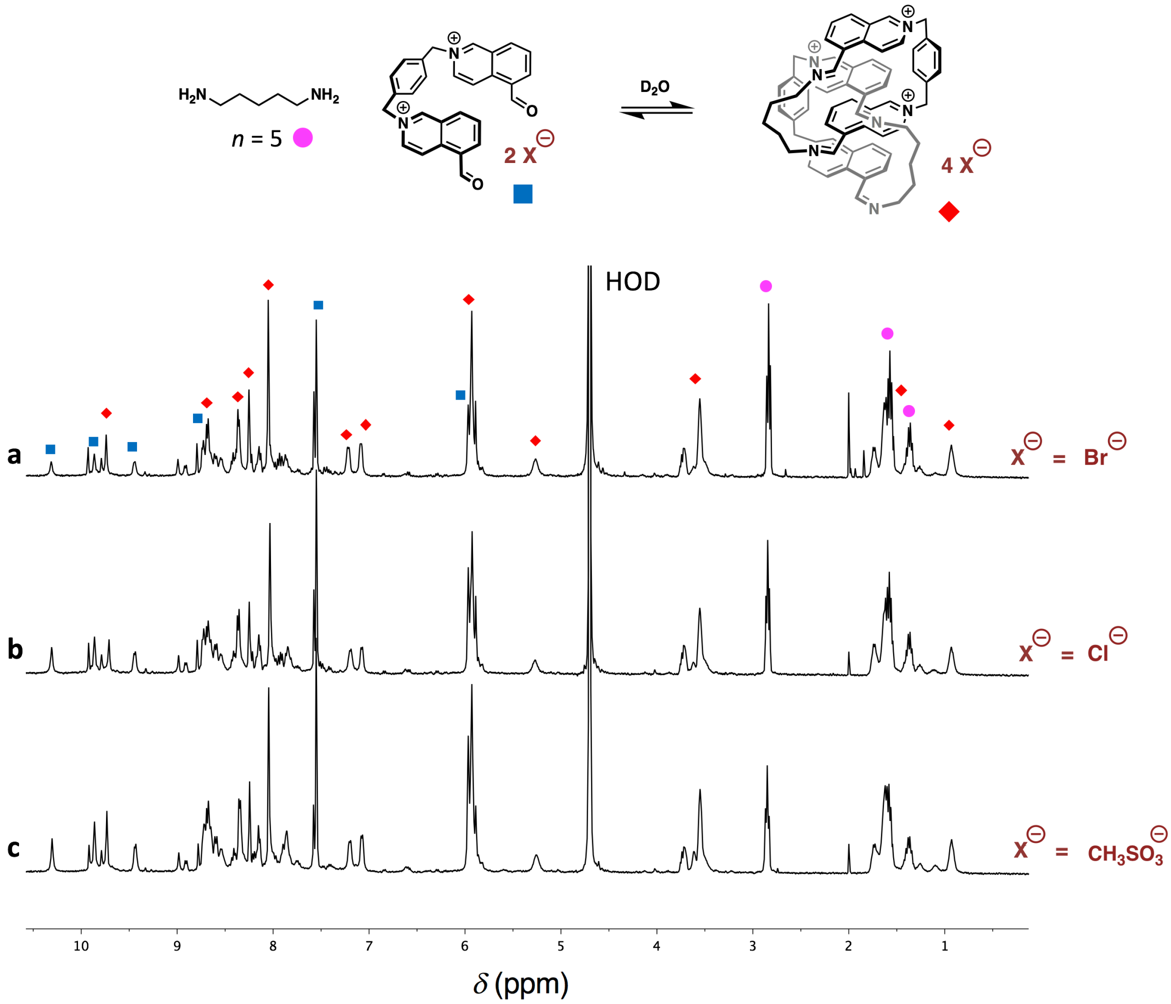

Figure S24. ^{1}H NMR spectra (D_2O, 400 MHz, 298 K) of the library composed of 5 mM of **A** and 5 mM of $\mathbf{B_5}$. The counter-ion is: (a) bromide, (b) chloride and (c) methanesulfonate.

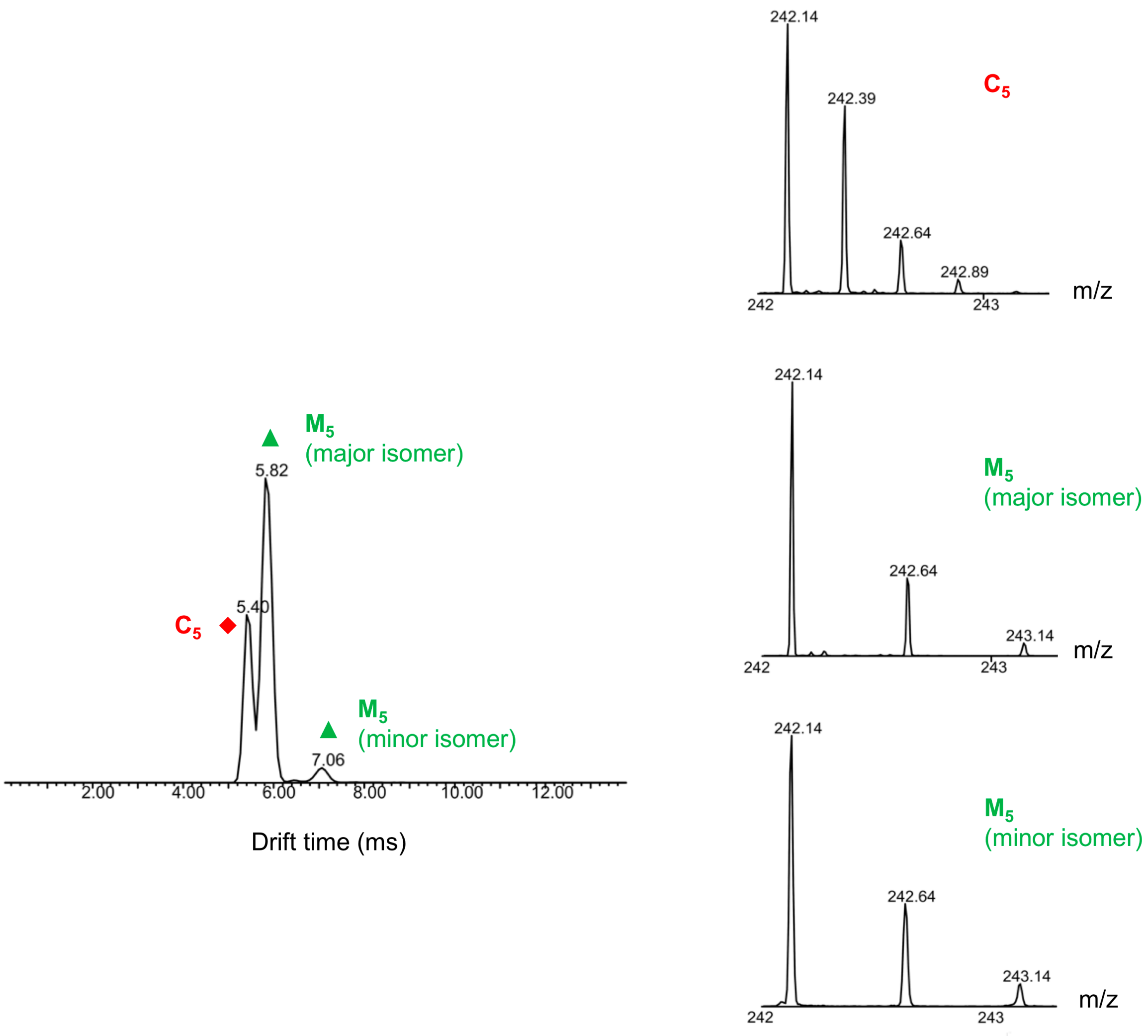

Figure S25. IMS separation of the *m/z* 242.14 peak corresponding to either [2]catenane $\mathbf{C_5}$ or macrocycle $\mathbf{M_5}$. The mass spectrum of each IMS peak is shown on the right side. The minor isomer of $\mathbf{M_5}$ probably arises from the insource fragmentation of the [2]catenane and corresponds to the open macrocycle.

7 Library composed of A and B_6

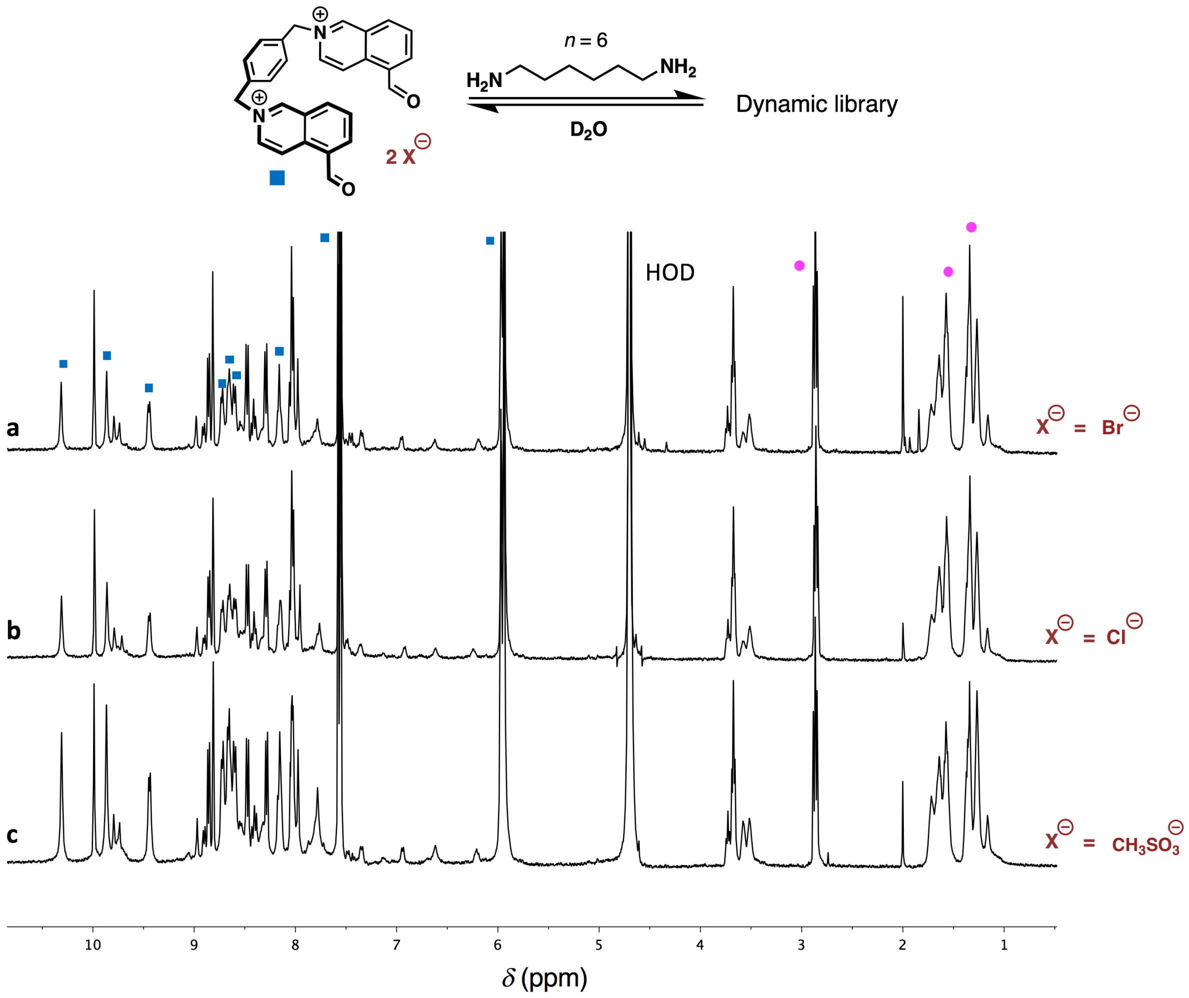

Figure S26. ^{1}H NMR spectra (D_2O, 400 MHz, 298 K) of the library composed of 5 mM of **A** and 5 mM of $\mathbf{B_6}$. The counter-ion is: **(a)** bromide, **(b)** chloride and **(c)** methanesulfonate.

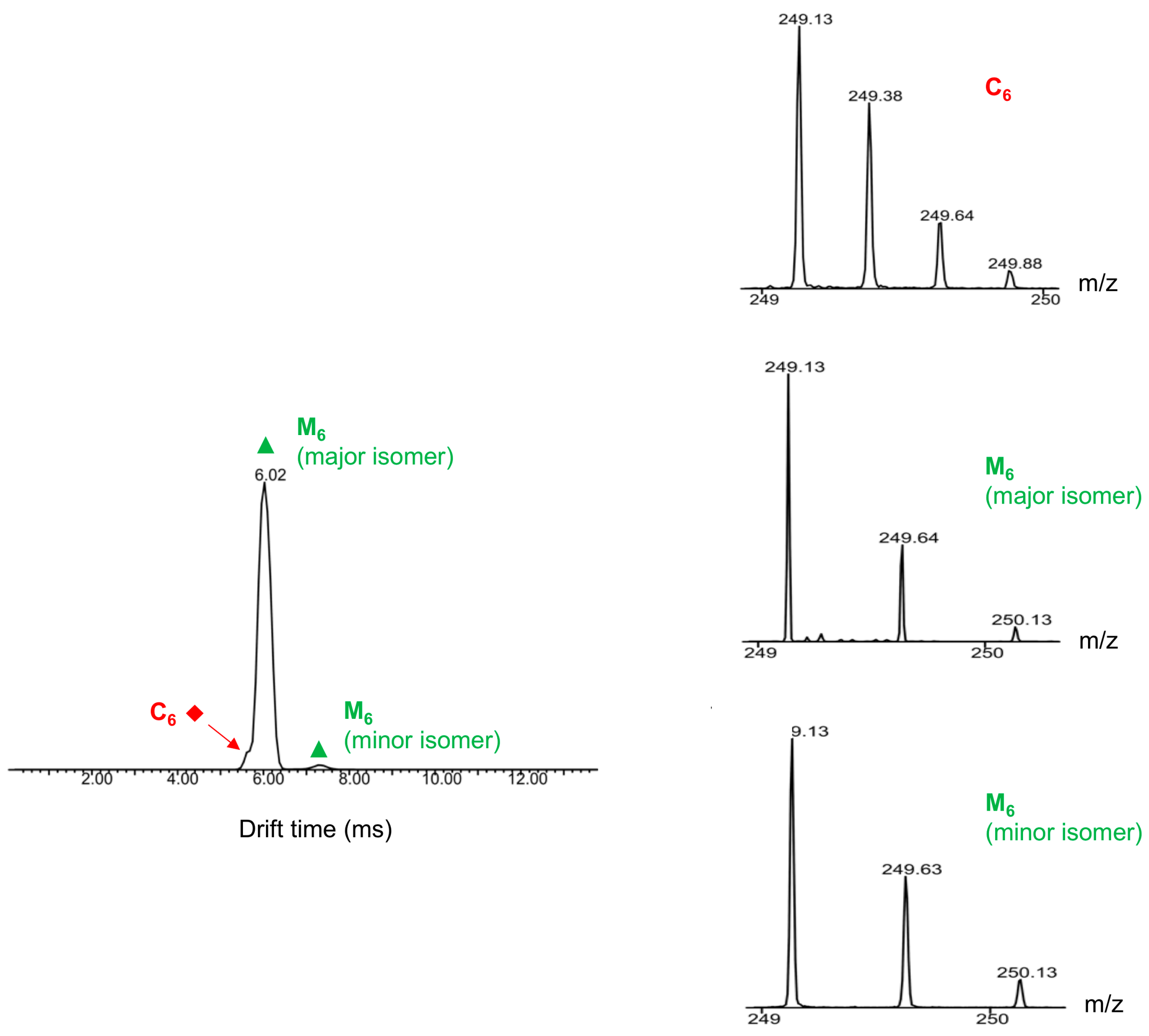

Figure S27. IMS separation of the *m/z* 249.13 peak corresponding to either [2]catenane $\mathbf{C_6}$ or macrocycle $\mathbf{M_6}$. The mass spectrum of each IMS peak is shown on the right side. The minor isomer of $\mathbf{M_6}$ probably arises from the insource fragmentation of the [2]catenane and corresponds to the open macrocycle.

8 Library composed of A^{2+} and B_7

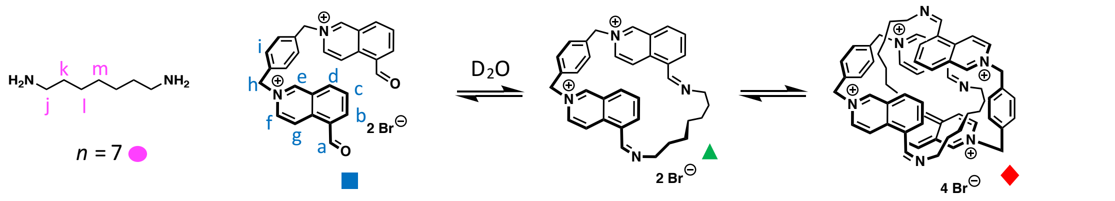

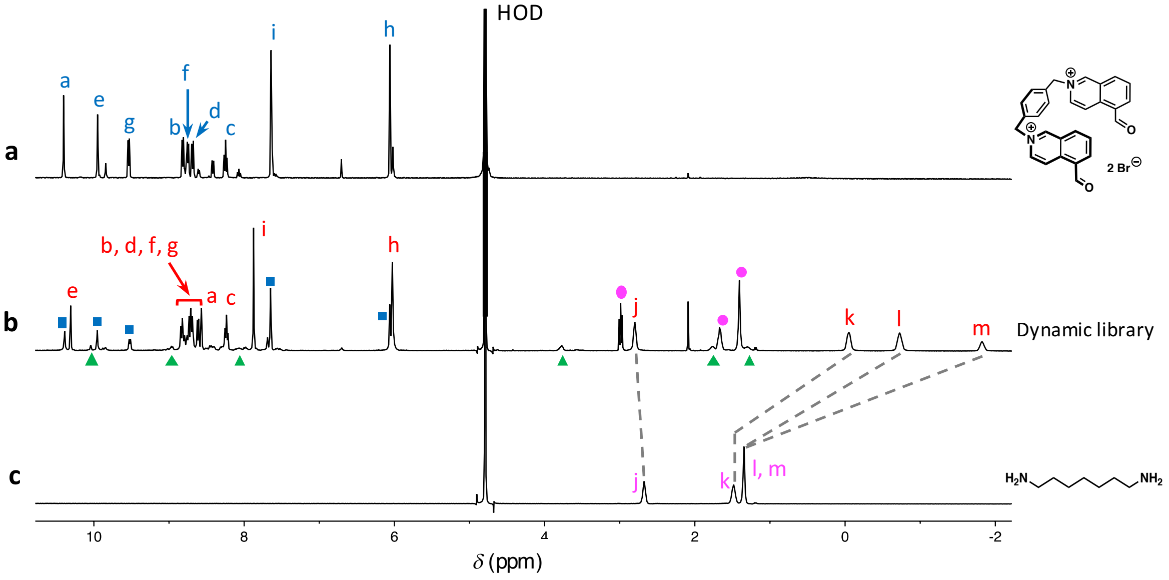

Figure S28. ^{1}H NMR spectra (D_2O, 400 MHz, 298 K) of the library composed of 10 mM of **A** and 10 mM of $\mathbf{B_7}$. Comparison of the starting materials **(a)** **A** alone and **(c)** $\mathbf{B_7}$ alone with **(b)** the corresponding library. Only the signals corresponding to [2]catenane $\mathbf{C_7}$ were assigned. Residual starting materials are labelled with blue squares (**A**) and pink circles ($\mathbf{B_7}$). The grey dotted lines highlight significant NMR shifts between the starting materials and $\mathbf{C_7}$.

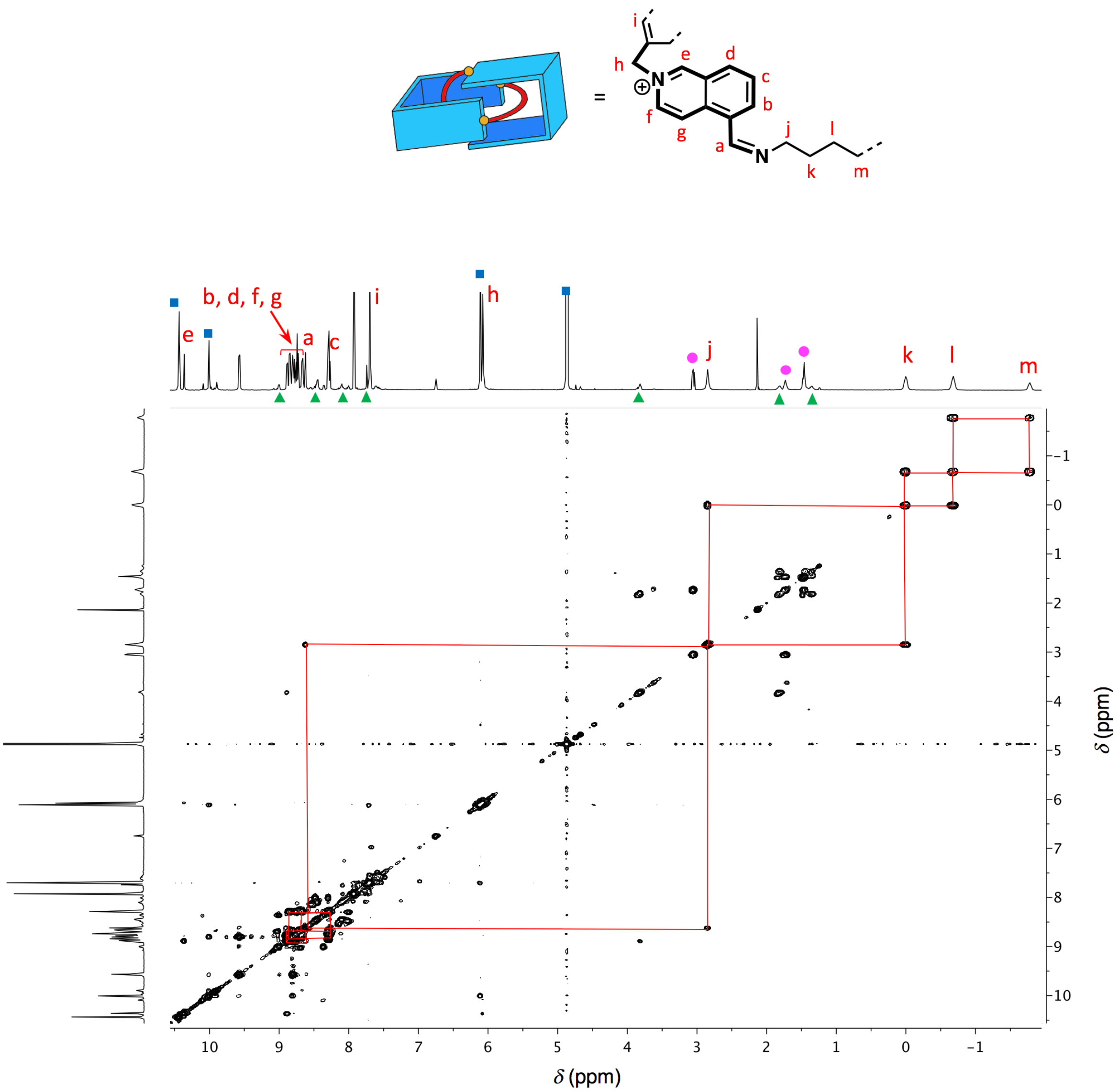

Figure S29. COSY (D_2O, 500 MHz, 298 K) of the library composed of 5 mM of **A** and 5 mM of $\mathbf{B_7}$. Correlations associated with $\mathbf{C_7}$ are highlighted in red.

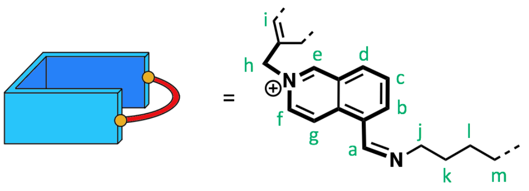

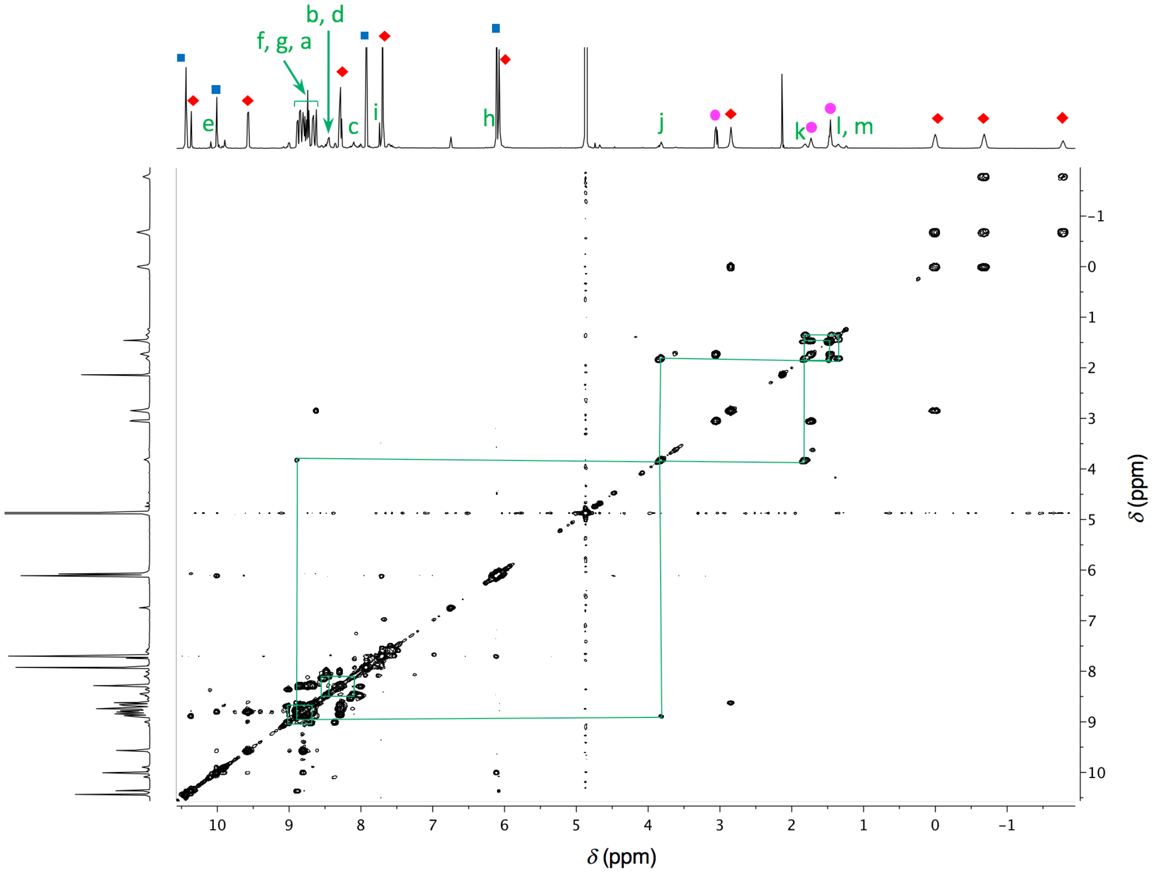

Figure S30. COSY (D_2O, 500 MHz, 298 K) of the library composed of 5 mM of **A** and 5 mM of **B$_7$**. Correlations associated with **M$_7$** are highlighted in green.

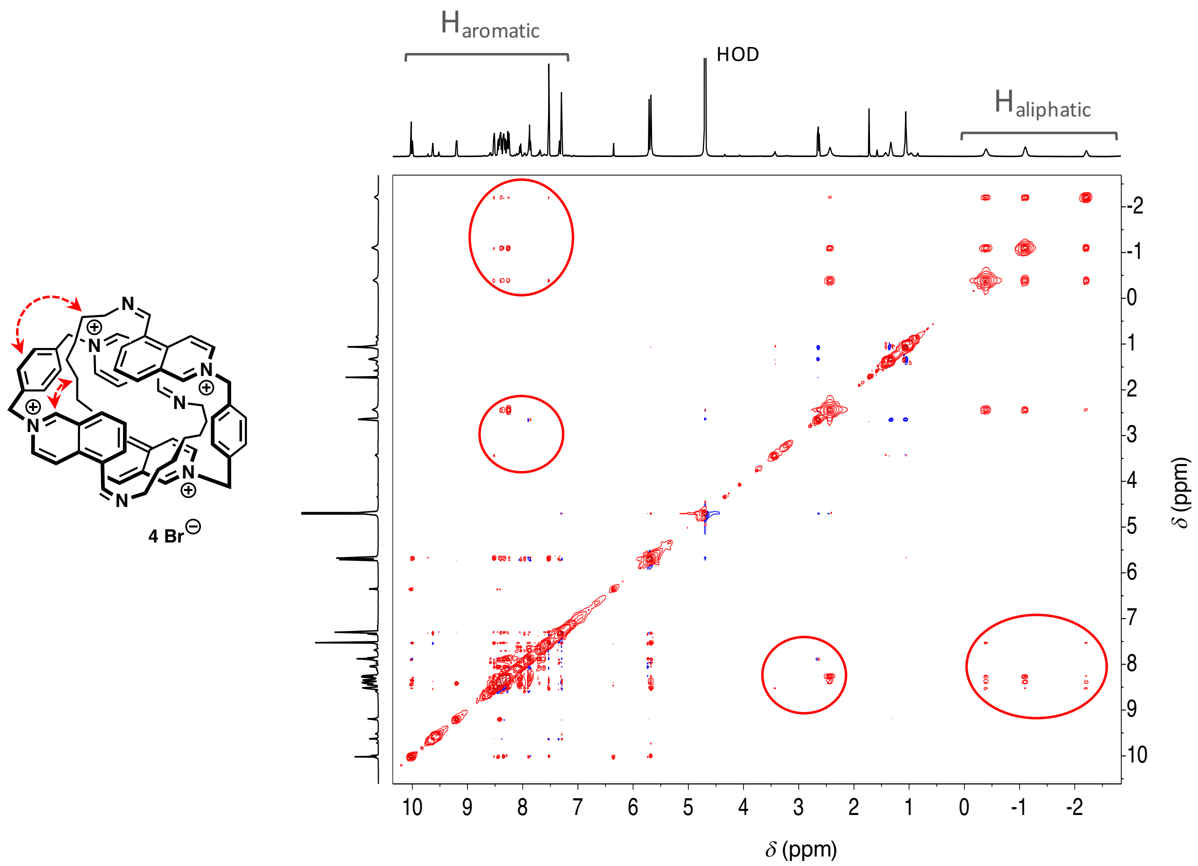

Figure S31. NOESY (D_2O, 500 MHz, 298 K, d_8 = 300 ms) of the library composed of 5 mM of **A** and 5 mM of $\mathbf{B_7}$. Correlations in agreement with the proposed structure of $\mathbf{C_7}$ are highlighted with dotted lines.

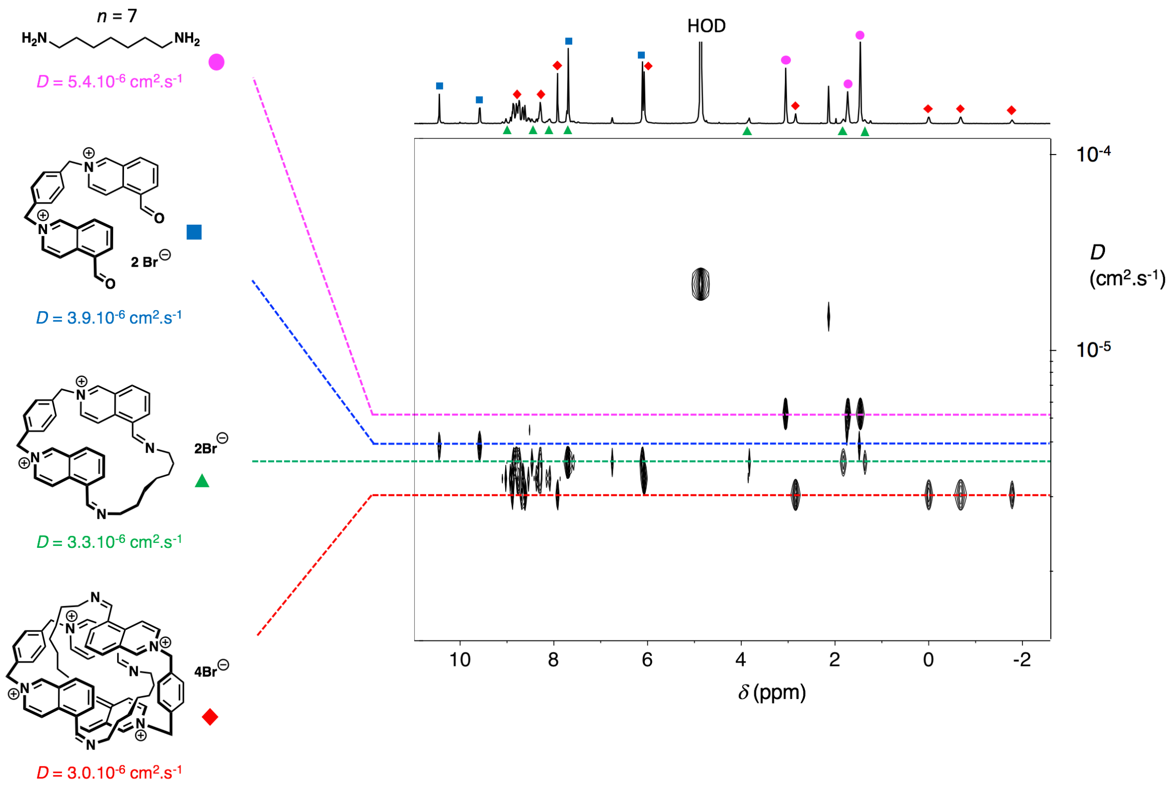

Figure S32. DOSY (D_2O, 500 MHz, 298 K) of the library composed of 5 mM of **A** and 5 mM of **B$_7$**. Each species of the library is highlighted with a dotted line. The diffusion coefficients were measured for **B$_7$** ($5.4.10^{-6}$ $cm^2{\cdot}s^{-1}$), **A** ($3.9.10^{-6}$ $cm^2{\cdot}s^{-1}$), **M$_7$** ($3.3.10^{-6}$ $cm^2{\cdot}s^{-1}$) and **C$_7$** ($3.0.10^{-6}$ $cm^2{\cdot}s^{-1}$). Assuming the molecule to be spherical, these values of diffusion coefficients correspond to volumes of $0.14.10^{-27}$, $0.36.10^{-27}$ and $0.83.10^{-27}$ m^3, respectively. Volumes were calculated using the Stokes-Einstein equation at 298 K (k = $1.381.10^{-23}$ $m^2{\cdot}kg{\cdot}s^{-2}{\cdot}K^{-1}$, $\eta_{D2O} = 1.25.10^{-3}$ $kg{\cdot}m^{-1}{\cdot}s^{-1}$).

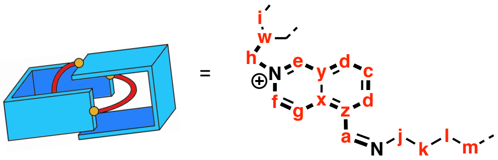

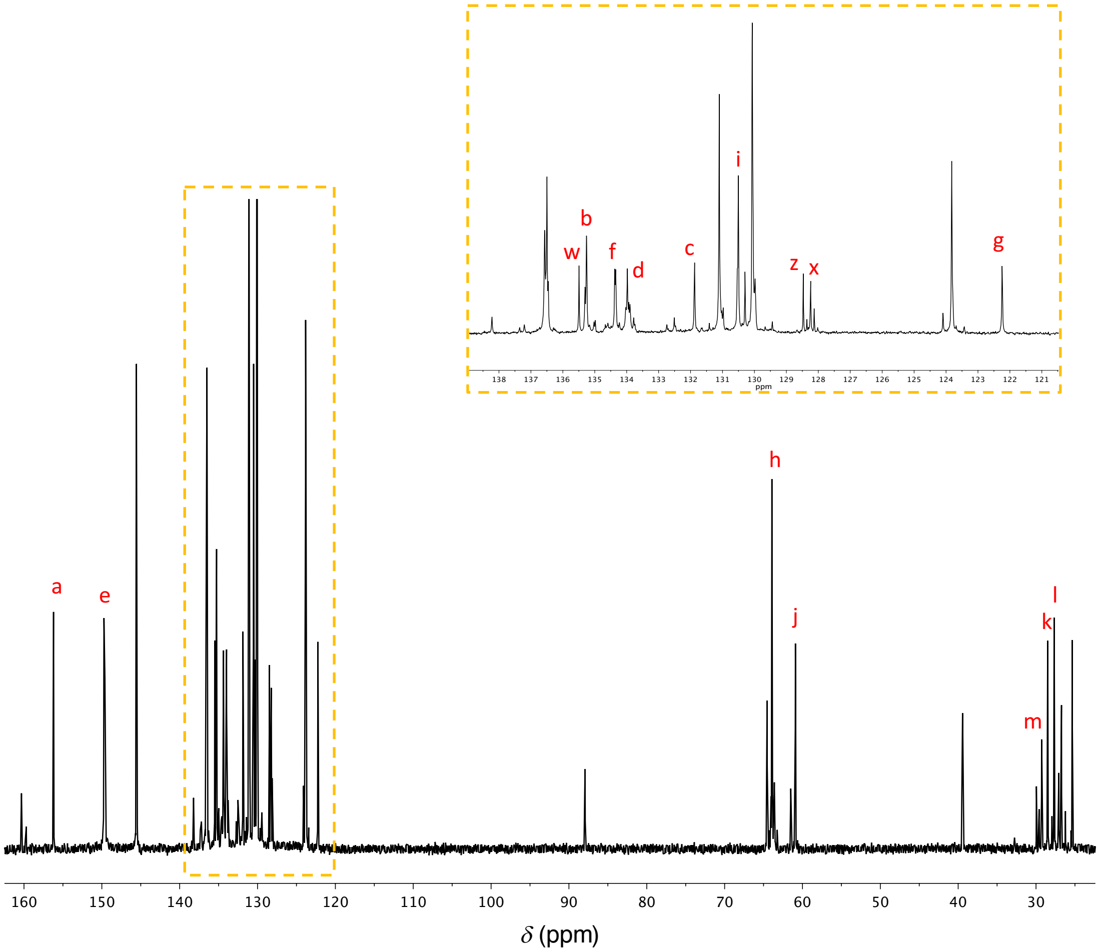

Figure S33. ^{13}C NMR (D_2O, 125 MHz, 298 K) of the library composed of 5 mM of **A** and 5 mM of **B**$_7$. Only the signals corresponding to [2]catenane **C**$_7$ were assigned. The red insert shows an expansion of the aromatic region.

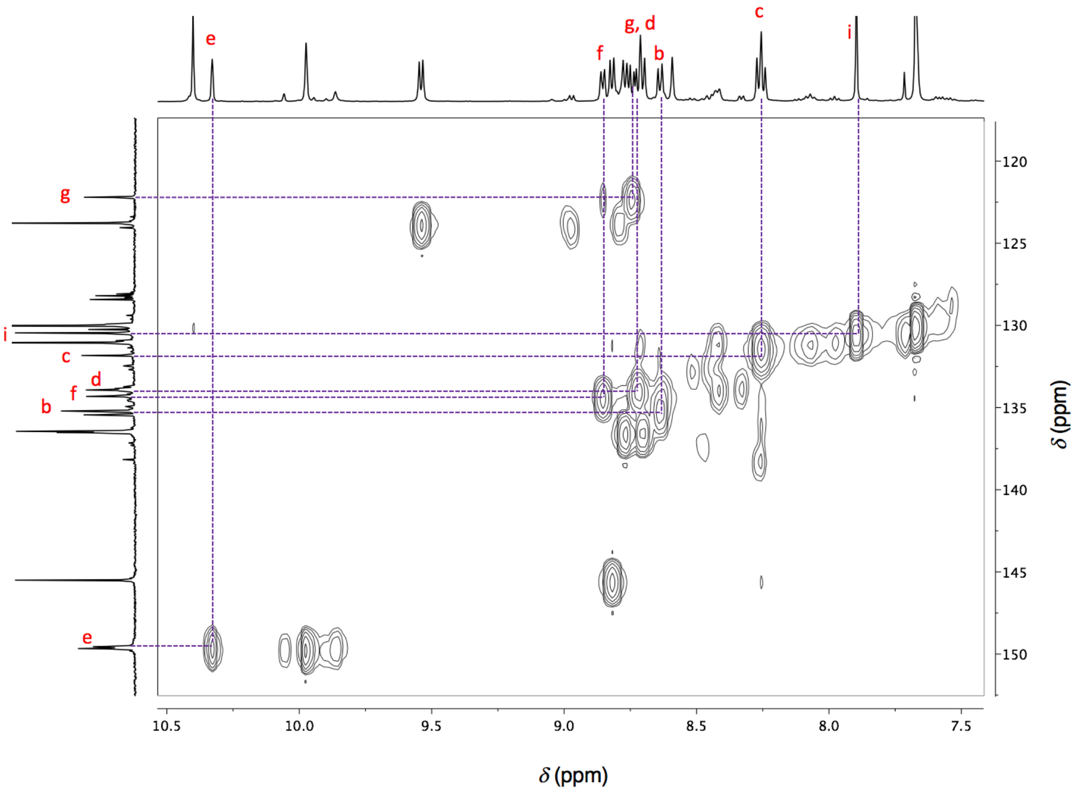

Figure S34. HSQC (D_2O, 125 MHz, 298 K) of the library composed of 5 mM of **A** and 5 mM of $\mathbf{B_7}$. **(a)** Full spectrum and **(b)** expansion of the aromatic region. Correlations between protons and carbons corresponding to [2]catenane $\mathbf{C_7}$ are highlighted with purple doted lines.

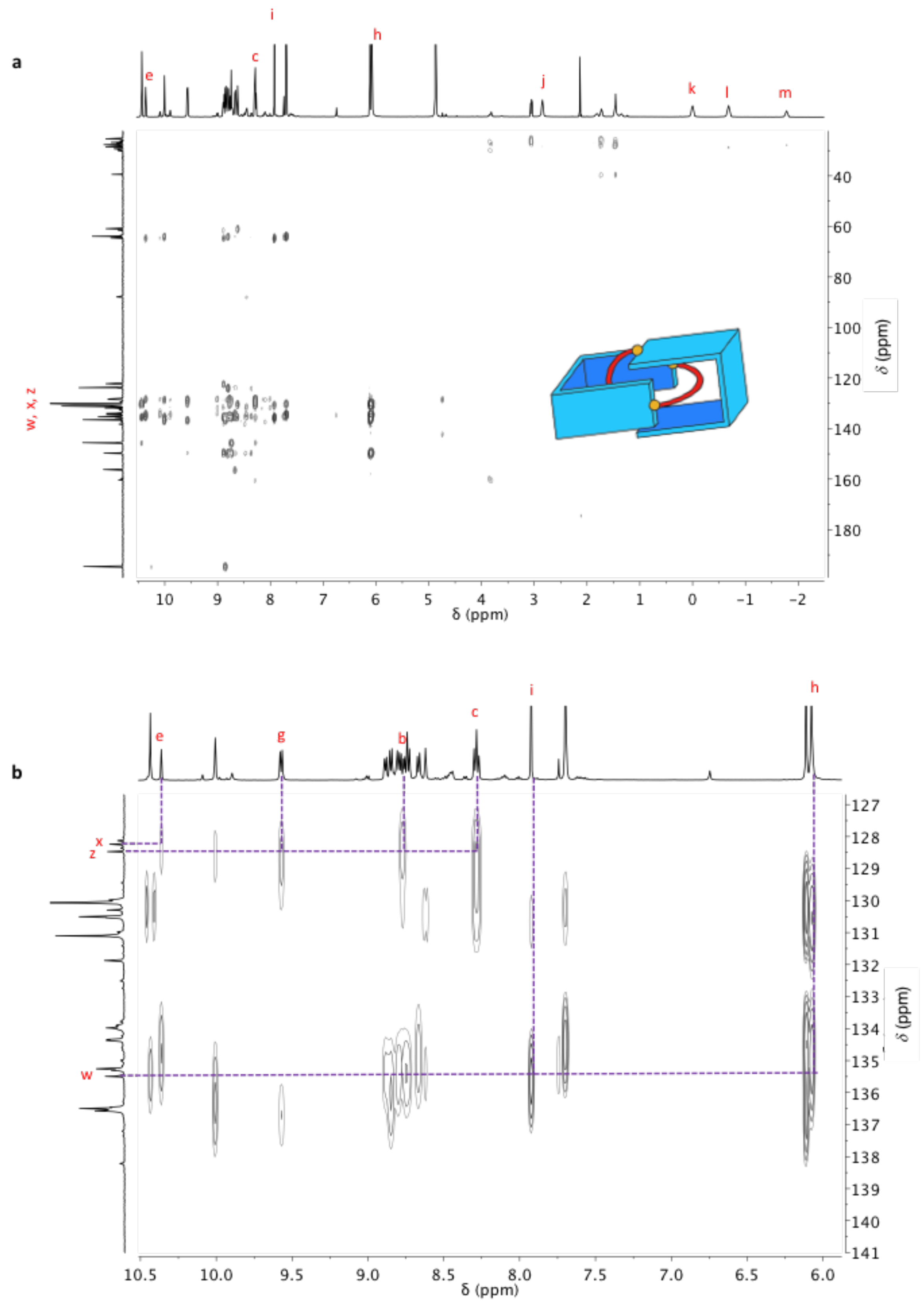

Figure S35. HMBC (D_2O, 125 MHz, 298 K) of the library composed of 5 mM of **A** and 5 mM of $\mathbf{B_7}$. Expansion of the aromatic region. Correlations between protons and carbons corresponding to [2]catenane $\mathbf{C_7}$ are highlighted with purple dotted lines.

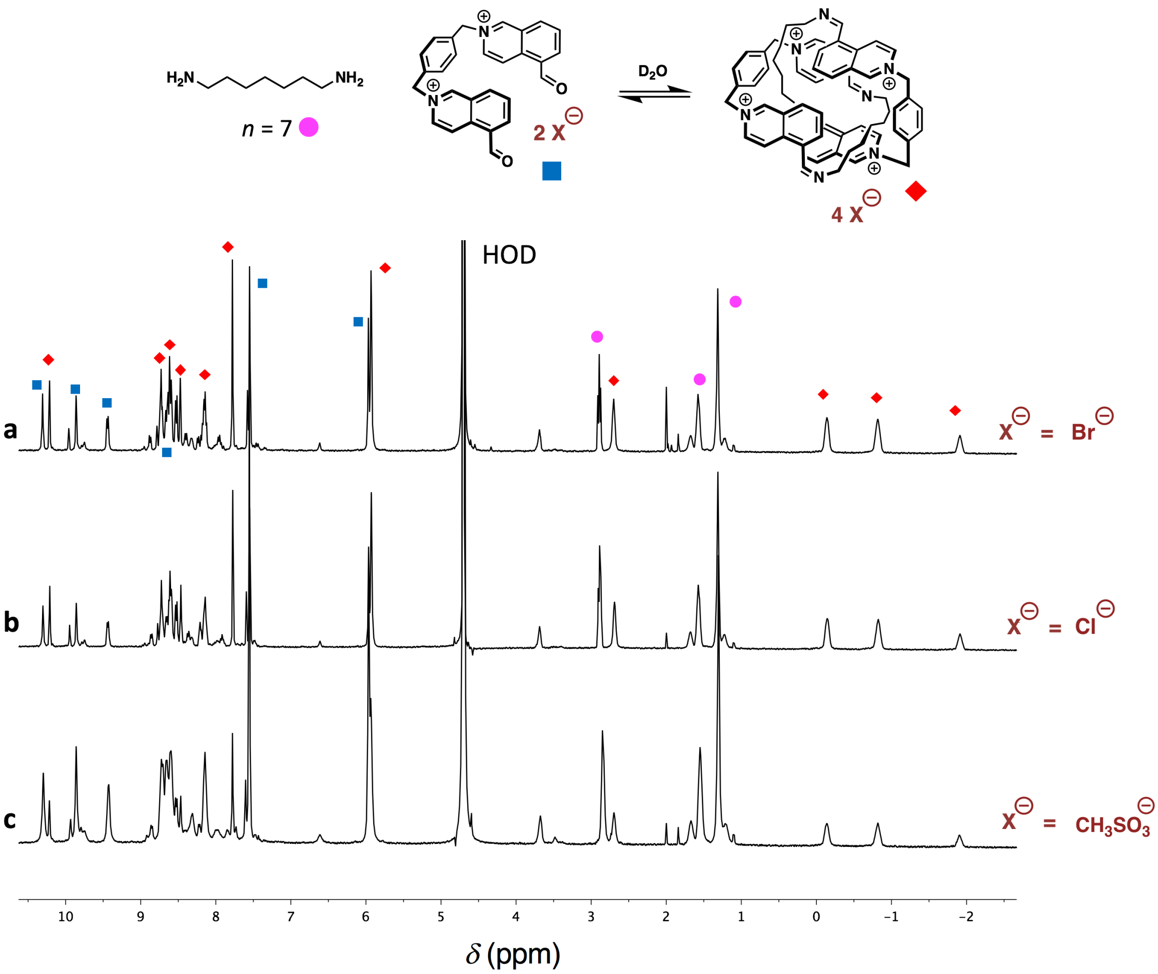

Figure S36. Effect of the counter-ion on the library composition. ^{1}H NMR spectra (D_2O, 400 MHz, 298 K) of the library composed of 5 mM of **A** and 5 mM of **B**$_7$. The counter-ion is: **(a)** bromide, **(b)** chloride and **(c)** methanesulfonate.

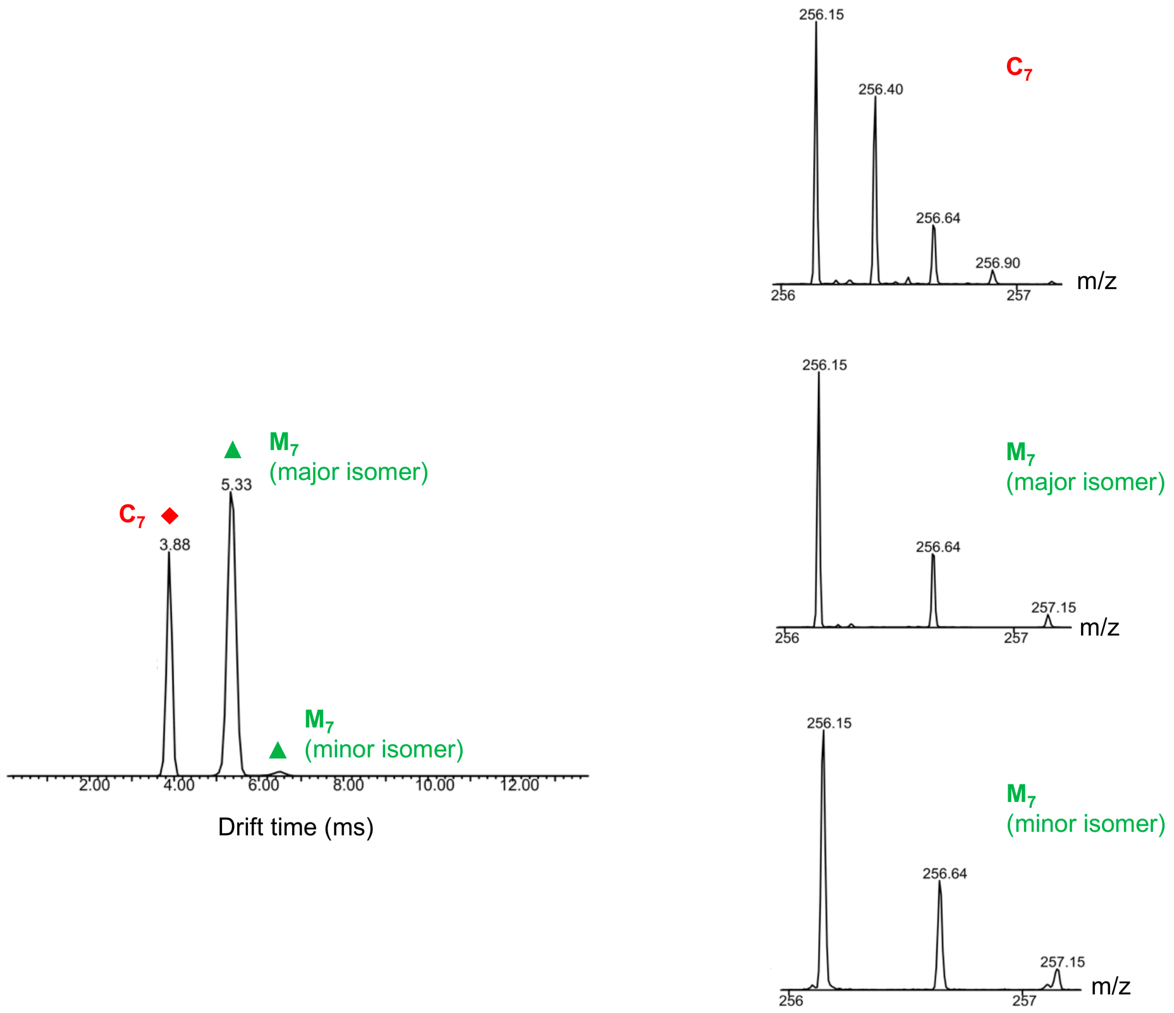

Figure S37. IMS separation of the *m/z* 256.15 peak corresponding to either [2]catenane **C$_7$** or macrocycle **M$_7$**. The mass spectrum of each IMS peak is shown on the right side. The minor isomer of **M$_7$** probably arises from the insource fragmentation of the [2]catenane and corresponds to the open macrocycle.

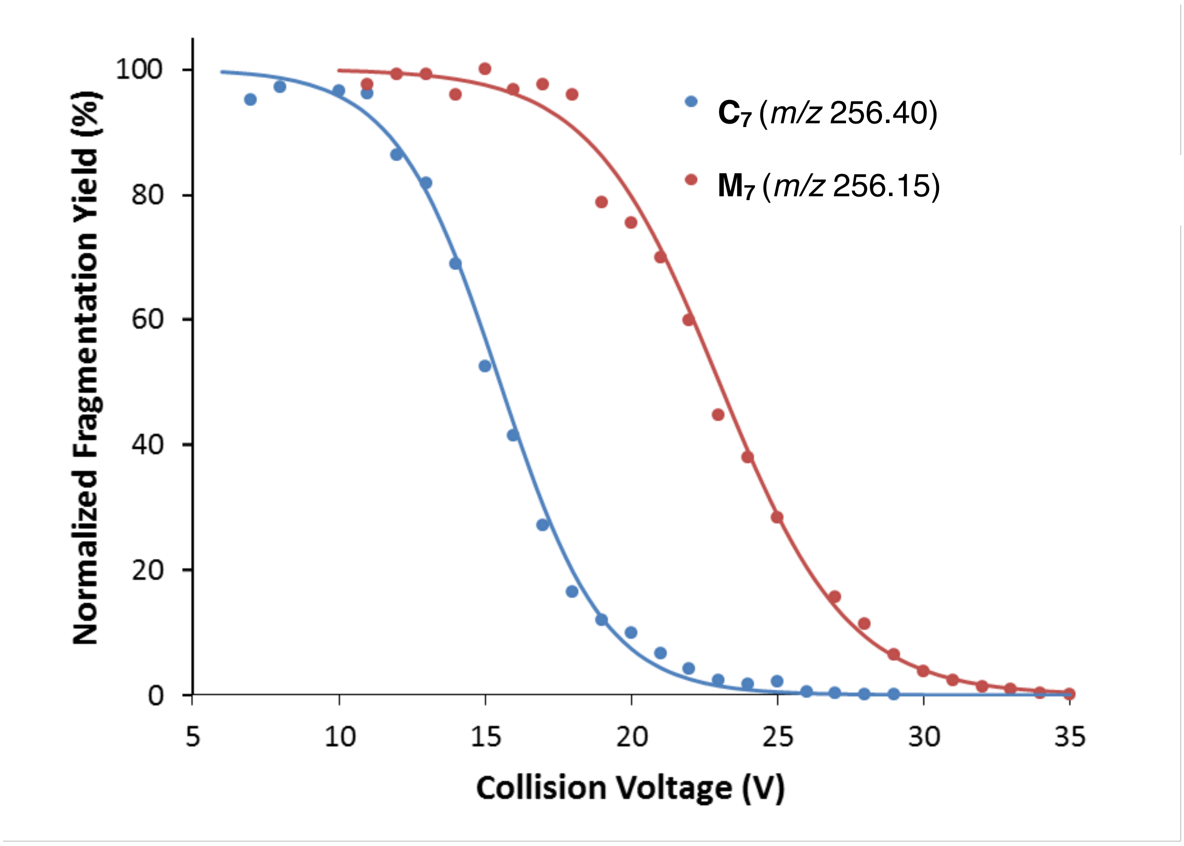

Figure S38. Normalized fragmentation yields (%) *versus* collision energy (V), n = 7. Dots show experimental data points and lines sigmoidal fit. The curves show the fragmentation yield for macrocycle **M**$_7$ (blue) and red for [2]catenane **C**$_7$ (red). For **M**$_7$, we used the isotopic peak m/z 256.15. For the [2]catenane **C**$_7$, we used the isotopic peak *m/z* 256.40. Other libraries showed similar graphs.

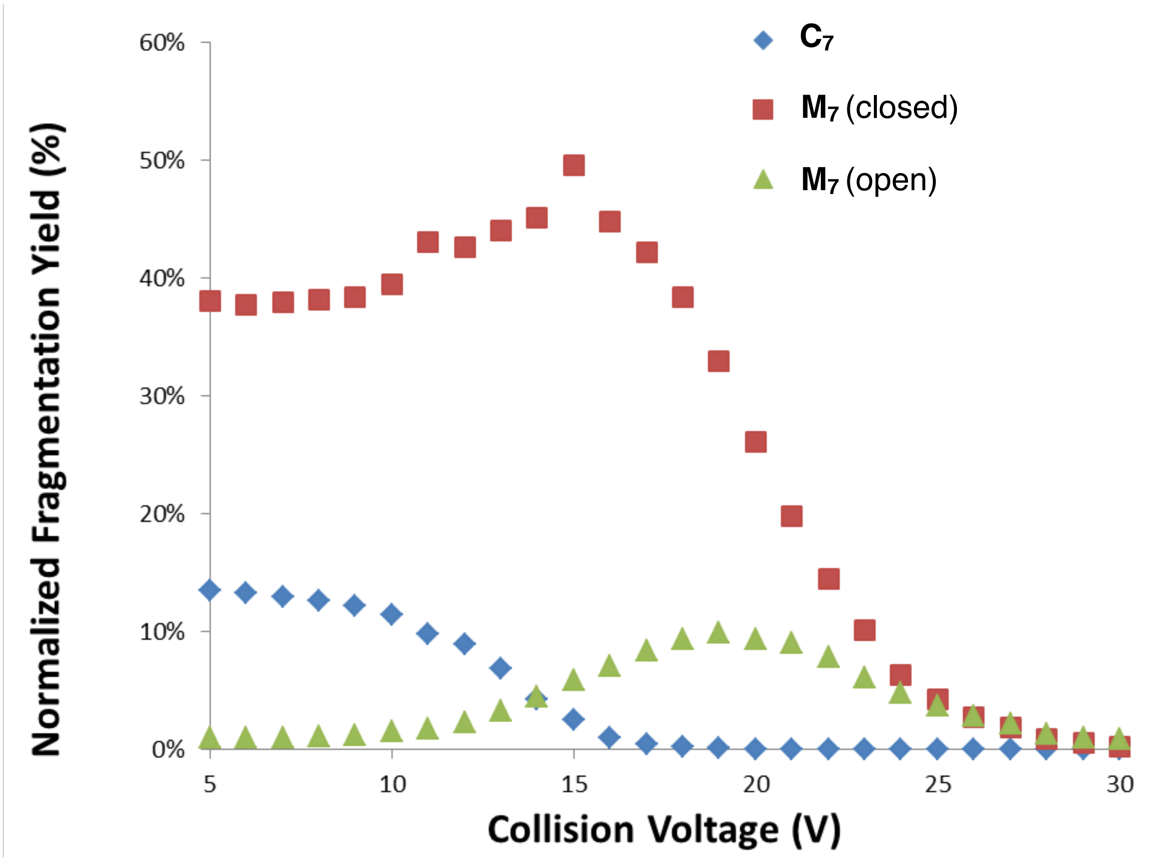

Figure S39. Normalized intensity (%) of [2]catenane, closed and opened [1+1] macrocycles (*m/z* = 256.15) *versus* collision energy (V), *n* = 7. The parent ion with *m/z* = 256.15 was isolated and the fragmentation was carried out before IMS and, therefore, catenane, macrocycles closed and opened could be separated by travelling wave instrument. All peaks were normalized against the total ion current produced.

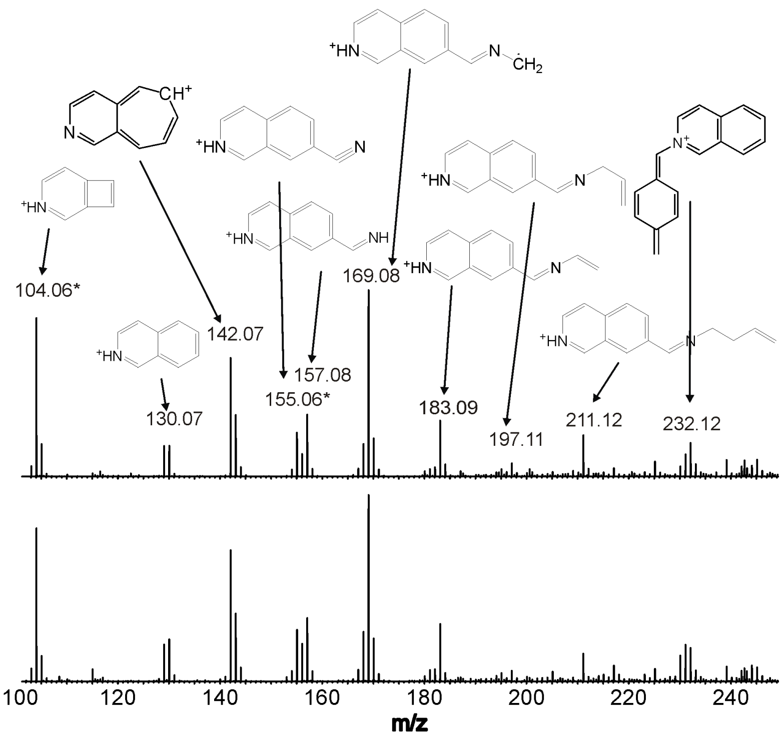

Figure S40. MS/MS of IMS separated [2]catenane **C**$_7$ (up) and macrocycle **M**$_7$ (down) from *m/z* 100 to 250. In this region the fragments observed are identical in the other libraries and do not depend on *n*.

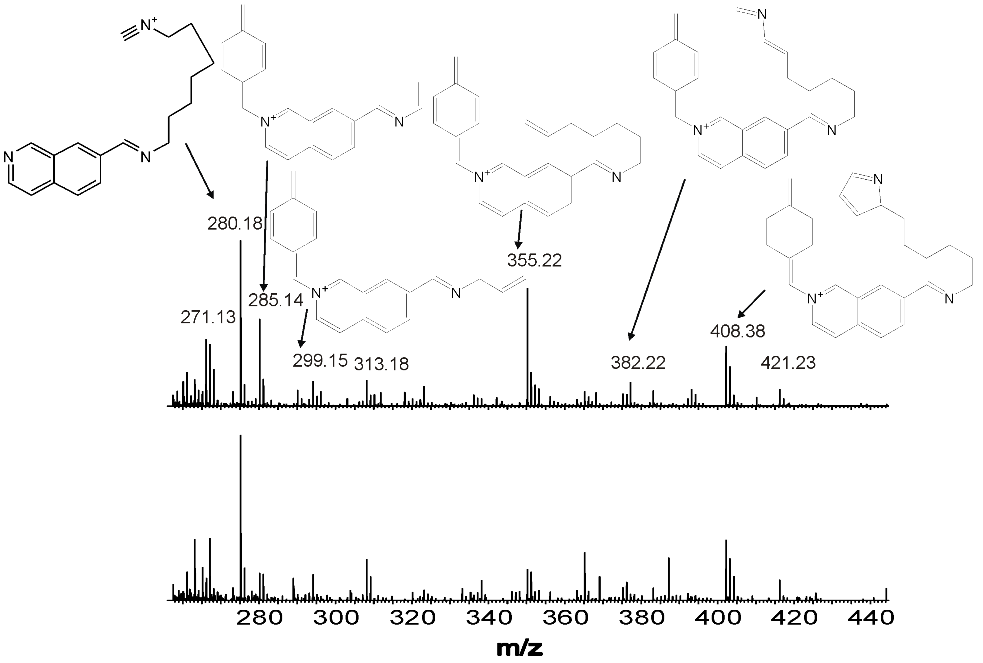

Figure S41. MS/MS of IMS separated [2]catenane **C**$_7$ (up) and macrocycle **M**$_7$ (down) from *m/z* 260 to 450. Fragments with higher *m/z* were not observed. Fragment at *m/z* 408.38 was not observed in other libraries: ions with similar structure but with varying alkyl chain length (depending on *n*) were observed instead.

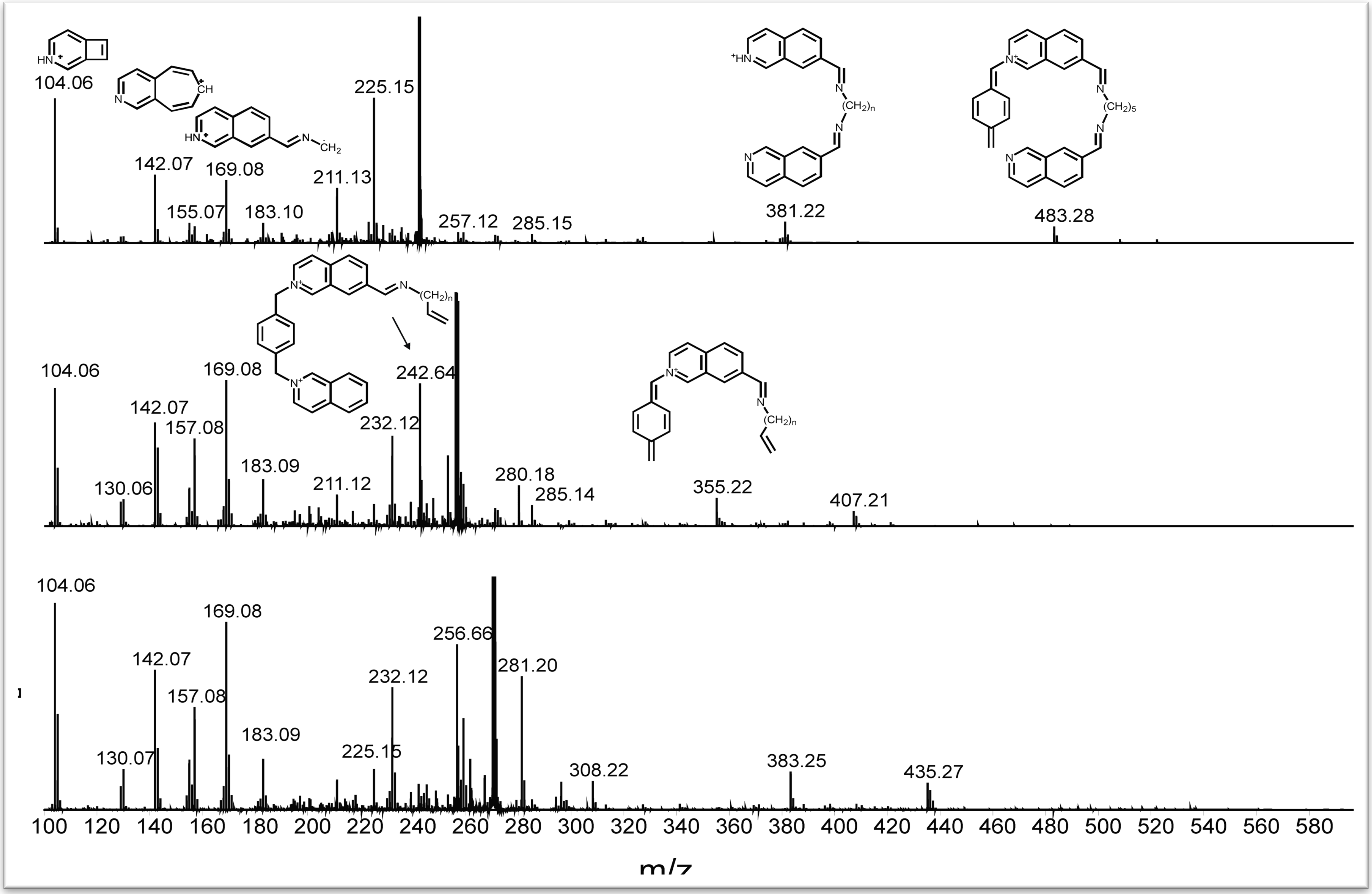

Figure S42.
Comparison of the MS/MS of IMS separated [2]catenanes **C_5** (up), **C_7** (middle) and **C_9** (down) from *m/z* 100 to 600. Fragments with higher *m/z* were not observed. For assignment of the fragments with *m/z* below 200 see Figure S40.

9 Library composed of A and B_8

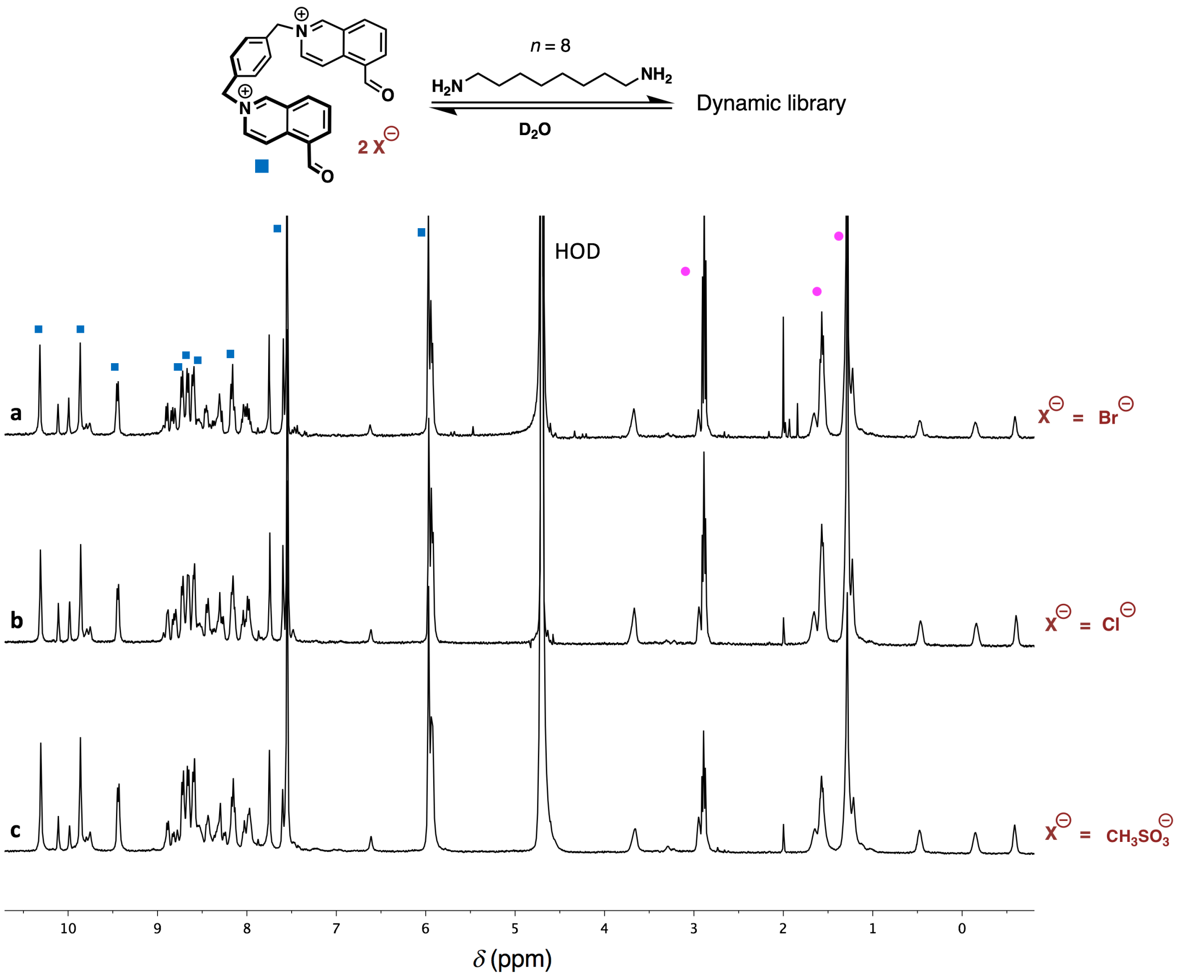

Figure S43. ^{1}H NMR spectra (D_2O, 400 MHz, 298 K) of the library composed of 5 mM of **A** and 5 mM of $\mathbf{B_8}$. The counter-ion is: **(a)** bromide, **(b)** chloride and **(c)** methanesulfonate

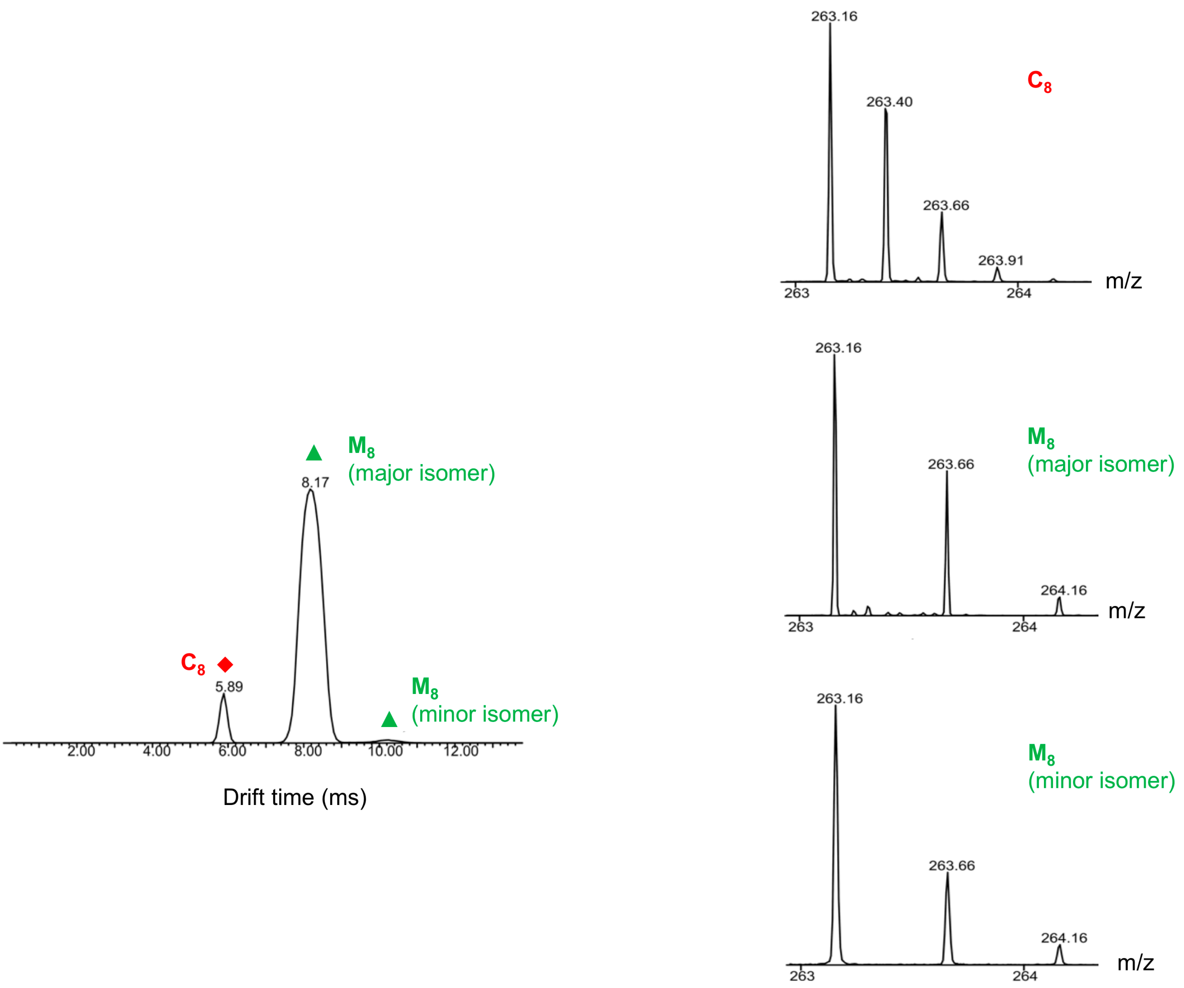

Figure S44. IMS separation of the m/z 263.18 peak corresponding to either [2]catenane $\mathbf{C_8}$ or macrocycle $\mathbf{M_8}$. The mass spectrum of each IMS peak is shown on the right side. The minor isomer of $\mathbf{M_8}$ probably arises from the insource fragmentation of the [2]catenane and corresponds to the open macrocycle.

.

10 Library composed of A and B_9

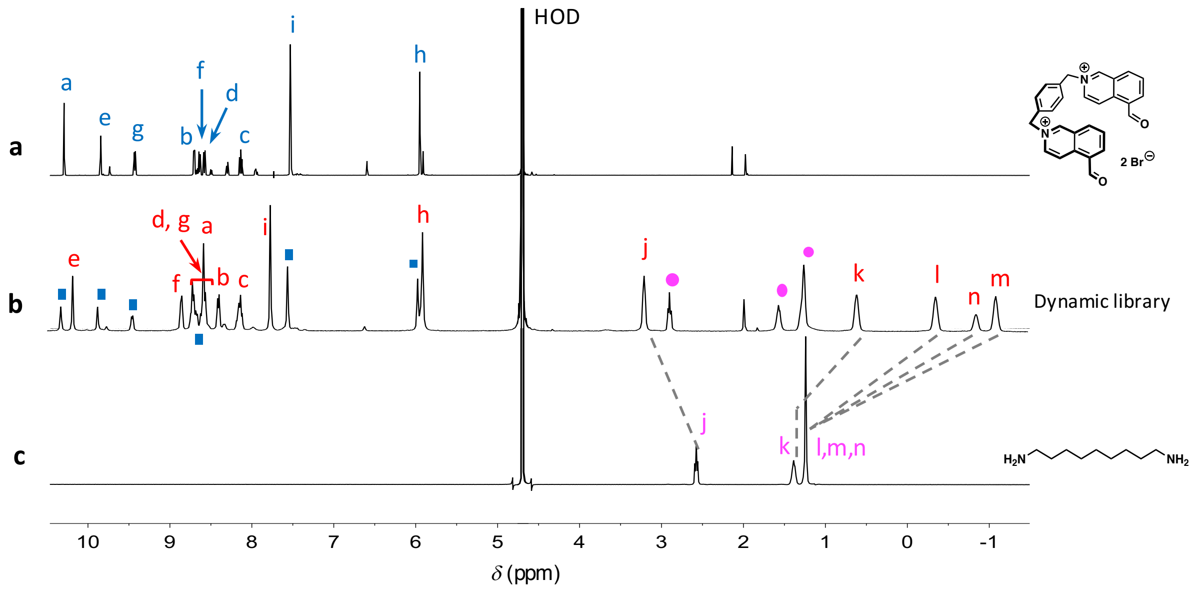

Figure S45. ^{1}H NMR spectra (D_2O, 400 MHz, 298 K) of the library composed of 10 mM of **A** and 10 mM of $\mathbf{B_9}$. Comparison of the starting materials **(a) A** alone and **(c)** $\mathbf{B_9}$ alone with **(b)** the corresponding library. Only the signals corresponding to [2]catenane $\mathbf{C_9}$ were assigned. Residual starting materials are labelled with blue squares (**A**) and pink circles ($\mathbf{B_9}$). The grey dotted lines highlight significant NMR shifts between the starting materials and $\mathbf{C_9}$.

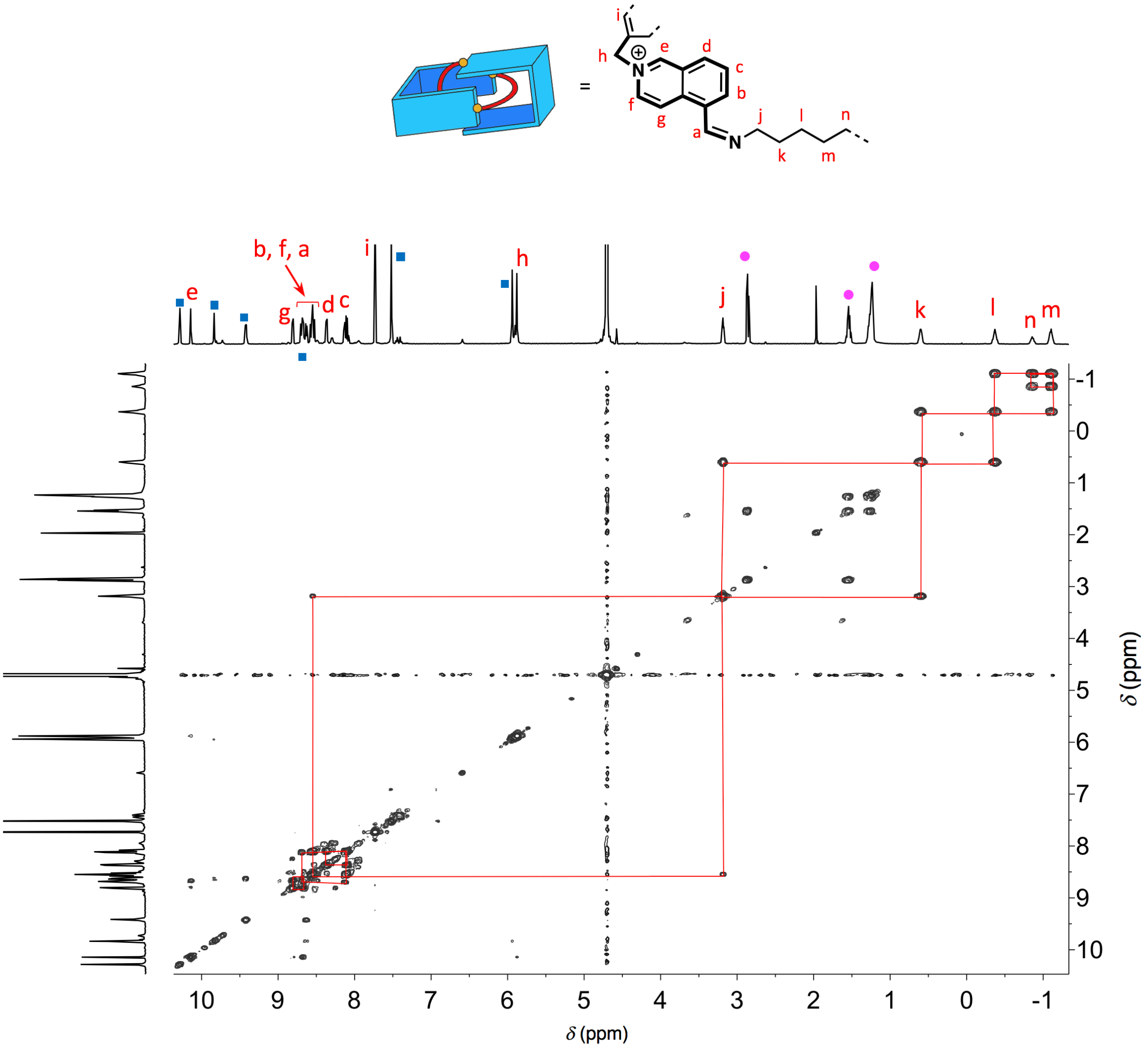

Figure S46. COSY (D_2O, 500 MHz, 298 K) of the library composed of 5 mM of **A** and 5 mM of **B₉**. Correlations associated with **C₉** are highlighted in red.

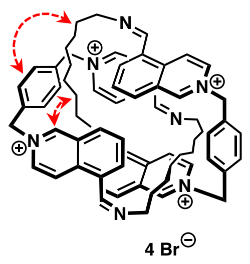

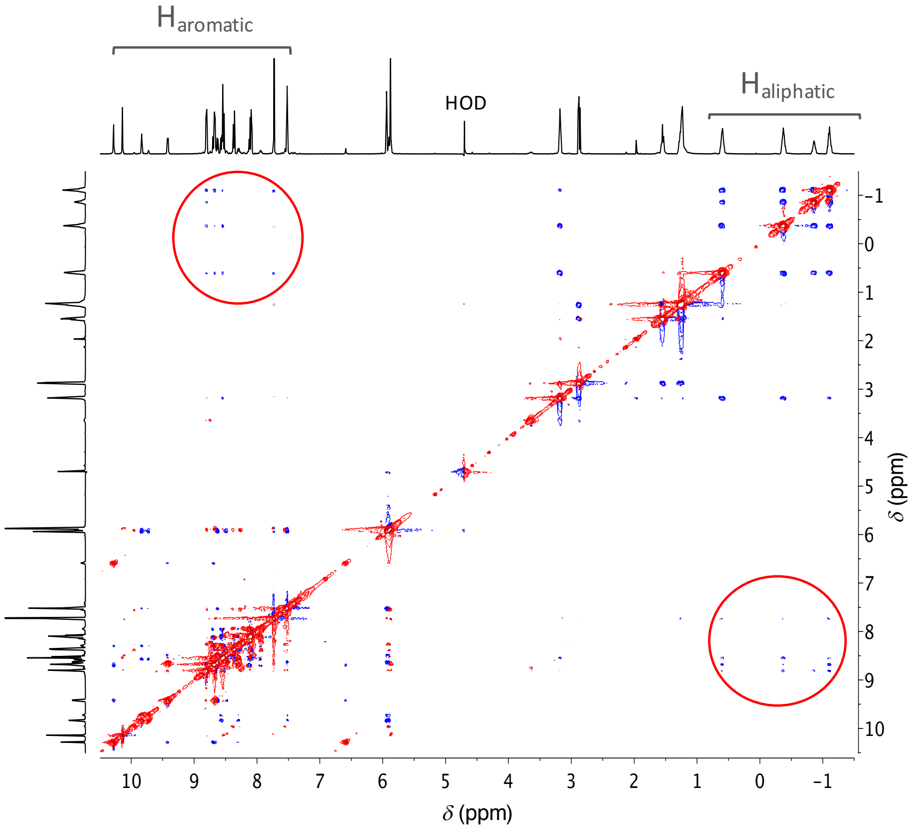

Figure S47. NOESY (D_2O, 500 MHz, 298 K, d_8 = 400 ms) of the library composed of 5 mM of **A** and 5 mM of **B₉**. Correlations in agreement with the proposed structure of **C₉** are highlighted with doted lines.

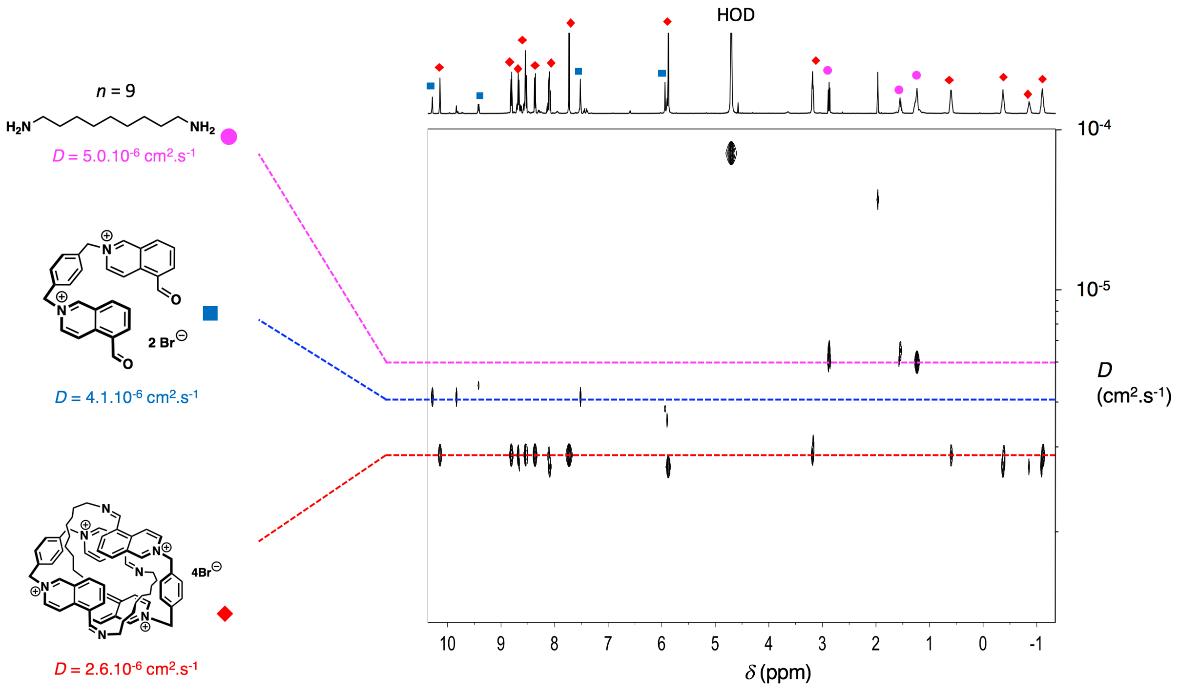

Figure S48. DOSY spectrum (D_2O, 500 MHz, 298 K) of the library composed of 5 mM of **A** and 5 mM of **B₉**. Each species of the library is highlighted with a dotted line. The diffusion coefficients were measured for $\mathbf{B_9}$ ($5.0.10^{-6}$ $cm^2{\cdot}s^{-1}$), $\mathbf{A}^{2+}$ ($4.1.10^{-6}$ $cm^2{\cdot}s^{-1}$) and $\mathbf{C_9}$ ($2.6.10^{-6}$ $cm^2{\cdot}s^{-1}$). Assuming the molecule to be spherical, these values of diffusion coefficients correspond to volumes of $0.18.10^{-27}$, $0.42.10^{-27}$ and $1.18.10^{-27}$ m^3, respectively. Volumes were calculated using the Stokes-Einstein equation at 298 K (k = $1.381.10^{-23}$ $m^2{\cdot}kg{\cdot}s^{-2}{\cdot}K^{-1}$, η_{D2O} = $1.25.10^{-3}$ $kg{\cdot}m^{-1}{\cdot}s^{-1}$).

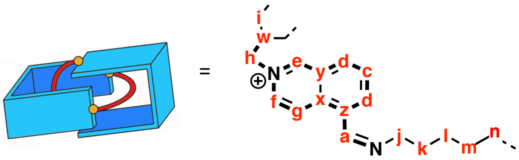

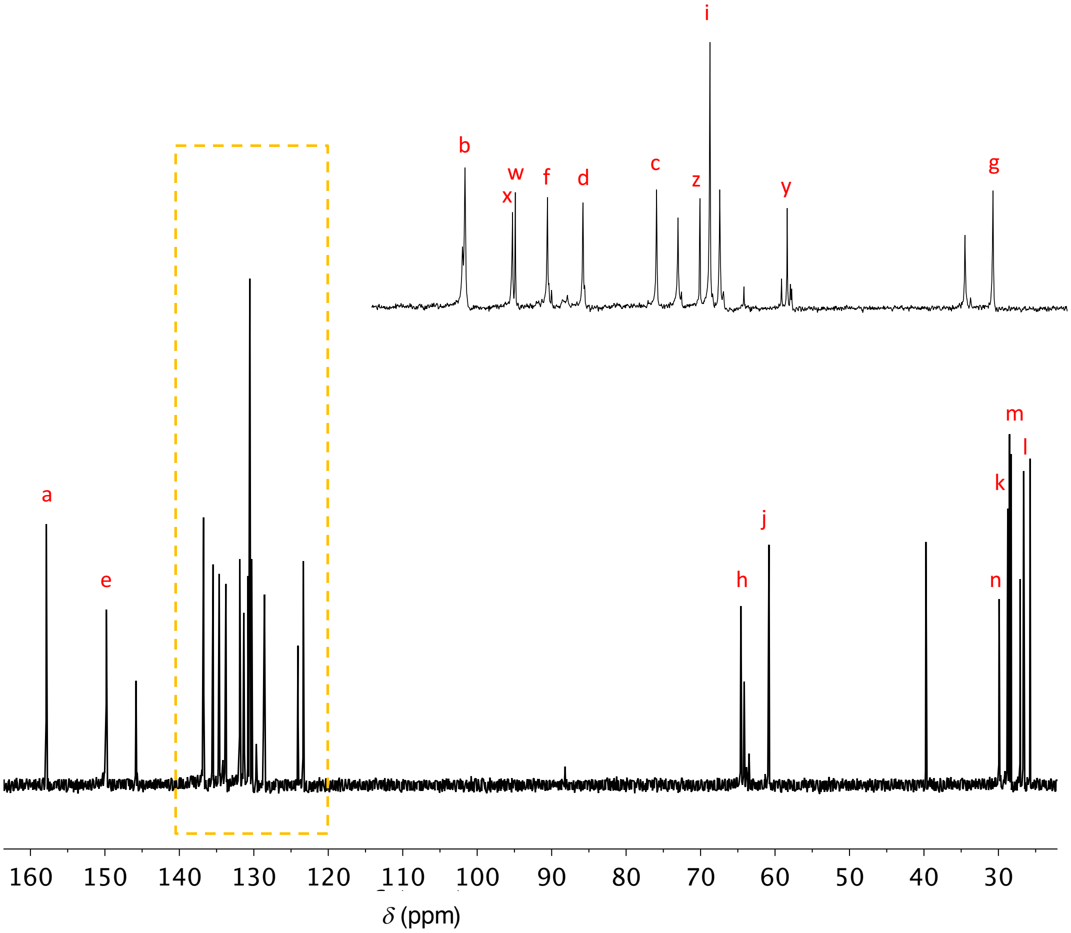

Figure S49. ^{13}C NMR (D_2O, 125 MHz, 298 K) of the library composed of 5 mM of **A** and 5 mM of $\mathbf{B_9}$. Only the signals corresponding to [2]catenane $\mathbf{C_9}$ were assigned. The red insert shows an expansion of the aromatic region.

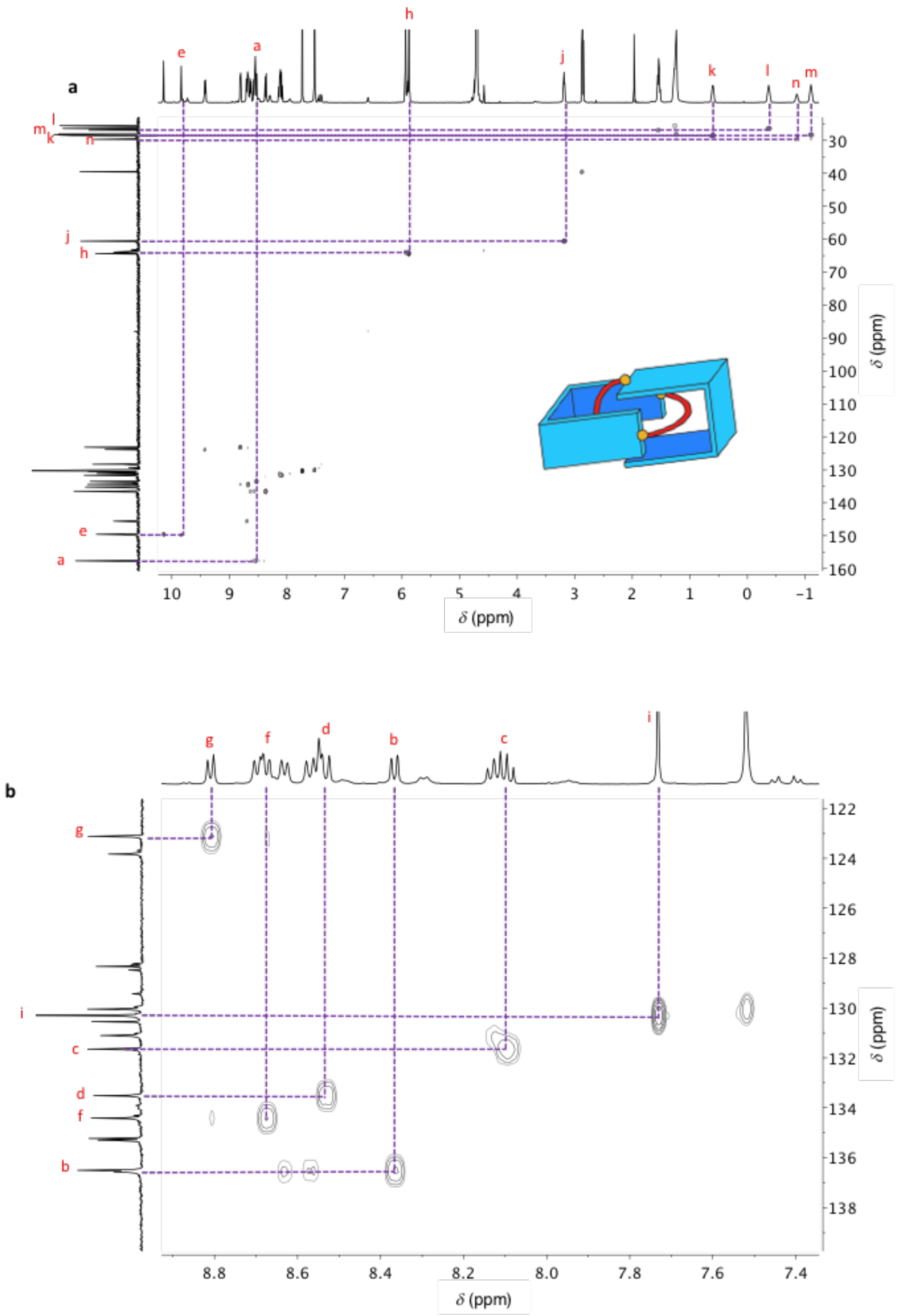

Figure S50. HSQC (D_2O, 125 MHz, 298 K) of the library composed of 5 mM of **A** and 5 mM of **B_9**. **(a)** Full spectrum and **(b)** expansion of the aromatic region. Correlations between protons and carbons corresponding to [2]catenane **C_9** are highlighted with purple dotted lines.

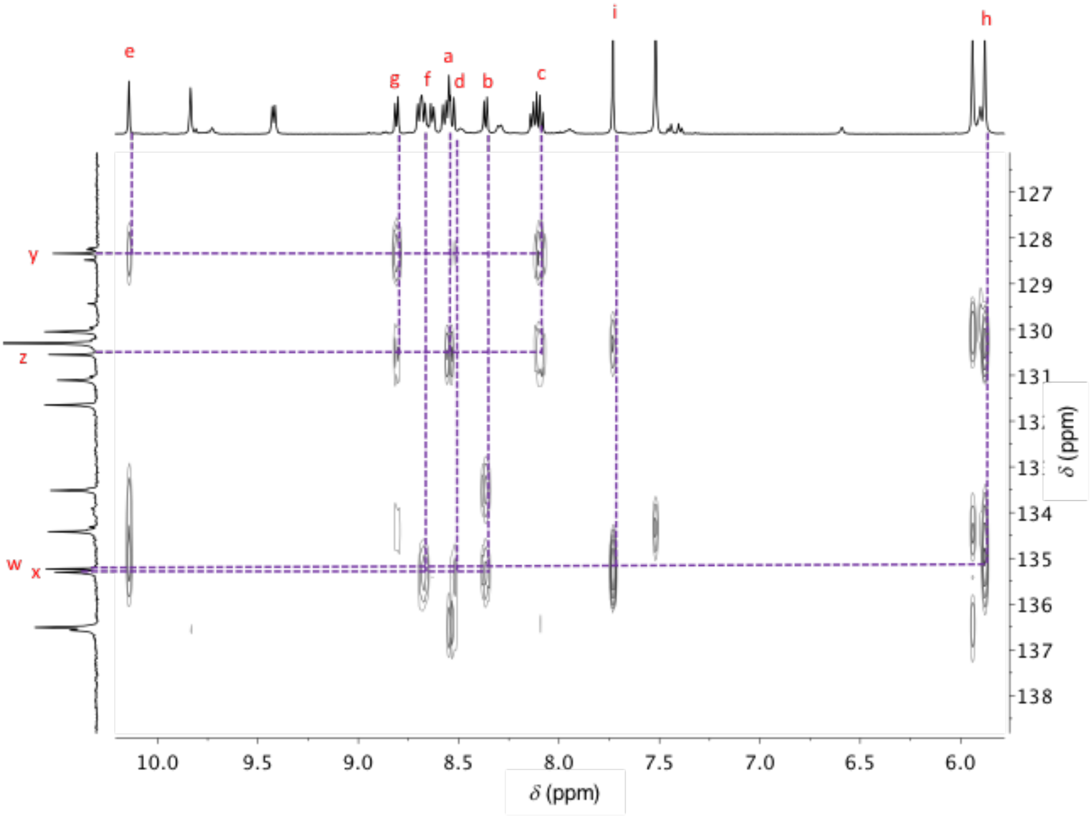

Figure S51. HMBC (D_2O, 125 MHz, 298 K) of the library composed of 5 mM of **A** and 5 mM of **B$_9$**. Expansion of the aromatic region. Correlations between protons and carbons corresponding to [2]catenane **C$_9$** are highlighted with purple dotted lines.

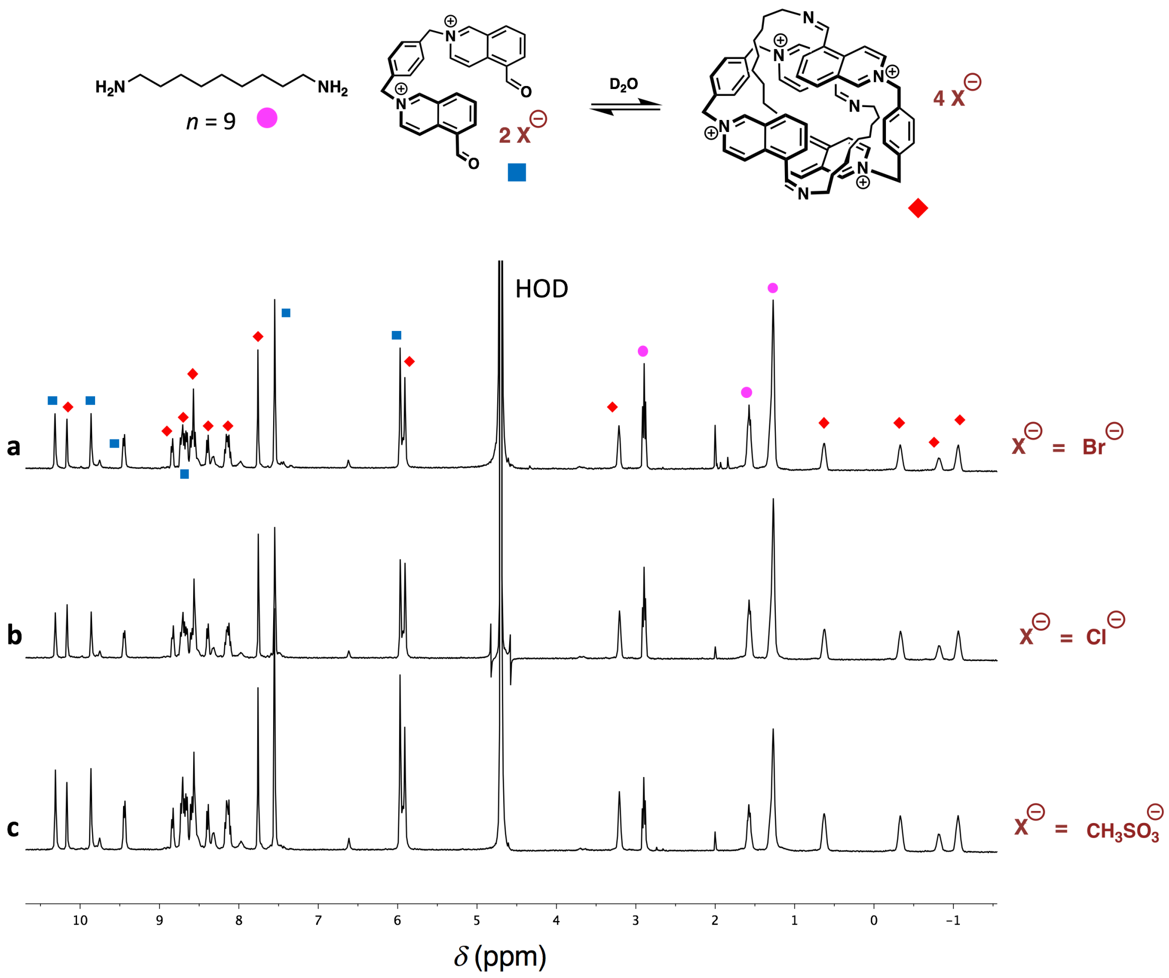

Figure S52. Effect of the counter-ion on the library composition. ^{1}H NMR spectra (D_2O, 400 MHz, 298 K) of the library composed of 5 mM of **A** and 5 mM of **B₉**. The counter-ion is: **(a)** bromide, **(b)** chloride and **(c)** methanesulfonate.

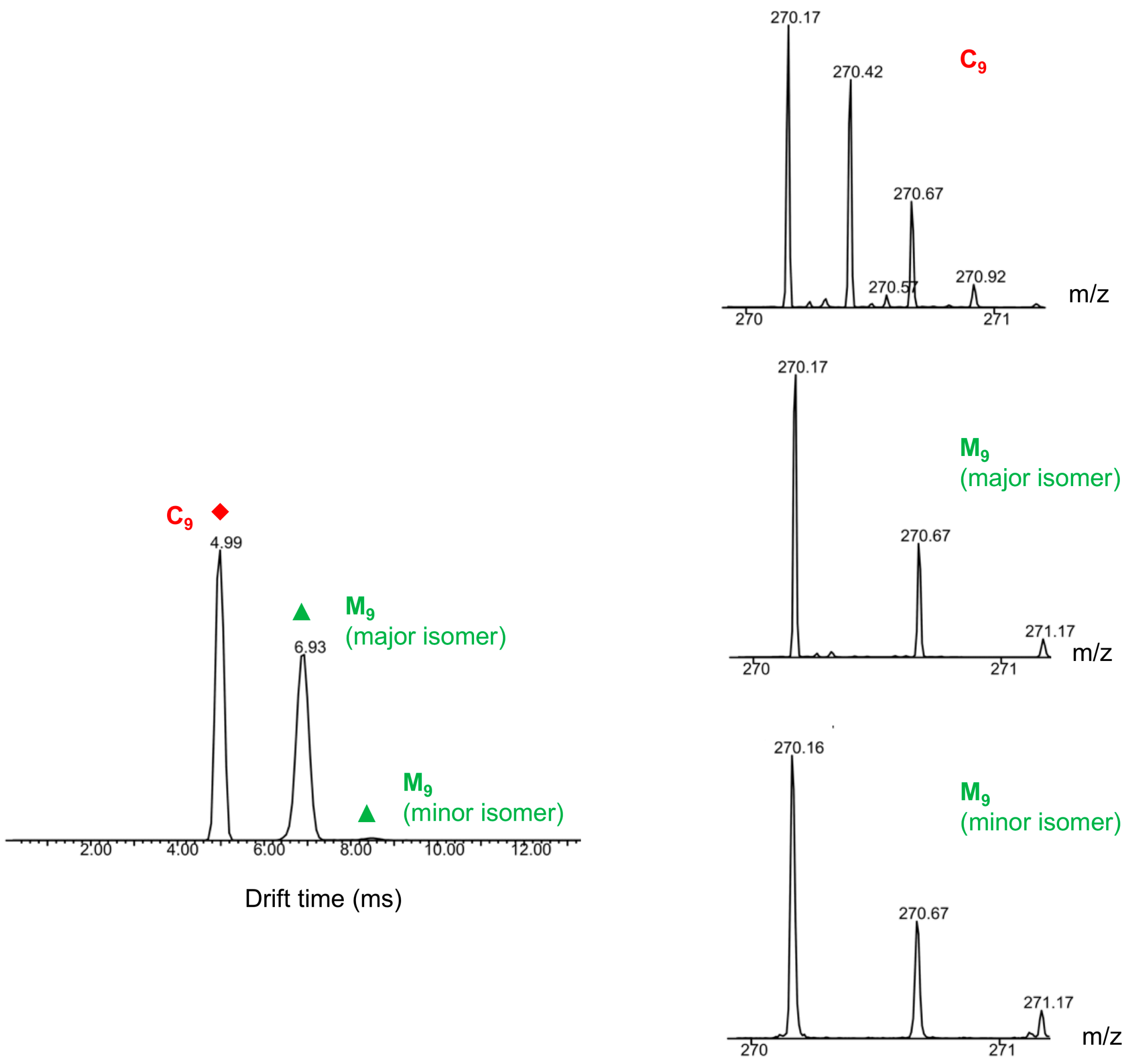

Figure S53. IMS separation of the *m/z* 270.16 peak corresponding to either [2]catenane **C9** or macrocycle **M9**. The mass spectrum of each IMS peak is shown on the right side. The minor isomer of **M9** probably arises from the insource fragmentation of the [2]catenane and corresponds to the open macrocycle.

11 Re-organization of the aqueous libraries containing [2]catenanes upon addition of organic solvents

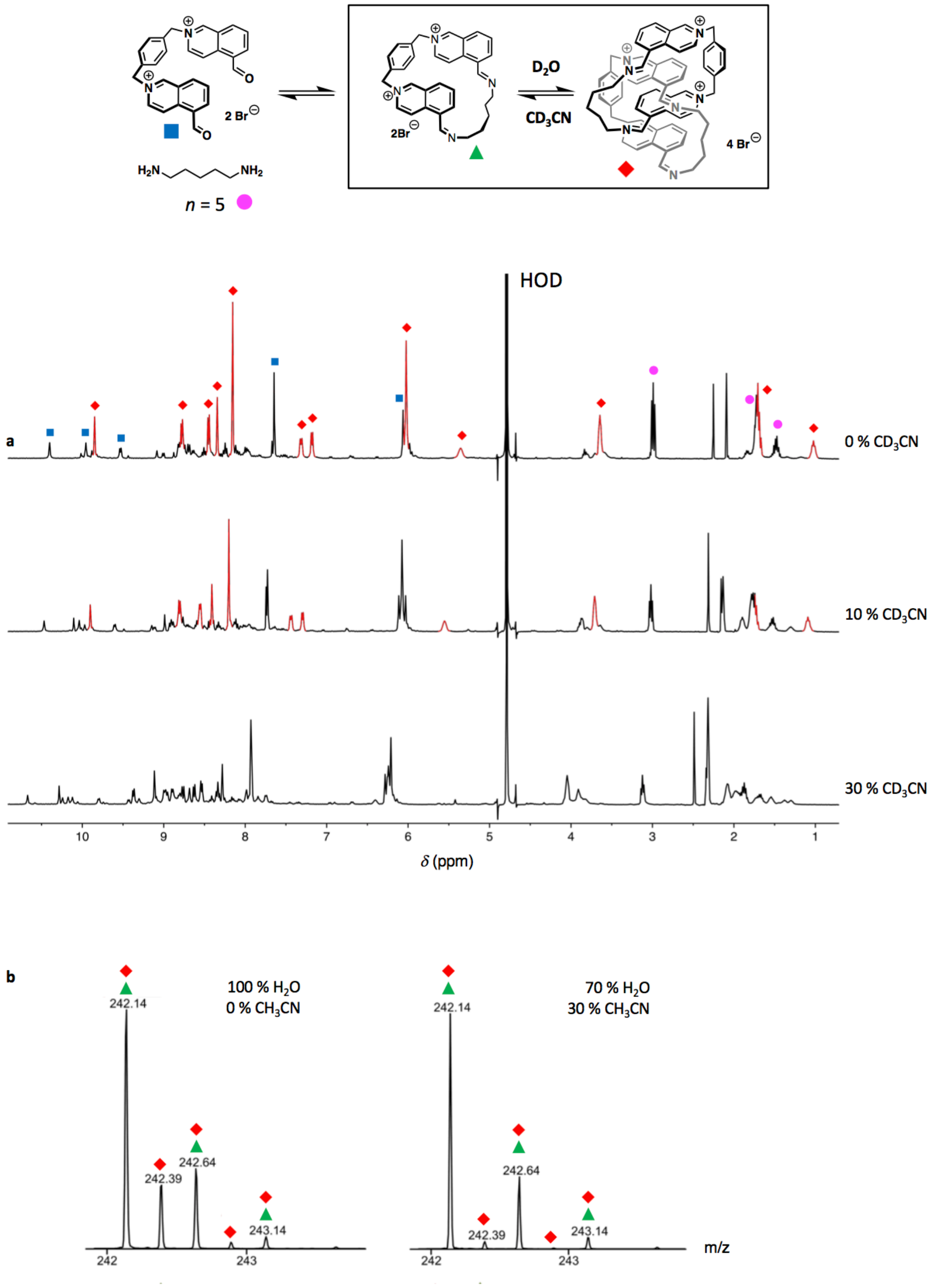

Figure S54. (a) ^{1}H NMR spectra (400 MHz, 298 K) and (b) mass spectra of the library composed of 10 mM of **A** and 10 mM of $\mathbf{B_5}$ in D_2O/CD_3CN.

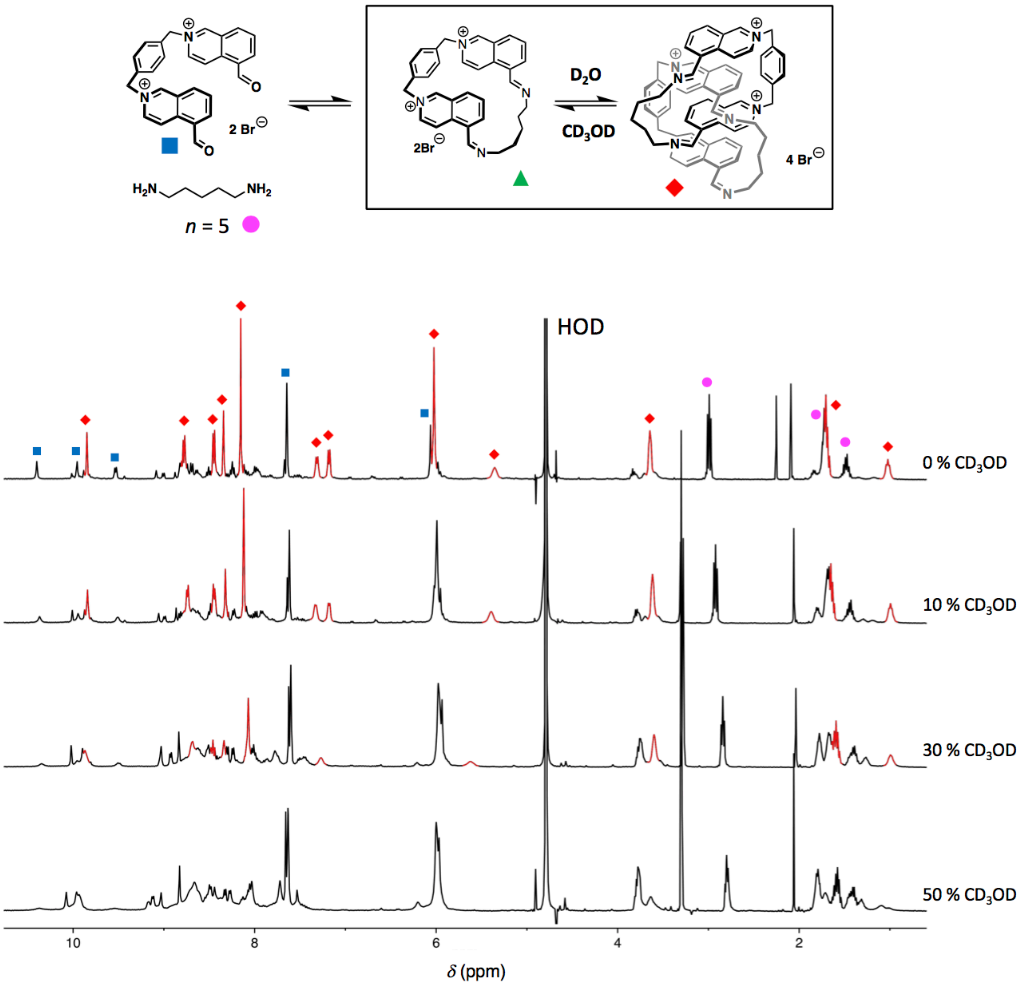

Figure S55. ^{1}H NMR spectra (400 MHz, 298 K) of the library composed of 10 mM of **A** and 10 mM of $\mathbf{B_5}$ in D_2O/CD_3OD.

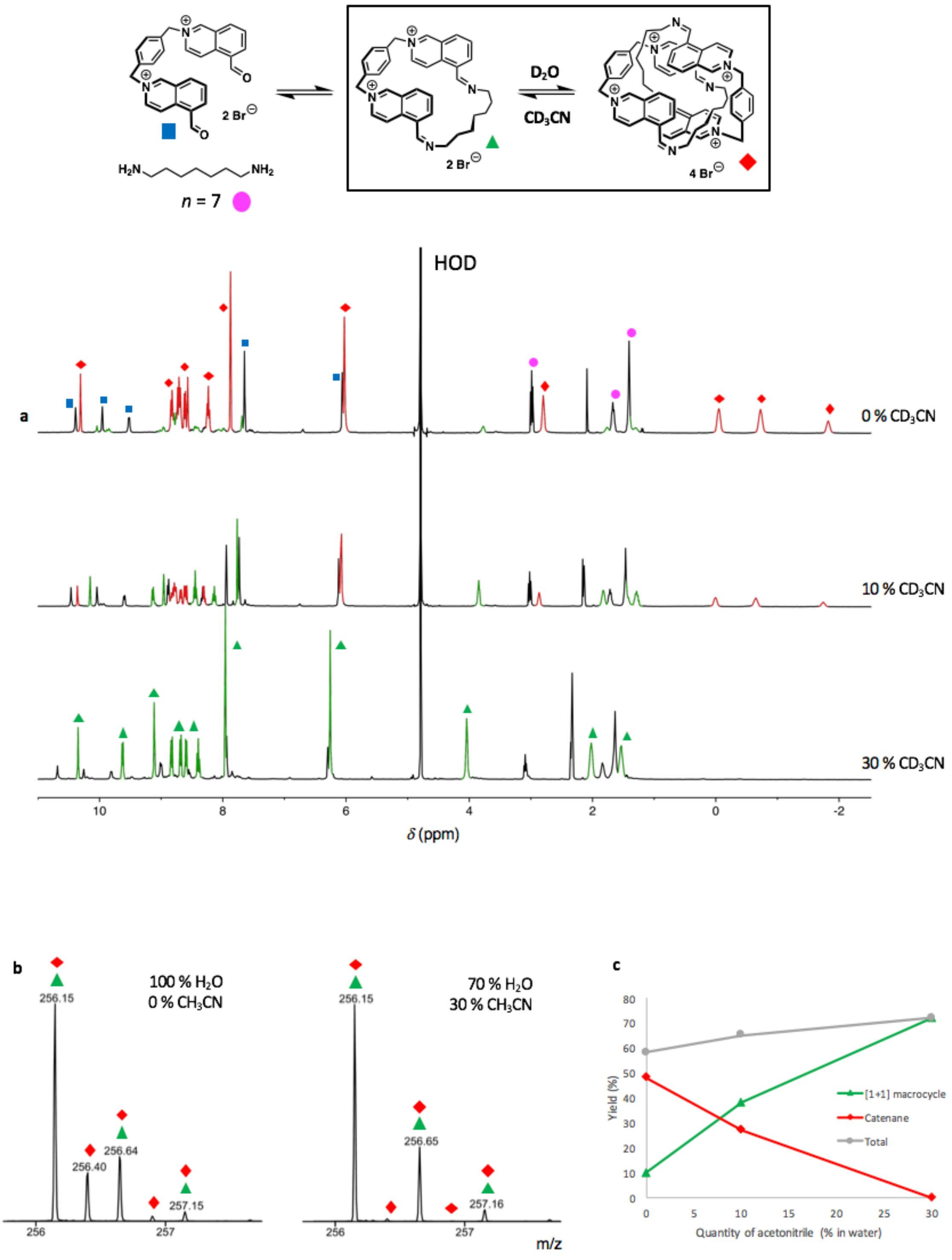

Figure S56. (a) ^{1}H NMR spectra (400 MHz, 298 K) and (b) mass spectra of the library composed of 10 mM of **A** and 10 mM of $\mathbf{B_7}$ in D_2O/CD_3CN.

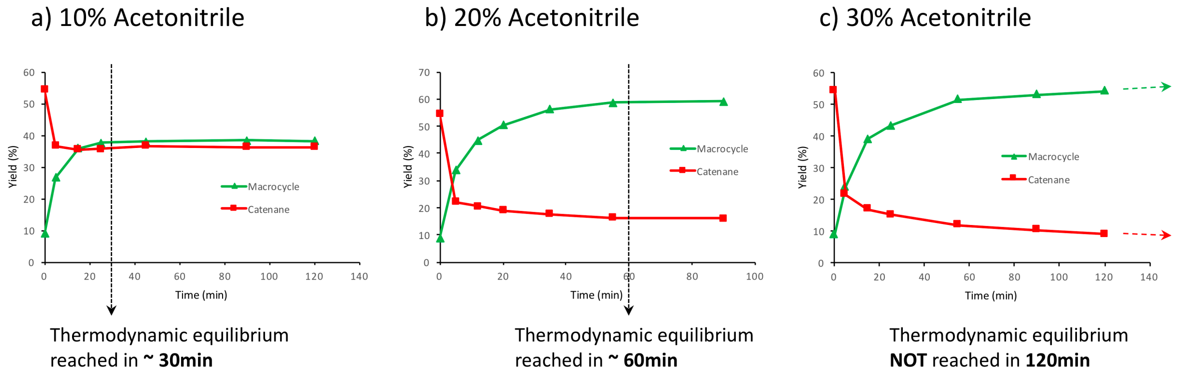

Figure S57. The kinetics of equilibration varies depending on the ratio water/acetonitrile. In this experiment, the catenane was pre-formed in pure water and acetonitrile is subsequently added (final concentrations: 10 mM of **A** and 10 mM of $\mathbf{B_7}$). The conversion of the catenane $\mathbf{C_7}$ into the macrocycle $\mathbf{M_7}$ was measured by NMR over time. As the amount of acetonitrile increases, the kinetics of equilibration slows down and thermodynamic equilibrium is reached after about 30 min (10% CD_3CN), 60 min (20% CD_3CN) and >120 min (30% CD_3CN).

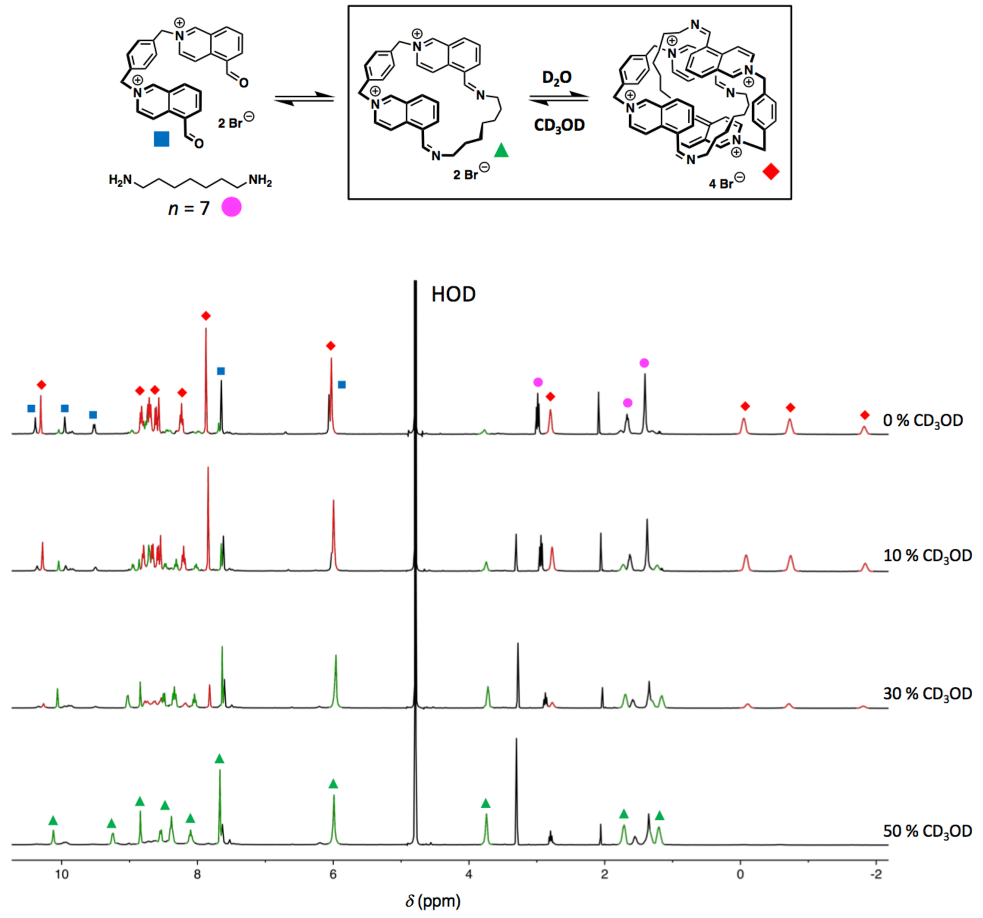

Figure S58. ^{1}H NMR spectra (400 MHz, 298 K) of the library composed of 10 mM of **A** and 10 mM of $\mathbf{B_7}$ in D_2O/CD_3OD.

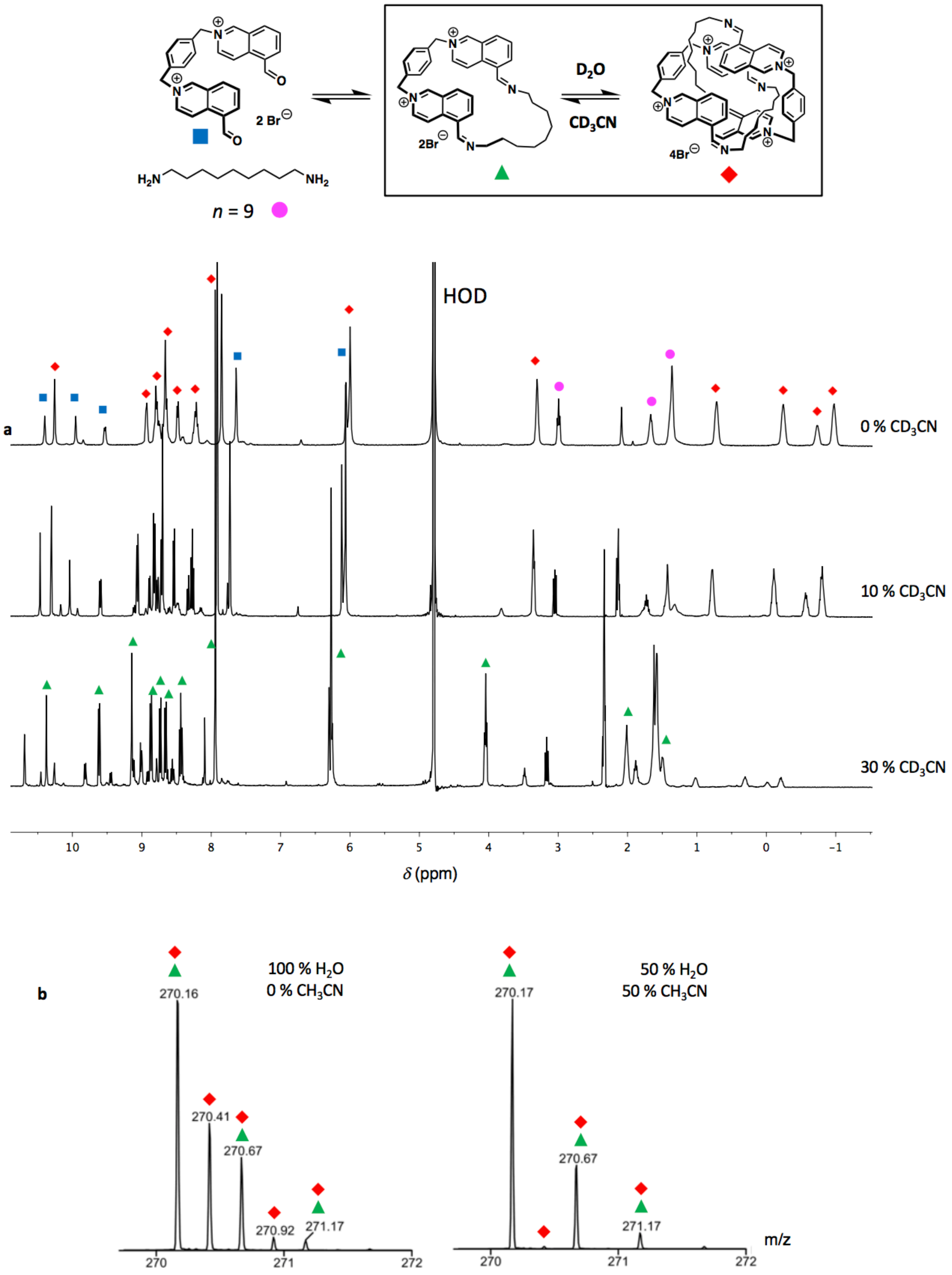

Figure S59. (a) ^{1}H NMR spectra (400 MHz, 298 K) and (b) mass spectra of the library composed of 10 mM of **A** and 10 mM of $\mathbf{B_9}$ in D_2O/CD_3CN.

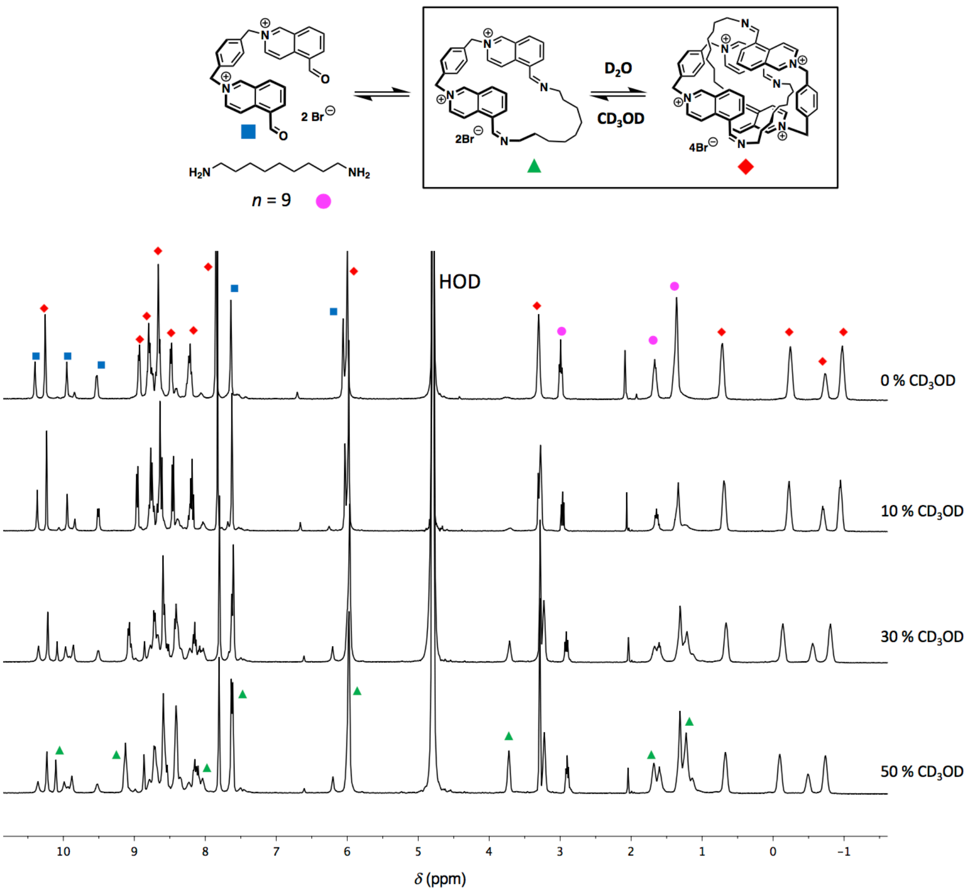

Figure S60. ^{1}H NMR spectra (400 MHz, 298 K) of the library composed of 10 mM of **A** and 10 mM of $\mathbf{B_9}$ in D_2O/CD_3OD.

12 Deuterium exchange occurring on the isoquinolinium unit in D_2O

A decrease of intensity of signal **e** born by the isoquinolinium unit was consistently observed over time in the imine-based libraries presented in this work, and was attributed to deuterium exchange occurring in basic conditions.[S1,S2] Figure S61-62 present experiments performed on building block **A1** aiming at confirming deuterium exchange. Exchange was specifically observed at this position.

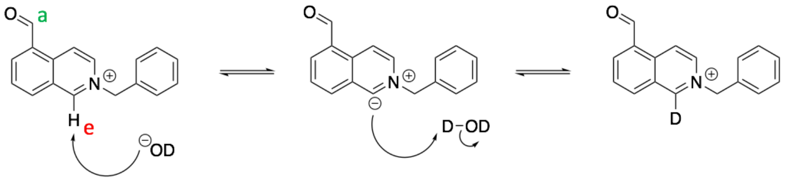

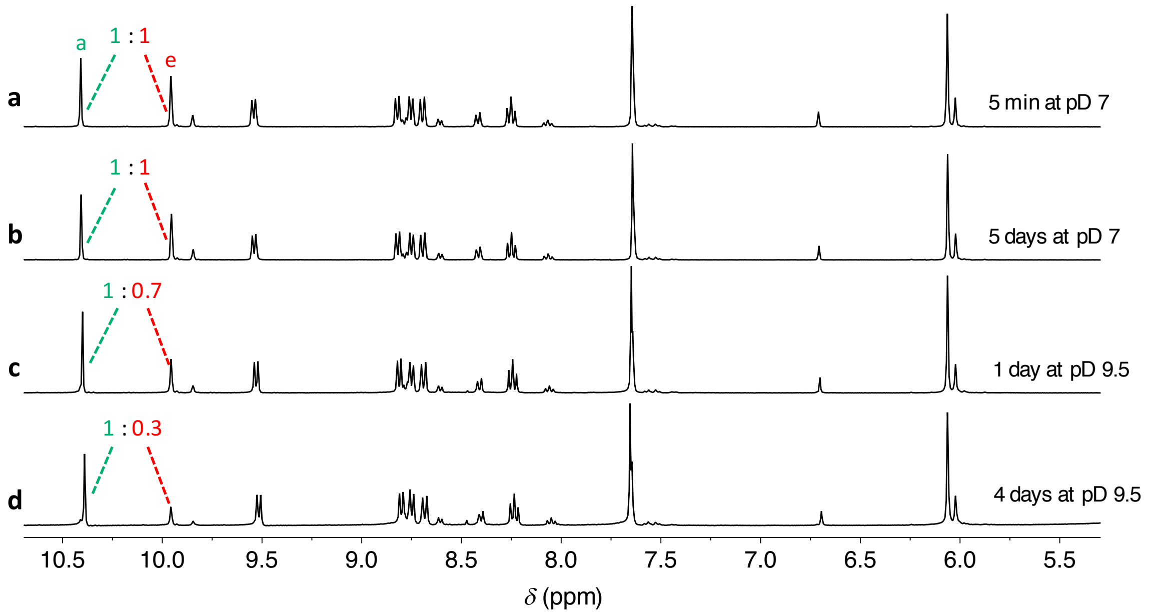

Figure S61. The ^{1}H NMR signal of proton e decreases over time in basic conditions. Partial ^{1}H NMR spectra (D_2O, 400 MHz, 298 K) of **A1** in D_2O in neutral conditions (pD = 7) after **(a)** 5 minutes and **(b)** 5 days, showing that the signal corresponding to proton **e** remains identical. In contrast, in basic conditions (pD = 9.5), the intensity of this signal decreases over time: **(c)** 1 day and **(d)** 4 day. In the figure, protons **a** and **e** are highlighted in green and red respectively.

S1. Zoltewicz, J. A.; Sale, A. A. *J. Am. Chem. Soc.* **1973**, *95*, 3928-3931.
S2. Bagley, M. C.; Alnomsy, A.; Sharhan, H. I. *Synlett* **2016**, *27*, 2467-2472.

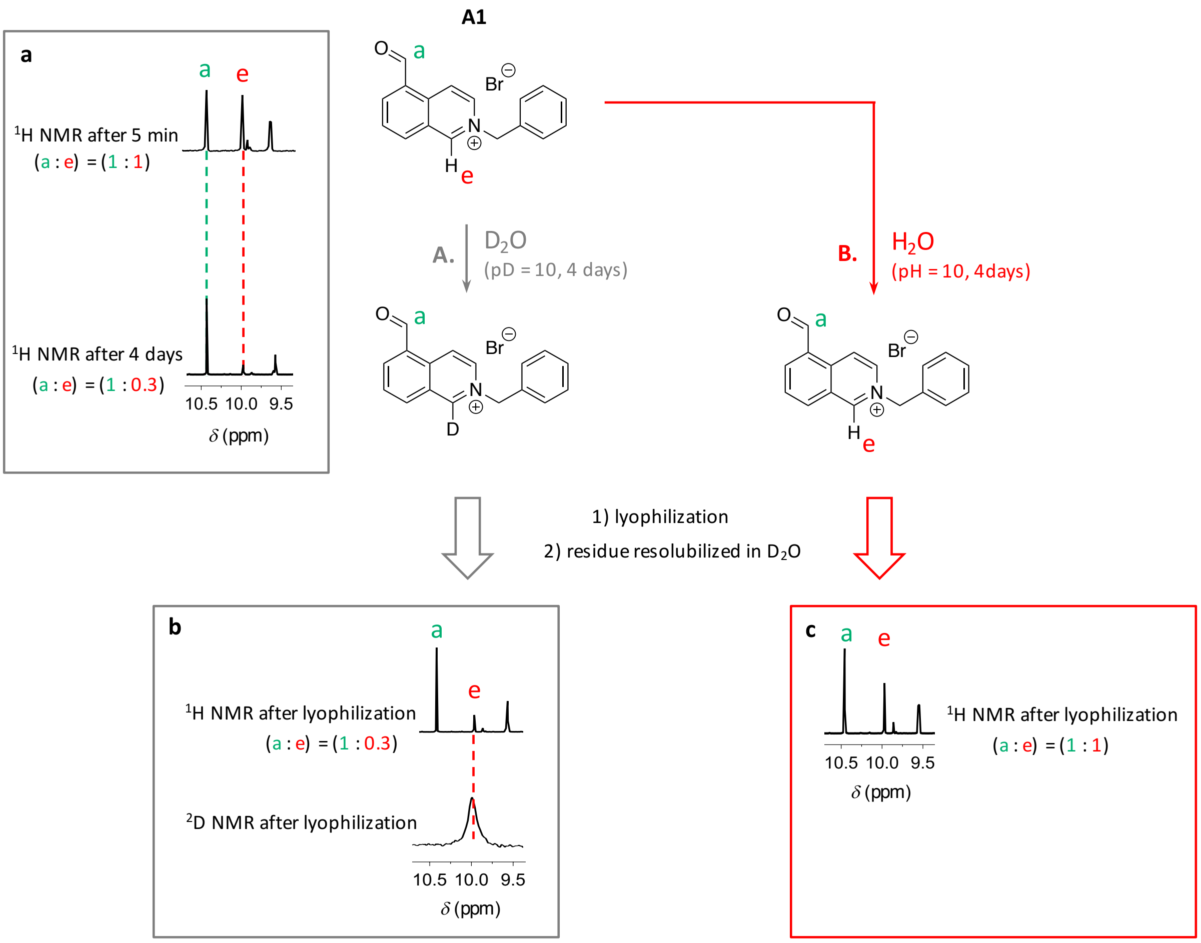

Figure S62. Proton **e** exchanges with deuterium in D_2O. This figure describes an experiment aiming at demonstrating that proton **e** exchanges with deuterium. Building block **A1** was dissolved in D_2O (10 mM) and pD was adjusted to 10 using a concentrated solution of NaOD in D_2O (reaction A in grey). **(a)** The expected decrease of intensity of proton **e** was followed by ^1H NMR over 4 days. In parallel, the same reaction was performed in H_2O and the pH was adjusted using NaOH (reaction B in red). In a protonated solvent, deuterium exchange cannot occur. Both reactions (A and B) were treated in the same way. The samples were lyophilised and the residues were dissolved in D_2O in order to record their respective NMR spectra. **(b)** At the end of reaction A, the relative intensity of the ^1H signal corresponding to proton **e** has decreased. ^2D NMR shows that it has been replaced by a deuterium. **(c)** On the other hand, at the end of reaction B, the ^1H signal corresponding to proton **e** still integrates for 1 proton compared to proton a.

The partial NMR spectra shown were recorded in D_2O at 400 MHz and 298 K. Protons **a** and **e** are highlighted in green and red respectively.

13 Semi-empirical calculations

Table S2. Semi-empirical calculations of the total energy for the macrocycles (**M*n***) and catenanes (**C*n***) calculated using Avogadro, using UFF force field. These calculations do not take the solvent into account. However, the total energy of the catenanes **C*n*** already exhibits an odd-even trend. The macrocycles **M*n*** seem less sensitive to the odd-even effect.

n	**Total Energy of M*n* (kJ/mol)**	**Total Energy of C*n* (kJ/mol)**
4	481.269	1086.51
5	450.793	835.433
6	406.468	879.137
7	395.545	728.465
8	438.612	744.599
9	351.915	659.281

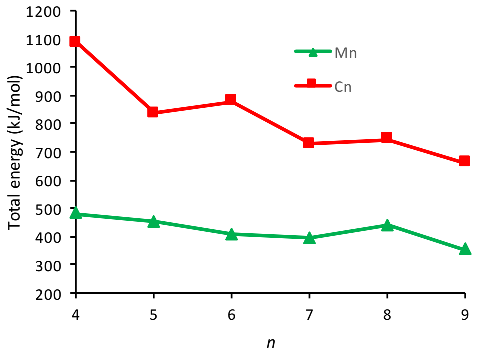

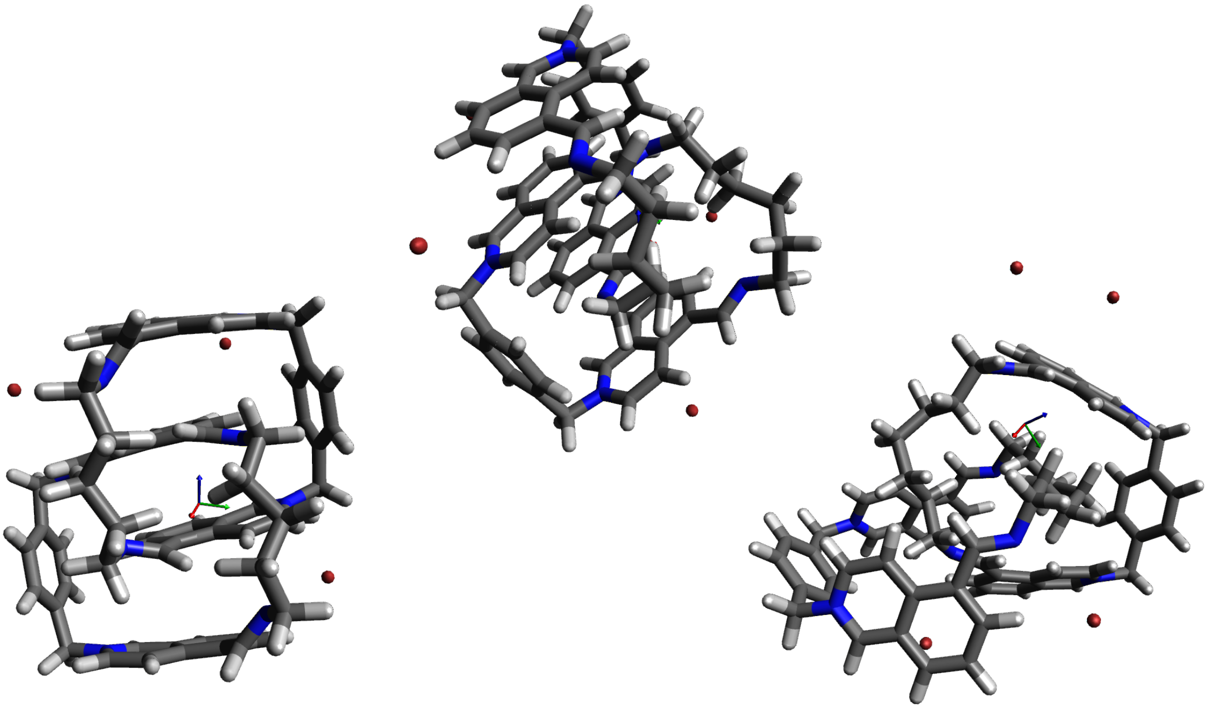

Figure S62. Representation of the minimized structures of **C**$_5$ (left), **C**$_6$ (center) and **C**$_7$ (right) generated with Avogadro (UFF force field, counter ion Br$^-$).
